# Antibiotic: a boon to mankind and doom to the environment - an insight on biological and non-biological degradation processes

**DOI:** 10.3389/fmicb.2026.1801898

**Published:** 2026-04-07

**Authors:** Baby Thankam Antony, Jabez William Osborne, Lincy Kirubhadharsini Benjamin

**Affiliations:** 1 Department of Bio Sciences, School of Bio Sciences and Technology, Vellore Institute of Technology, Vellore, India; 2 Department of Plant Pathology and Entomology, VIT-School of Agricultural Innovation and Advanced Learning, Vellore Institute of Technology, Vellore, India

**Keywords:** advanced chemical oxidation, metabolomics, microbial remediation, physical techniques, phytoremediation

## Abstract

Antibiotics are antimicrobial agents that are used to treat infections caused by pathogens. Antibiotics are released from various sources such as pharmaceutical industries, hospitals and municipal waste along with medical waste in higher concentrations. About 20% of the antibiotics are absorbed by humans or animals during metabolism upon consumption and excess is released into the environment through excretion. Frequent exposure of antibiotics on living organisms can develop resistance in host and its residues can have ill-effects on the environment including disturbance to the microbes in the ecosystem, and can also harm the non-target organisms. The current article summarizes the application of non-biological conventional treatment processes such as physical and chemical methods for the breakdown of antibiotics. However, these methods have limitations such as formation of secondary by-products and sludge alongside incomplete degradation. Therefore, application of biological methods for the degradation is considered to be highly effective due to their reduced toxicity. There are several microbes such as *Escherichia coli*, *Pseudomonas aeruginosa*, and *Streptococcus pneumoniae*, which are identified to be degrading antibiotic while administered during treatment regimens. In recent years, OMICs technologies has been used as a tool to provide insights on understanding the mechanisms involved in antibiotic degradation apart from the conventional processes. Therefore, the current review also proposes hypothetical and logical framework that can aid the research community in development of strategies that could lead to enhanced degradation of antibiotics by combining physical, chemical and biological processes.

## 1 Introduction

The discovery of arsphenamine (salvarsan) by Paul Ehrlich marked the beginning of the antibiotic era and revealed the capacity of antimicrobial agents to target pathogens (Hutchings et al., 2019; Leanse et al., 2022). Over the past century, the discovery of antibiotic compounds has remarkably reached a substantial achievement, ranging from early therapies such as arsphenamine to more recent developments like tethered macrocyclic peptides (Hutchings et al., 2019; Zampaloni et al., 2024). This is primarily due to the rapid increase in bacterial infections which leads to the illnesses caused by a wide range of pathogens that impair physical and mental health. Studies have shown the clinical efficacy of antibiotics or antimicrobial drugs in the treatment of polymicrobial illnesses (Sartini et al., 2021).

Antibiotics have been used since the 1940s to treat acute and chronic infections, prevent infections during the post-operative period, and as a prophylaxis for immuno compromised and cancer patients (Bidoia and Montagnolli, 2021; Tyers and Wright, 2019). These are grouped into various classes based on their chemical structure, mode of action, and the type of etiological agents against which they are effective. Based on grouping, they are divided into penicillins, cephalosporins, tetracyclines, macrolides, aminoglycosides, quinolones, sulfonamides, glycopeptides, lincosamides, oxazolidinones, carbapenems, and glycylcyclines (Cetecioglu and Atasoy, 2018; Tran et al., 2019).

The usage and abuse of antibiotics in excess has resulted in the development of drug resistance in several individuals especially in developing countries which are considered to be regions where antibiotic waste management is inadequate, high population density and limited regulatory enforcements. It also leads to the release of antibiotic into the environment which causes disturbances to the various flora and fauna of the ecosystem (Urban-Chmiel et al., 2022). The presence of antibiotics not only kills the pathogenic organisms, it also affects the microbial richness and diversity of the beneficial microbes which plays a pivotal role in the biogeochemical cycles (Mathur et al., 2021) by inhibiting the synthesis of several micronutrients sourced through microbes. Therefore, in environments under antibiotic stress, it is necessary to maintain the homeostasis of the soil nutrients which can be achieved using antibiotic degrading microbes (Thoms et al., 2021).

Several studies have reported the identification of microbes capable of degrading and utilizing antibiotics as sole carbon source has been reported in the past decade (Chen X. et al., 2023). The mechanisms involved in antibiotic resistance through enzymes like beta lactamases and metallo specific β lactamases and proteins that help in efflux pump (Rocha et al., 2019) such as AcrB (Zwama et al., 2017), have also been elaborated. Some of the microbes that harbors the above genes and enzymes include *Escherichia coli, Pseudomonas* sp., etc., could effectively degrade the antibiotics (Biondo, 2023). It is observed that not only bacteria possesses the ability to degrade but eukaryotes such as fungi, algae and plants are also shown to degrade antibiotics. Similar to bacterial mechanism, fungi like *Aspergillus niger, Trichoderma* sp., *Penicillium oxalicum* also exhibits the degradation ability (Arun et al., 2023). Algae such as *Chlorella vulgaris*, *Scenedesmus obliquus*, *Spirulina* sp., and few other cyanobacterial species are known to produce enzymes that can break down antibiotics (Huang et al., 2023). Recently, studies have reported the application of plants for the breakdown of antibiotics through the process of phytoremediation. Plants such as *Pteris vittata*, *Lolium* sp., etc., have shown to possess degradation potential (Zhou et al., 2024). However, combining multi-biosystems for the degradation could be more effective and can have a positive impact on the ecosystem (Babuponnusami et al., 2023).

Recently, omics technologies have proven to be effective in understanding the molecular mechanisms which helps in the prediction of pathways and it has also been employed in identifying the microbial functional dynamics of a particular ecological niche (Dai and Shen, 2022). Metabolomics is predominantly used in the detection and quantification of wide spectrum of antibiotics and their degraded metabolites. Development of metabolomics has significantly increased the understanding of mechanisms during recalcitrant degradation which has pioneered through KEGG (Kyoto Encyclopedia of Genes and Genomes) and other online platforms (Hong et al., 2024). This technology plays a crucial role in identifying degraded metabolites by profiling intermediate molecules, hereby elucidating degradation pathways through the valuation of toxic by-products. The advanced omics approaches such as metagenomics, metatranscriptomics and metaproteomics offers a framework to identify the microbial profile, functional gene, their expression, and the changes in expression under antibiotic stress. These omics technologies enhance mechanistic understanding of antibiotic biodegradation pathways (Dai and Shen, 2022).

**FIGURE 1:**
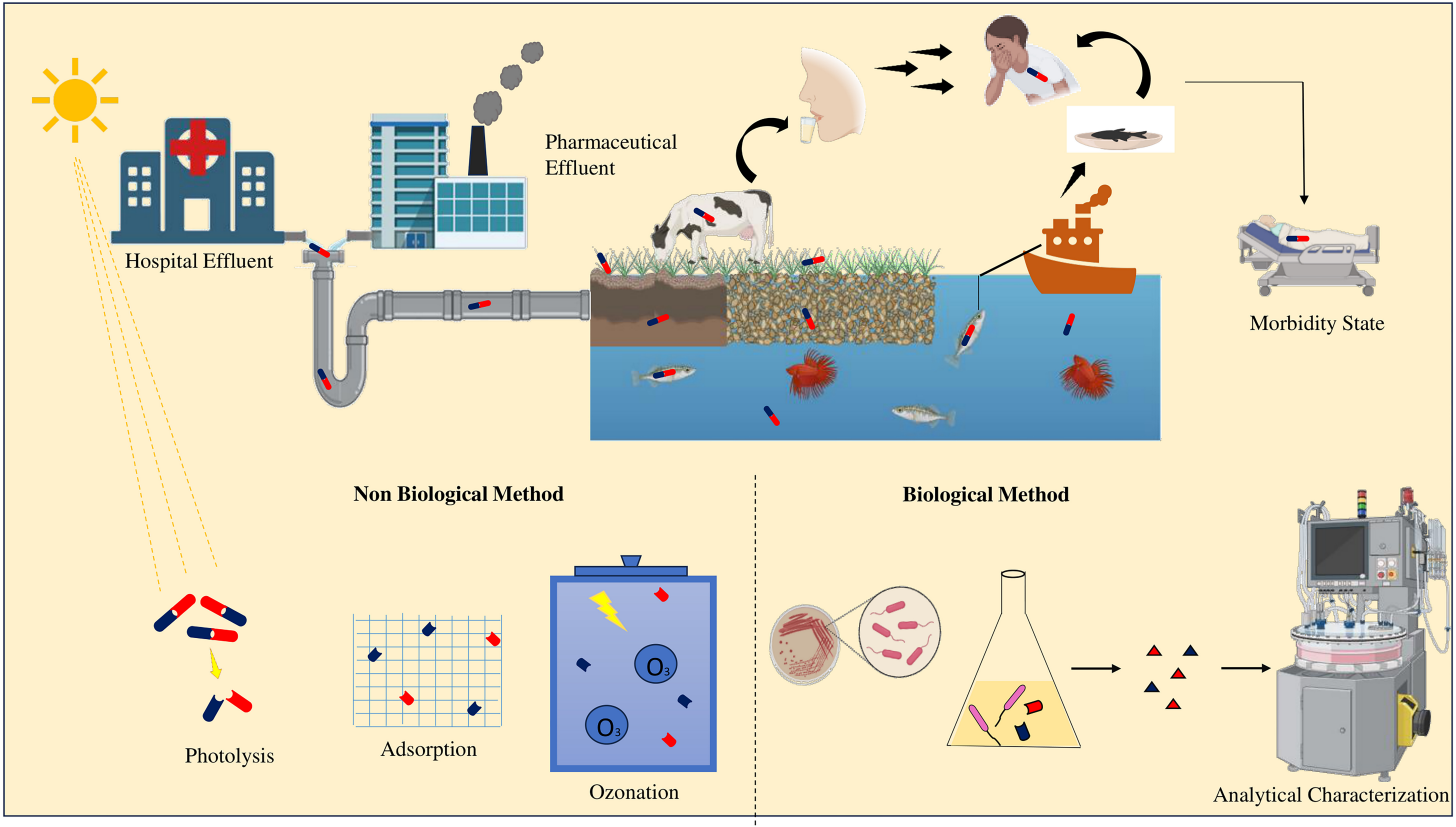
A graphical abstract showing environmental dissemination of antibiotics and resistance transmission to animals and humans: Morbidity risks, mitigation through biological and non-biological method and characterization by high-throughput sequencing approaches.

Demand and overuse of antibiotics have led to environment polluted with antibiotics and are hence considered as contaminants of emerging concern (Chaturvedi et al., 2021b). Bacteria exposed to these antibiotic residues can develop resistance genes making drugs ineffective for treatment of infectious diseases (Okaiyeto et al., 2024). This necessitates the use of higher doses or newer classes of antibiotics. Bioremediation of antibiotic is by specific enzymes such as beta lactamase and tetracyclinease and non-specific enzymes like hydrolase, carboxylase, dehydrogenase and esterase that leads to the breakdown of antibiotics (Cherazard et al., 2017) and similar mechanisms are also observed in fungi and algae (Randhawa et al., 2021; Figure 1).

Phytoremediation is another strategy that employs plants like hyperaccumulators for the uptake of antibiotics from soil through phytoextraction and rhizofiltration enabling the accumulation of antibiotics inside the plant system (Khan et al., 2024). This review provides a critical comparison of various methods for eliminating antibiotics from the environment and evaluating their efficiency of degradation by physical separation, chemical (advanced chemical oxidation method) and biological degradation by bacteria, fungi and algae. Further the efficiency of individual processes or their integration with traditional conventional techniques has also been provided.

Though various studies have reported and discussed on the removal or degradation techniques for antibiotics, there is a lack in the updation of recent developments in the antibiotic degradation. Modified degradation methods that integrate both conventional, as well as biological methods to control and minimize antibiotic is the need of the hour. Further research is required to identify combinations of conventional and biological treatments that can provide sustainable, cost- effective, complete remediation, while simultaneously ensuring the removal of antibiotic resistance genes and reuse of soil and water resources. The objectives of this review are as follows:

- A comprehensive reference for the recent knowledge on antibiotic classifications, their generations and mechanisms along with exploring their antibiotic resistance mechanisms and their respective genes responsible for resistance
- Advantages, disadvantages and efficiency of conventional antibiotic removal techniques such as physical, chemical and advanced chemical oxidation
- Study the biological strategies, particularly plant and microbial enzymatic actions involved in degradation and removal of antibiotics along with their merits, drawbacks and rate of efficiency
- Focus on the importance of metabolomics in understanding degradation pathways and the influence of degraded metabolite on environment and to outline future possibilities and challenges in mitigating environmental antibiotic pollution.
- Bridges the research gap by consolidating recent literature across degradation techniques with their novel and mechanistic insights, proposing hybrid treatment to yield better and efficient degradation incorporating OMICS as a tool.

## 2 Materials and methods

The review process has been segmented into three phases: initial stage holds the need for the study, its scope and methodology, the second stage involves search strategy, study selection, data extraction and synthesis and the final stage focuses on reporting the selected studies within a comparative framework and providing conclusions.

### 2.1 Literature search strategy

A total of 314 peer-reviewed articles were systematically examined and extensive literature search was conducted across major scientific databases including ScienceDirect, PubMed, Google Scholar, Scopus, Web of Science, Wiley Online Library and Embase. The primary literature search focusing on antibiotic degradation through biological strategies, mechanisms involved, metabolites identified and hybrid technologies of non-biological and biological studies were collected from 2000 to 2025 to ensure the inclusion of recent scientific developments, whereas early publications from 20th century were selectively incorporated to provide historical context of discovery, evolution and early clinical use of antibiotics. The review encompasses studies published from the late 1940s to outline the evolution of antibiotics. Key search terms comprised “antibiotic degradation,” “bioremediation,” “physical removal technique,” “chemical methods,” “chemical reactions in antibiotic degradation,” “advanced chemical oxidation,” “biological degradation of antibiotics,” “microbial degradation,” “enzymatic degradation by bacteria,” “fungal degradation of antibiotics,” “algal remediation,” “phytoremediation,” “wastewater treatment,” “metabolomics in antibiotic degradation,” “antibiotic degraded metabolites,” “environmental antibiotic pollution and their sources.” Boolean operators (OR/AND) were strategically applied to optimize the selection of relevant articles for this review (Chakraborty et al., 2025).

### 2.2 Study identification and selection

Following a comprehensive literature search, all identified records were transferred to Mendeley reference manager and duplicates were removed. Titles, abstract and keywords of the remaining studies were screened by all authors to eliminate irrelevant studies for this review. Study selection was based on inclusion and exclusion criteria. Any disagreements arising during selection process were resolved through discussion and consensus.

### 2.3 Inclusion criteria and exclusion criteria

Inclusion criteria used for the studies were reported experiments and published reviews detailing antibiotic resistance and degradation of antibiotics in wastewater, soil and aquatic environments. Additional criteria included were removal techniques by non-biological methods and degradation or accumulation studies by biological approaches. Research articles that explained antibiotic degradation by bacteria, fungi, algae and plants were preferentially included. Articles were prioritized if they showed high degradation efficiencies, novel mechanisms, details regarding by-products, identified genes that encoded for the specific and non-specific enzymes production which were responsible for degradation mechanisms. Studies showing both the parent antibiotic compound and its identified degradation metabolites, along with a detailed explanation of underlying mechanism were specifically selected. Articles involving metabolomics for degradation pathway derivation and other emerging technologies in the field of antibiotic remediation were also considered. Foundation of the article was built upon peer-reviewed journals, standards and regulations by organizations like World Health Organization (WHO), Environmental Protection Agency (EPA), Food and Drug Administration (FDA) and European Medicines Agency (EMA) published in English. Studies published in languages other than English without adequate translation, conference papers lacking full data, model based study without validation, duplicate publications and research that lacked quantitative outcomes were excluded from this review.

### 2.4 Data extraction and quality assessment

The study extracted key data including antibiotic classes, generations, mechanisms of action, point and non-point sources, restriction mechanisms, degradation methods and their efficiency, associated microorganisms and plants, reported antimicrobial resistant genes, physical membranes, chemical agents and their reactions, degradation products. Multiple databases were employed to minimize bias, enabling cross validation of findings from independent studies. Preference was given to the studies that used analytical techniques for confirming authenticity. Removal efficiencies of each method were documented along with their respective advantages and limitations. Certain approaches have drawbacks like incomplete degradation, substantial operational and management cost, sludge formation, production of toxic by-products, prolonged degradation times, thereby ruling out any overly optimistic interpretation.

### 2.5 Data synthesis

This review exclusively considered articles published in high-impact and reputed peer reviewed journals. Explicit contradictions and gaps in the existing literature were identified to guide our study in addressing the differences. The literature synthesis concerning the mitigation of environmental antibiotic contamination, begins with an examination of antibiotic fate in the environment, tracing their journeys from various sources into ecosystems. This explains their harmful effect on soil, water, human gut microbiota, human organs and human body (Patangia et al., 2022). Physical, chemical and advanced chemical oxidation have been explored with the limitations and challenges (Zhang et al., 2023; Ren et al., 2024). As a potential solution employing biological methods by bacteria, fungi, algae and plants along with their enzymatic reactions and uptake mechanisms were prioritized for the removal or degradation of antibiotic residues and antibiotic resistant genes and thereby safeguarding ecological and public health (Li and Nikaido, 2016).

## 3 Antibiotics from the past to present

The constant evolution of antibiotics has been driven by the pressing challenge of bacterial infections and antimicrobial resistance. Progress in this field has been guided by the principles of efficiency, specificity, safety and resistance management (Barathe et al., 2024). Arsphenamine, an early organoarsenic compound was used in the treatment regime of syphilis, had a relatively simple chemical structure with limited functional groups, resulting in low molecular weight. However, its efficacy was limited with increased side effects such as allergic reactions, liver damage, and arsenic poisoning due to its lack of specificity (Stanton et al., 2020; Alissa et al., 2024).

Naturally synthesized first generation of penicillin consisted of penicillin G and penicillin V and there are other semi-synthetically produced second generation antibiotics which includes methicillin and oxacillin that acquired modification from their natural structure (Gandhi and Ingole, 2023). Cephalosporin is one of the most modified antibiotic class that has cephalothin and cefazolin, cefuroxime and cefoxitin, ceftriaxone and ceftazidime, and cefepime and ceftaroline as the first, second, third and fourth generations, respectively (Lima et al., 2020). The broad spectrum tetracycline and oxytetracycline antibiotics belong to the first generation of tetracyclines whereas doxycycline and minocycline are modified, hence fits into the second generation of tetracyclines (Lin and Kück, 2022). Whereas, first generation of macrolides comprises of erythromycin, followed by second generation antibiotics like clarithromycin and azithromycin after structural modifications (Nelson and Levy, 2011). The first known quinolone was nalidixic acid, and after incorporation of fluorine atoms the widely used fluoroquinolones such as ciprofloxacin and levofloxacin were derived (Kirst, 2013). Vancomycin being the first generation antibiotic teicoplanin and oritavancin were its second class (Pham et al., 2021; Rubinstein and Keynan, 2014). Structural modifications in these compounds were carried out by adding functional groups, so as to serve as broad spectrum antibiotic which could resist enzymatic activity and other resistance mechanisms induced by the organisms (Table 1).

**TABLE 1:** Evolution of antibacterial agents over a century: discovery year, chemical class and structure, antibacterial spectrum, bacterial target sites and mechanisms of action.

Antibiotic profile	Chemical structure	References
Arsphenamine (1910) • Synthetic organoarsenic chemotherapeutic agent • Effective against *Treponema pallidum*	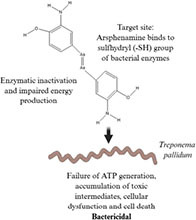	(Dowling, 1973)
Penicillin (1928) • Beta-lactam antibiotic • Effective against most gram-positive bacteria (*Steptococcus* sp., and *Staphylococcus* sp.) and some gram-negative bacteria (*Neisseria* and *Treponema pallidum*)	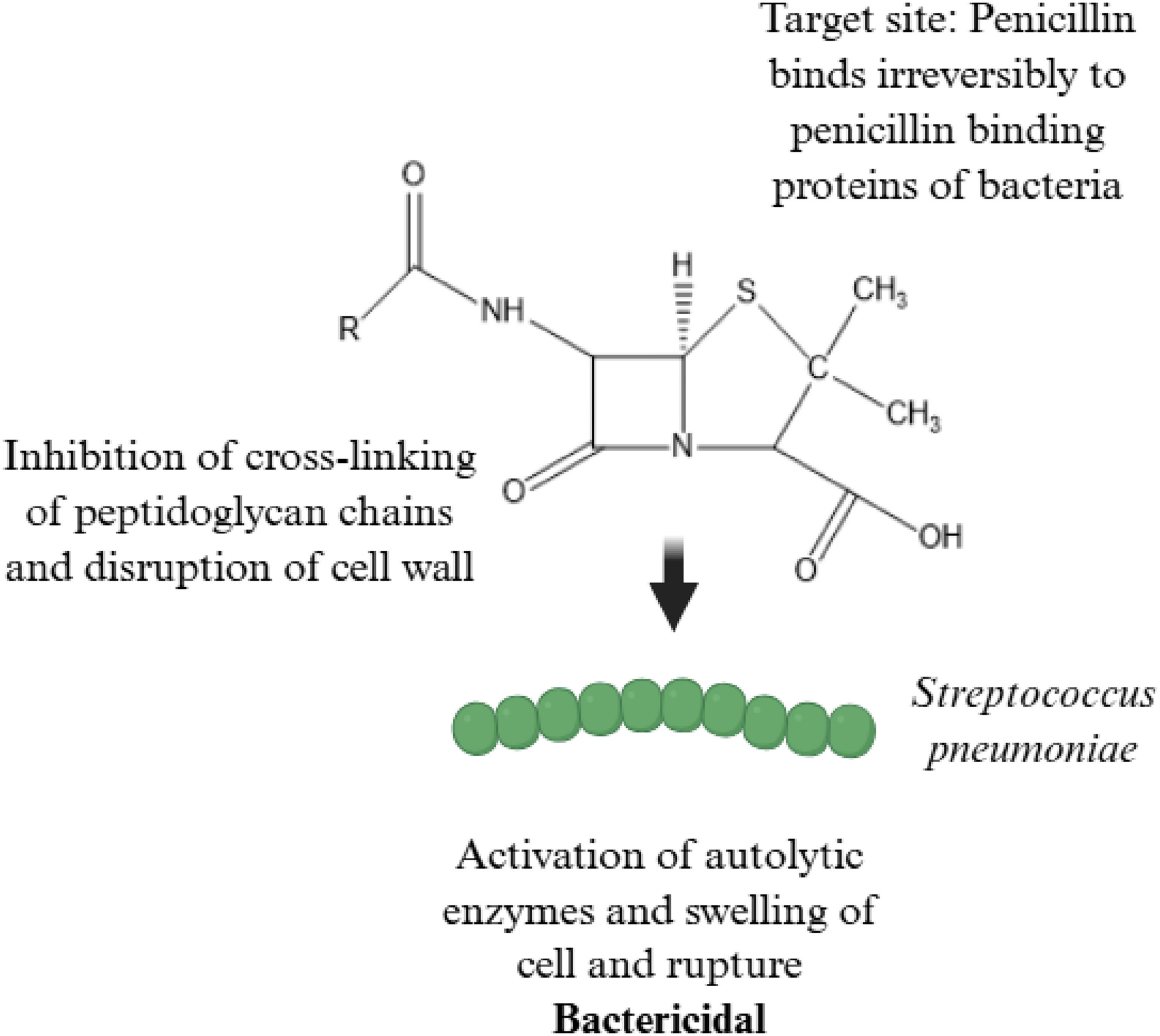	(Derderian, 2007; Castle et al., 2007)
Tetracycline (1948) • Tetracycline antibiotic • Effective against Gram-positive bacteria (*Streptococcus pyogenes*), gram-negative bacteria (*Escherichia coli*) and atypical bacteria (*Mycoplasma pneumoniae*)	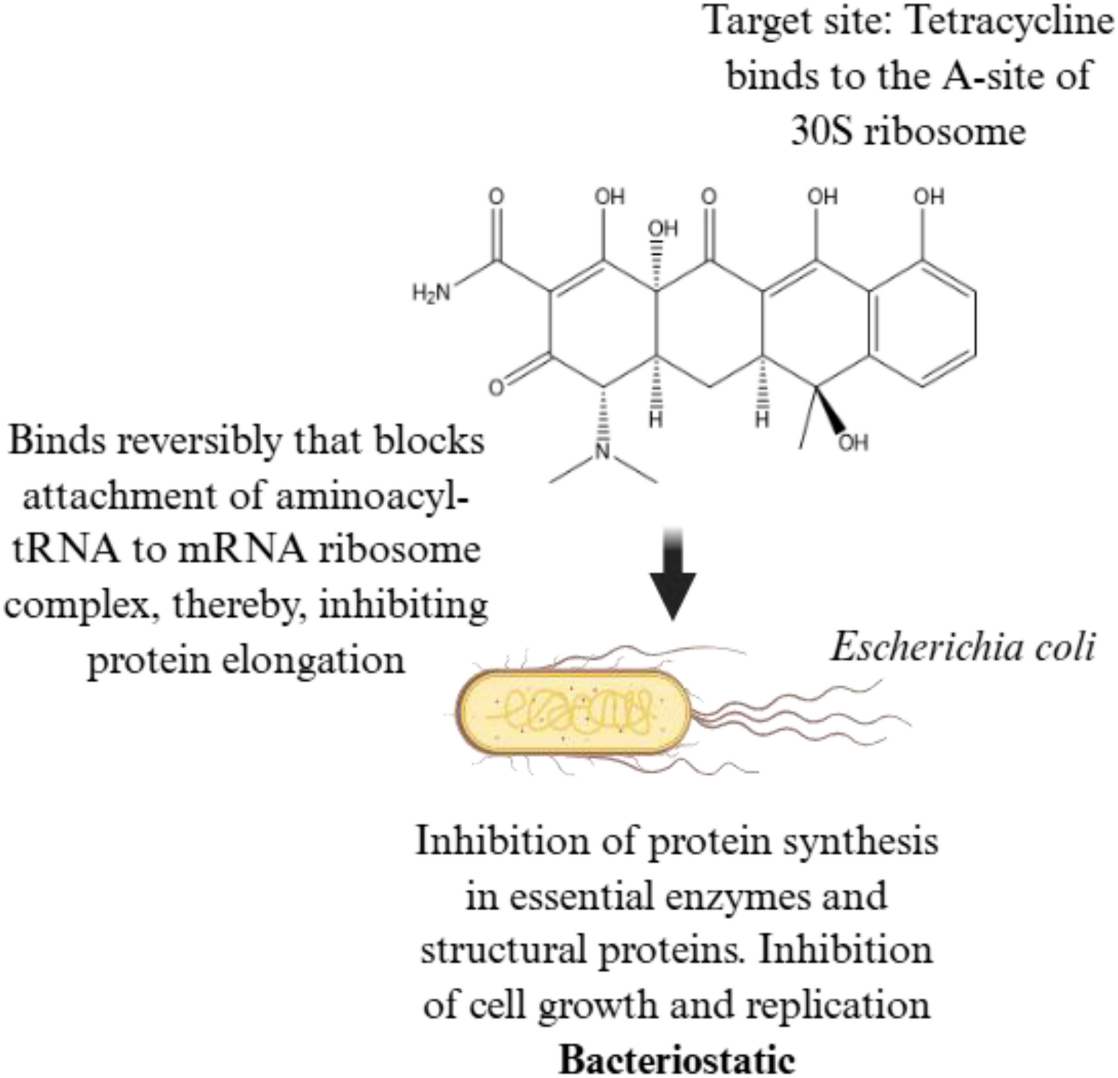	(Chopra and Roberts, 2001; Aminov, 2010)
Erythromycin (1952) • Macrolide antibiotic • Effective against Gram-positive bacteria (*Staphylococcus aureus*), gram-negative bacteria (*Neisseria gonorrhoeae*) and atypical bacteria (*Chlamydia trachomatis*)	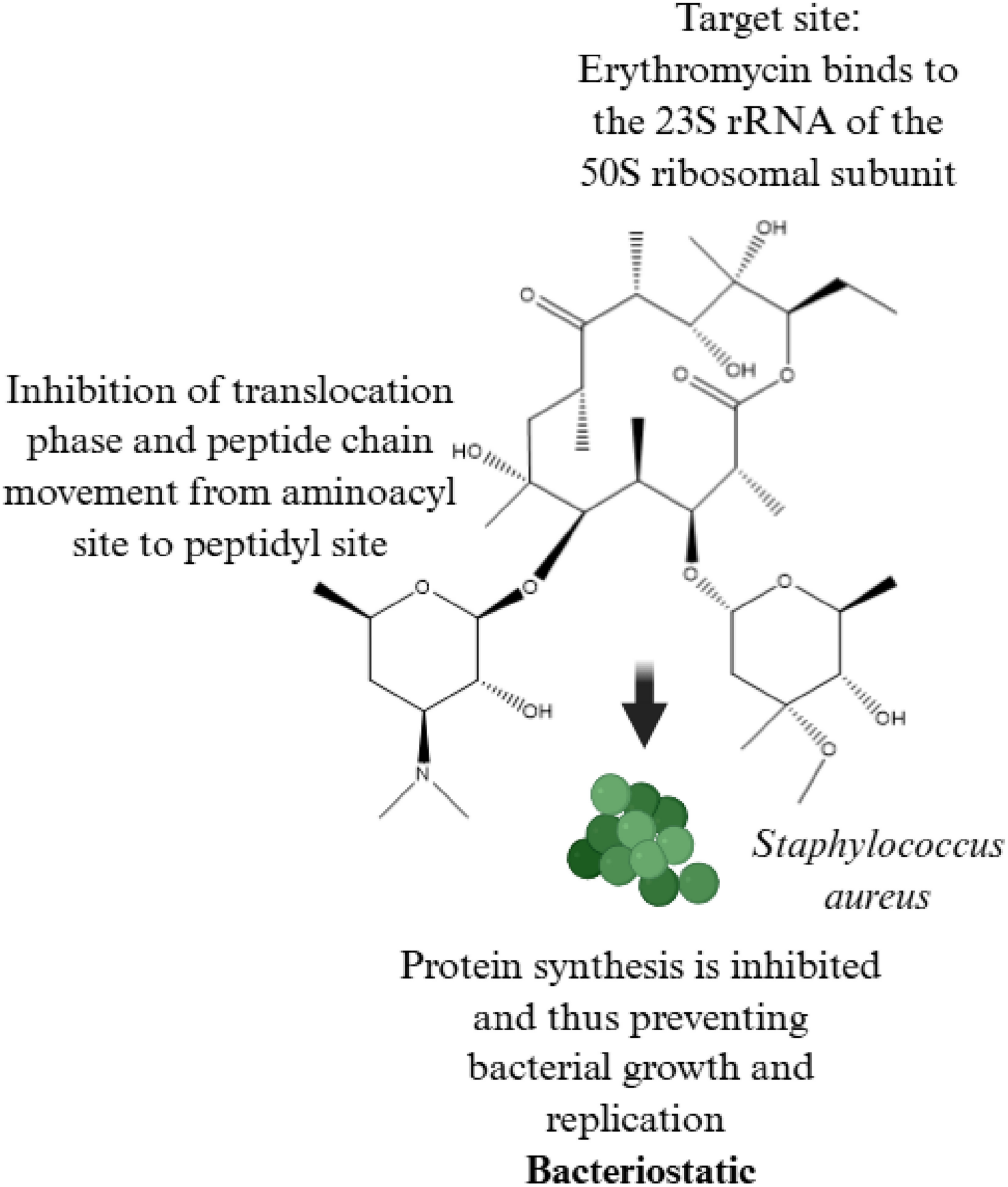	(Garrod, 1957)
Vancomycin (1953) • Glycopeptide antibiotic • Effective against Gram-positive bacteria (Methicillin resistant *Staphylococcus aureus*, *Enterococcus faecalis* and *Clostridioides difficile*)	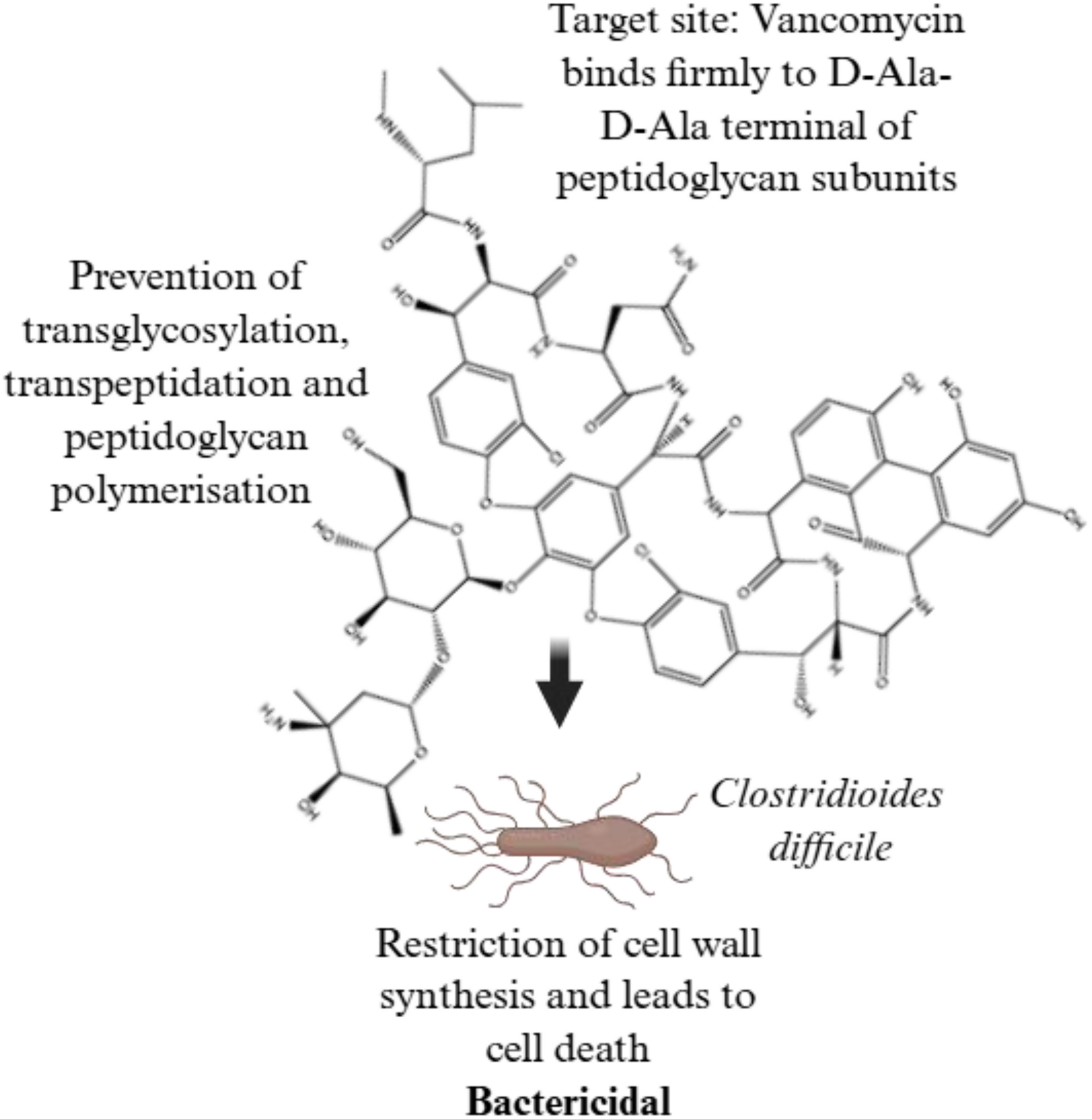	(Kwon et al., 2017; Blaskovich et al., 2022)
Nalidixic Acid (1962) • Quinolone antibiotic • Effective against: *Escherichia coli*, *Klebisella pneumoniae*, *Salmonella enterica*	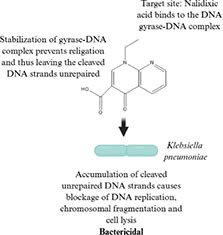	(Cederberg et al., 1974; Pham et al., 2019)
Imipenem (1980) • Carbapenem (Beta-lactam antibiotic) • Effective against: Gram-positive bacteria (*Staphylococcus* sp. and *Streptococcus* sp.) Gram-negative bacteria (*Pseudomonas aeruginosa* and *Haemophilus influenza*)	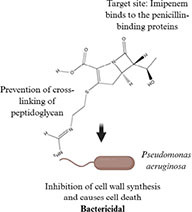	(McLeod and Lyon, 1985; Bricchi et al., 2020)
Zosurabalpin (2020) • Tethered macrocyclic peptide • Effective against: Multidrug-resistant organisms, carbapenem-resistant strains and specifically *Acinetobacter baumannii*	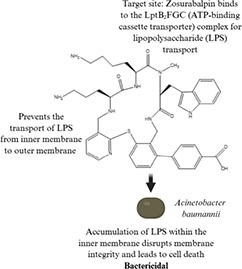	(Schüttel, 2023; Rathman and Del Valle, 2022)

On the other hand, tethered macrocyclic peptides represents a new class of drugs that have been recently discovered. As compared to Salvarsan, zosurabalpin, a recently developed experimental drug belonging to the tethered macrocyclic peptide family has been identified which is a large complex molecule which contains carboxyl, amine, hydroxyl, and diverse side chains consisting of amino acids with multiple cyclic structures, often referred to as macrocyclic compounds (Schüttel, 2023). Three-dimensional arrangement of these molecules allows them to precisely fit into the target receptor sites and due to their target specificity, they do not exhibit off-target effects (Rathman and Del Valle, 2022). All the antibiotics mentioned above are not completely utilized or broken down by the host and hence it is reported to be released through excretion into the environment.

## 4 Effect and consequences of antibiotics to the ecosystems

**FIGURE 2:**
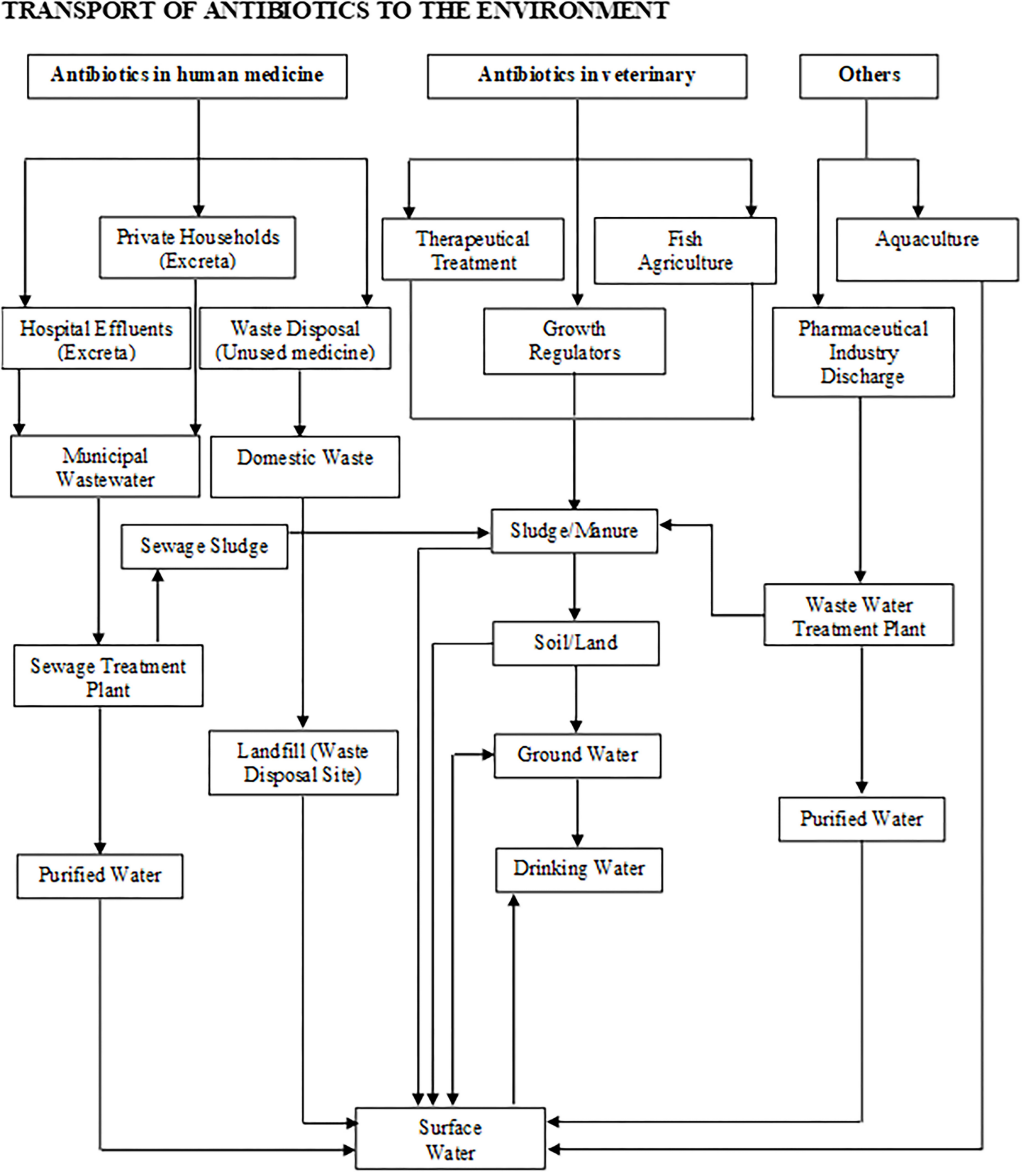
The major anthropogenic sources, fate and transport of antibiotics to the surface and ground water.

Antibiotics are extensively employed in the treatment of animal and human infections. These when enter into the ecosystem affects the unsaturated zones (vadose) and reach the saturated (aquifer) zone through percolation in soil due to its low density (Jayalakshmi et al., 2017; Kuppusamy et al., 2018). These antibiotics mostly reach the surface from urine or faeces of patients treated with antibiotics and in some cases, antibiotics are released through the effluents and sludge of pharmaceutical industries (Polianciuc et al., 2020; Wang et al., 2017; Wohde et al., 2016; Figure 2). Few micro gram per liter or nano gram per liter is sufficient to induce multi-resistance in microorganisms such as bacteria. The WHO recognized antibiotic residue and antimicrobial-resistant bacteria as one among the top ten priorities in the global health threats (Gray and Wenzel, 2020).

Under a controlled study (Gaikowski et al., 2003), dietary feed dosage of oxytetracycline in *Morone saxatilis* (<413 ppm/day) (Limbu et al., 2021), *Oncorhynchus mykiss* (< 4,000 ppm/day), *Salmo salar* (<308 ppm/day) (Bojarski et al., 2020), *Oreochromis niloticus* (<3 ppm/day) (Limbu et al., 2020) resulted in decreased growth, reduced feed intake, spinal deformities and short life span respectively. Exposure of oxytetracycline (<900 ppm/day) led to delayed hatching of embryos and absence of swim bladder in *Danio rerio* (Oliveira et al., 2013). Studies have also reported certain aquatic organisms such as *Cyprinus carpio* upon exposure of tetracycline (<0.2 ppm/day) showed malformation of tail (Escobar-Huerfano et al., 2020), *Clarias gariepinus* treated with chloramphenicol (<0.6 ppm/day) exhibited irregular swimming pattern (Nwani et al., 2014) and *Danio rerio* in response to gentamycin (<3,000 ppm/day) resulted in acute renal failure (Hentschel et al., 2005).

The uptake of residual antibiotics including doxycycline and tetracycline by plants interferes with the physiological processes and causes toxic effects on plants (Wang et al., 2017). Doxycycline (<25 ppm) showed reduced growth in *Arabidopsis thaliana* and *Triticum aestivum* when exposed to tetracycline (<0.2 ppm) exhibited retarded growth. Decreased root elongation in *Daucus carota* was observed (<5 ppm) due to the alteration in the photosynthetic gene activity, inhibition of organelle protein synthesis, impaired mitochondrial function, decreased cell division, and reduced oxidative stress reaction mechanism (Wang et al., 2015). In the agricultural field, soil enriched with manure contaminated with antibiotics could hinder seed development or reduce biological matter eventually leading to decreased crop production (Minden et al., 2016).

Terrestrial animals are regularly treated with low dosages of antibiotics such as tetracycline and macrolides in their feed to boost their growth and body weight. A study reported that poultry and cattle livestock, administered with tetracycline has accumulated residues (>8 ppm) in their body tissues including liver, kidney and muscle. This elevated residual concentration were found to be associated with pathological alterations such as inflammation of organs, hepatic enzyme alteration and accumulation of fatty acids in liver cells, tubular degeneration and impaired filtration in kidney (Enshaie et al., 2025). Although fluoroquinolone residues are generally detected at lower concentrations in animal tissues, their chronic effects, especially ciprofloxacin (<0.3 ppm) and enrofloxacin (<0.5 ppm) leads to disruption of mitochondrial DNA replication which in turn affects lipid peroxidation and apoptosis of cells present in liver and kidney. Furthermore, in juvenile animals, these antibiotics can impair cartilage formation and causes cartilage erosion (Oladeji et al., 2025). A single case of antibiotic-associated morbidity has been reported in free living vultures. However, unlike non-steroidal anti-inflammatory drugs such as diclofenac, antibiotics have not linked to large population mortality and therefore, a clear definite toxicological mechanism has not been established (Ives et al., 2022). Another major problem associated with antibiotic among the microbial community is the development of antibiotic resistance in microorganisms inherited from generation to generation by transfer of genetic elements through natural methods of gene transfer (Lerminiaux and Cameron, 2019). Studies have reported that genetic material is transferred from a bacteria to another and this bacteria which is involved in symbiosis with animals or humans, carry the resistance and continues to persist within the host (Hotopp, 2011; Vinayamohan et al., 2022). Therefore, horizontal gene transfer between organisms at different trophic levels leads to the development of antibiotic resistance from micro to macro organisms.

### 4.1 Effects of antibiotic on gut microbiota

Antibiotics or antimicrobial agents are known to treat millions of lives, till recent days, however their negative effects on gut microbial diversity are recently being reported (Yang et al., 2021). In cases, where the pathogen is unknown, physicians prescribe a broad spectrum of antibiotics, which can drastically reduce the number of beneficial organisms residing in the gut. According to a study by Ainonen and other researchers, individuals with impaired immune response and children are more vulnerable to the negative effects of antibiotics due to their reduced immune response (Ainonen et al., 2022). Orally ingested antibiotics directly interact with the gastrointestinal tract and the associated microbiome. Consequently, they have a more pronounced impact on these regions as compared to other organs and tissues (Mathur et al., 2021; Table 2).

**TABLE 2:** Antibiotic induced disruption of beneficial gut microbiota and intestinal homeostasis.

Antibiotics	Effects	References
Clindamycin	Increases *Clostridium difficile* Significant decrease in *Bacteroides* species	(Buffie et al., 2012)
Ciprofloxacin	Affects the *Enterobacteriaceae* family, including both harmful and beneficial organisms	(Kourbeti et al., 2010)
Amoxicillin clavulunate	Total reduction in the population of *Bifidobacterium*, partial removal of *Clostridium* clusters IV and XIVa, *Bacteroides fragilis* is predominant before antibiotic treatment whereas *Bacteroides distasonis* is dominant after antibiotic treatment	(Stavroulaki et al., 2021)
Clarithromycin Metronidazole	Upon the antibiotic treatment, firmicutes and proteobacteria becomes dominant and there is a substantial decrease in actinobacteria and bifidobacterium	(Brüssow, 2020; Greenstein, 1993)
Cefprozil	Increase in *Lacnoclostridium bolteae*	(Raymond et al., 2016)

Healthy individuals harbors gut microbiota which belongs to the phyla such as Firmicutes (*Faecalibacterium*, *Ruminococcus*, *Lactobacillus*), Bacteroidetes (*Bacteroides*, *Prevotella*), Actinobacteria (*Bifidobacterium*) and a minor presence of other Proteobacteria. In such individuals beneficial gut bacteria produce butyrate which hold junction proteins firm and suppress inflammation (Raymond et al., 2016). Antibiotic-associated dysbiosis reduces butyrate level, compromising the integrity of junction and increases intestinal permeability. This facilitates passage of bacterial toxins to bloodstream that triggers inflammatory cytokines release which leads to tumor, obesity and digestive system disorders. Prolonged antibiotic consumption disturbs the immune balance by decreasing regulatory T cells and increasing IgE antibody production that leads to allergic reactions, asthma and autoimmune disorders (Akhremchuk et al., 2022; Sarasti et al., 2025). In some cases, antibiotics such as clindamycin, ciprofloxacin, ceftriaxone and amoxicillin can lead to over colonization of *Clostridium difficile* which causes antibiotic-associated diarrhea. Presence of antibiotics also play a significant role in the reduction of *Bifidobacterium* species which is a known beneficial gut associated bacteria (Hrncir, 2022). The duration of antibiotic therapy, the number of doses, the antibiotic class, age of the host, genetic susceptibility, and lifestyle have an impact on the changes in gut microbial community. Humans respond to antibiotics in different ways, and the recovery time vary from person to person (Sarasti et al., 2025).

### 4.2 Effect of antibiotics on human organs and cells

Prolonged and excessive use of antibiotics can have detrimental effects on various organs. These agents can generate and release reactive oxygen species and free radicals, leading to oxidative stress in the tissues and organs (Raymond et al., 2016). Structurally, a healthy gut has intact mucosal lining, well-developed villi, balanced mucus secretion and a rigid epithelial barrier. However, administration of antibiotic can cause thinning of the mucus layer, blunting of villi, loosening of epithelial barrier, indicating increased permeability to infectious organisms and inflammation (Hrncir, 2022). Furthermore, in conditions where digestive and immune functions are compromised or individuals suffering from gastric cancer, the horizontal transfer of antibiotic resistant genes to other organisms can occur and this can lead to increase in antibiotic resistance among the gut microbial population (Thrastardottir et al., 2022). The after-effects of antibiotics are not limited to the gut, they also have ill-effects on various organs and cells through body fluids. In the liver, excessive usage of amoxicillin-clavulunate, rifampin and erythromycin increases enzyme production, resulting in liver dysfunction. Antibiotics such as gentamycin, vancomycin, amphotericin B and cephaloridine can accumulate in the renal tubular epithelial cells, causing direct toxicity release of cytokines, leading to kidney inflammation (Morales-Alvarez, 2020). Cardiotoxicity is another significant problem, caused by erythromycin, azithromycin, ciprofloxacin and levofloxacin that blocks potassium channels in cardiac cells, which can prolong the QT (interval between Q and T wave on an electrocardiogram) interval and lead to arrhythmia (Schwartz et al., 2020). Certain antibiotics, such as doxorubicin, used as chemotherapeutic agents, can directly affect cardiac muscle cells. Additionally, antibiotics including penicillin, cephalosporin, metronidazole, ciprofloxacin, erythromycin, and isoniazid, can cross the blood-brain barrier and cause seizures, confusion, hallucinations, and dizziness by affecting brain cells (Patangia et al., 2022). These reports have shown the ill-effects caused by antibiotics in humans and it is the need of the hour to identify a suitable strategy that could be effectively used for the degradation of antibiotics.

## 5 Biological and non-biological methods in antibiotic removal

### 5.1 Preliminary step in antibiotic removal: physical method

Physical techniques for the removal of antimicrobial compounds and their active compound residues aim to separate or remove these substances through simple mechanisms that do not alter their chemical structure. Though these techniques are relatively cost-effective as compared to other non-conventional approaches, they are known to have limited effectiveness rates and one of the widely applied physical method is membrane infiltration that include microfiltration, nanofiltration, ultrafiltration, as well as forward and reverse osmosis (Li et al., 2019). Antibiotics removed through physical strategies are not degraded, rather are extracted and transferred to the disposal unit. These antibiotics are primarily separated based on their molecular size, charge, hydrophobicity and size exclusion properties. Antibiotic degradation and formation of by-products may occur through biological method, advanced chemical oxidation techniques, and physical methods when coupled with either biological or advanced chemical oxidation (Akhil et al., 2021; Le-Minh et al., 2010). Physical methods serve as a preliminary treatment approach, they aid in adsorption, advanced chemical oxidation or biodegradation methods for the effective clearance of separated antibiotics.

Microfiltration is based on the screening using membrane with a pore size ranging between 0.1 and 1.0 μm which can filter particles or organic molecules using static pressure (Thakre et al., 2024), hence preventing the passage of microorganisms (Li et al., 2019; Yin et al., 2021). A recent study showed 58.9–100% removal of antibiotics using a hybrid model microfiltration operated with reverse osmosis against various classes of antibiotics such as enrofloxacin, sulfamethazine, cefalexin, amoxicillin, lomefloxacin and ampicillin. However, it was inefficient in the removal of sulfonamides and trimethoprim (Qiu et al., 2021). In contrast, nanofiltration can provide improved removal efficiency as compared to microfiltration due to its smaller pore size which facilitates the removal low molecular weight antibiotics. Porosity of the nano-filtration membrane is confined to 0.2–2 nm which aids in retaining molecules with 80–1,000 Da. The most prevalent type of membrane modules are hollow tubes, spiral-wrapped configurations, plated frame designs and tube (Cristóvão et al., 2021). In a study, the application of Desal 5 DK nanofiltration membrane for the treatment of effluent reduced the microbial load by 98% and carbapenem resistant genes by 99.99% (blaKPC, blaOXA-48, blaNDM, blaIMP, blaVIM) and fluoroquinolone (qnrA, qnrB, and qnrS) (Oliveira et al., 2022; Song et al., 2022). Nanofiltration is highly effective for antibiotic removal, however ultrafiltration is generally used for separation to avoid fouling of the membrane.

Ultrafiltration membrane has a pore size of 0.001–0.2 μm and is mostly employed in drinking water treatment plants, where it has been shown to enhance the efficiency in removing beta-lactams, tetracycline, erythromycin and sulfonamide antibiotics by 21% on an average as compared to studies without ultrafiltration (Hampu et al., 2020). Fluoroquinolone antibiotics and antifungal drugs that are resistant to ozonation process has been reported to be removed using ultrafiltration (Li et al., 2018; Li L. et al., 2023). A modified ultrafiltration process showed micelle chemistry, where the surfactants generated micelles that could encapsulate antibiotics such as levofloxacin and sulfamethazine with a removal efficiency of 84.1 and 95.3%, respectively (Raval et al., 2024). Although ultrafiltration exhibits limited removal efficiency for low molecular weight antibiotics, it plays a significant role in separating particulate matter and microorganisms, thereby serving as an effective pre-treatment stage prior to forward or reverse osmosis for enhanced antibiotic removal.

Forward and reverse osmosis membranes has proven to be effective in antibiotic removal. It has a pore size of 0.0001 and 0.0005 μm that are selectively permeable and reverse osmosis differs from forward osmosis by their direction (Field et al., 2021). A bioreactor study showed the application of reverse osmosis with highest efficiency in the removal of trimethoprim, carbapenem, tetracycline and fluoroquinolone (∼95%) (Akhil et al., 2021), though antibiotic removal efficiency was higher, there are certain drawback associated in this membrane treatment technology. Some of it includes fouling of the membrane due to microbial biofilms which deteriorates the membrane, allowing the passage of water containing antibiotic resistant genes to reach the human opportunistic pathogens (Labella et al., 2021). In another study, steric hindrance and electrostatic interaction have been reported to be the major factors that controlled the rate of removal of charged antibiotics such as ciprofloxacin, norfloxacin, erythromycin and azithromycin (98% removal) from municipal and industrial wastewater using a nanofiber composite combined with forward osmosis (Sanahuja-Embuena et al., 2021). Steric hindrance normally has little effect on charged macromolecular or neutral antibiotics, whereas electrostatic contact has a major impact on charged micromolecular antibiotics (Zhang et al., 2020).

The application of nanomaterials and nanoparticles in the field of membrane infiltration technique has been increased to enhance the efficiency of membrane-based systems for the removal of antibiotics. Nanoparticles such as carbon nanotubes and silver nanoparticles can improve the flow rate of water through membrane, thereby enhancing the removal of antibiotics (Majeed et al., 2024). Membranes incorporated with carbon nanotubes has shown to exhibit remarkable mechanical strength with improved filtration of antibiotics like sulfamethazine and amoxicillin. Certain nanoparticles like graphene oxide are specific in nature and can alter the surface charge of membrane to remove specific antibiotics like beta-lactams and tetracyclines (Singh et al., 2023). Titanium dioxide nanoparticles are used in the hybrid model of membrane filtration coupled with other chemical or advanced chemical oxidation methods for the efficient removal of fluoroquinolones and macrolides (Zhang et al., 2023; Majeed et al., 2024). The use of nanomaterials can improve the efficiency of antibiotic removal, however, this may also generate residual nanomaterials and toxic intermediates. These intermediates and residues which arise concurrently with antibiotic by-products have the potential to leach into the water and gradually impact the environment (Ezeuko et al., 2021). The application of physical processes in antibiotic removal has been ineffective, as all the processes can only separate the antibiotic and not degrade.

### 5.2 Chemical treatment process for the remediation of antibiotic pollution

Conventional chemical methods employed for antibiotic removal include chlorination, redox reactions, precipitation and chemical adsorption whereas advanced chemical oxidation process include photolysis/heterogenous photocatalysis, ozonation, fenton, sonochemical and electrochemical oxidation methods. The primary distinction between these two treatments are their dominant degradation mechanism. In conventional method, their antibiotic removal or transformation occurs through direct molecular reactions such as electron transfer or selective oxidation without intentional radical generation. Though they rarely achieve complete mineralization, these methods are favored due to their simple, less energy demanding and dependence on direct chemical reaction (Cuerda-correa et al., 2019). In contrast, advanced chemical oxidation are specifically designed to degrade pollutants through *in situ* generation of highly reactive radical species, facilitating rapid and non-specific degradation. This method has been used in the treatment of municipal wastewater, polluted water bodies, and pharmaceutical industry effluents. However, these effective methods require higher initial, operational, and maintenance costs for their functioning (Akbari et al., 2021). Other operational and mechanism-based differences between these strategies are discussed in Table 3 (Pandis et al., 2022; Shi et al., 2022; Alrefaey et al., 2025).

**TABLE 3:** Distinguishing features of conventional chemical methods and advanced chemical oxidation processes in antibiotic treatment.

Criteria	Conventional chemical methods	Advanced oxidation processes
Degradation/removal mechanism	Electron transfer, selective oxidation or phase transfer process	Radical-driven oxidation by Reactive Oxygen Species (ROS)
Active oxidants	Molecular oxidants or surface interactions	Hydroxyl radicals, superoxide and other ROS
Radical generation	Incidentally generated (Cl_2_, Fe^2+^)	Specifically generated ($\cdot OH,O_{2}^{-}.$)
Oxidation strength	Moderate	Very high
Reaction specificity	More selective and functional group specific	Non selective oxidation
Mineralization potential	Usually removal or partial degradation	Better degradation compared to conventional chemical method
Reaction kinetics	Moderate	Faster due to radical-mediated
Target antibiotics	Limited effectiveness for persistent compounds	Highly effective for refractory and persistent antibiotics

#### 5.2.1 Conventional chemical approaches for antibiotic removal and degradation

##### 5.2.1.1 Antibiotic degradation by chlorination

Chlorination occurs when chlorine reacts with the functional group of antibiotics. There are three mechanisms involved in the generation of chlorine and the type of mechanism to be followed is dependent on the antibiotic structure and reaction conditions. In electrophilic chlorination, chlorine from chlorine gas, hypochlorous acid or N-chlorosuccinimide when added to an antibiotic inactivates their ability to bind with bacterial ribosomes. The reaction is as follows (1), where chloramphenicol reacts with chlorine to substitute the chlorine atom on the aromatic ring of chloramphenicol leading to the inhibition of protein synthesis in bacteria (Lin et al., 2022). In oxidative chlorination, hypochlorite ion (ClO^–^) generated from sodium hypochlorite (NaOCl) serves as both oxidant and chlorinating agent. This reaction is mainly observed in sulfur containing antibiotic like penicillin. An example for this reaction is provided in reaction (2) ClO^–^ reacts which alters the aliphatic chain of penicillin under acidic conditions and form chlorinated penicillin with Cl^+^ on sulfur group instead of hydrogen atom. This binding leads to the oxidation of sulfur group or disruption of beta-lactam ring which reduces the toxicity of antibiotic (Zhao et al., 2021). In radical chlorination, ultraviolet light interacts with chlorine gas, results in the formation of chlorine radicals. These highly reactive chlorine radicals can bind to the aliphatic chain, leading to a structural alteration of the antibiotic. This process can be explained by chemical reaction (3), where incorporation of chlorine radicals to the aliphatic chain of erythromycin leads to the disruption of macrolactone ring and this structural change prevents the antibiotic from binding to its target organism (Li W. et al., 2023). Though the chlorination method is economical and offers immediate result, it possesses a risk factor due to the presence of their chlorinated by-products. In such cases, chemical precipitation is applied to remove the dissolved contents into insoluble salts.


$$C_{11}\,H_{12}\,C\,l_{2}\,N_{2}\,O_{5}+C\,l_{2}\overset{F\,e\,C\,l_{3}}{⟶}C_{11}\,H_{11}\,C\,l_{13}\,N_{2}\,O_{5}+H\,C\,l$$ (1)

$$C_{16}\,H_{18}\,N_{2}\,O_{4}\,S+N\,a\,O\,C\,l\overset{a\,c\,i\,d\,i\,c\,c\,o\,n\,d\,i\,t\,i\,o\,n\,s}{⟶}C_{16}\,H_{17}\,N_{2}\,O_{4}\,S\,C\,l+N\,a\,O\,H$$ (2)

$$C_{37}\,H_{67}\,N\,O_{13}+C\,l_{2}\overset{U\,V\,l\,i\,g\,h\,t}{⟶}C_{37}\,H_{66}\,N\,O_{13}\,C\,l+H\,C\,l$$ (3)

##### 5.2.1.2 Antibiotic removal by chemical precipitation and redox reactions

Precipitation is a chemical process where a soluble substance in a solution reacts with another soluble or insoluble substance to form an insoluble compound, known as a precipitate. Antibiotics can also be removed through this process, as they typically react with metal ions such as Ca^2+^, Mg^2+^, Fe^3+^, and Cu^2+^ or reagents to form the insoluble-antibiotic complex. These precipitated antibiotic complexes can be physically separated from the solution by filtration, sedimentation or centrifugation process (Abed and Faisal, 2023). An example to show this precipitation reaction is explained in reaction (4) where tetracycline reacts with Ca^2+^ ions resulting in insoluble calcium-tetracycline complex (Hou et al., 2024). This method is known for its simple and selective nature with low energy requirement. However, incomplete degradation and formation of sludge are the major limitations, as the antibiotics are mostly immobilized in the precipitate which is further disposed or treated. In contrast to precipitation, which mainly results in transforming aqueous phase into solid, redox reactions facilitate antibiotic degradation by structural modification through electron transfer mechanisms.


$$C_{22}\,H_{24}\,N_{2}\,O_{8}+C\,a^{2+}\to \left(C_{22}\,H_{24}\,N_{2}\,O_{8}\right)\,C\,a$$ (4)

In redox reaction removal of antibiotic occurs through either oxidation or reduction which is specific in nature that provides targeted degradation of antibiotics. In oxidation reaction, generally addition of oxygen and removal of hydrogen takes place and removal of antibiotic through redox reaction is explained as follows, reaction (5) hydroxyl group on the quinolone structure of ciprofloxacin is oxidized into a carbonyl group in the presence of oxidizing agents. Ciprofloxacin typically inhibits the activity of DNA gyrase and topoisomerase IV which are the essential enzymes that are involved in bacterial DNA replication. However, as the hydroxyl group is replaced by the carbonyl group, this inhibitory mechanism is disrupted and the antibiotic loses its antibacterial activity (Bhatt and Chatterjee, 2022). In reaction (6), the carbonyl group present in the tetracycline is reduced into a by-product with alcohol group in presence of hydrogen molecule, resulting in the modification of chemical structure which negatively affects their interaction with bacterial targets (Ahmad et al., 2021). Redox reactions can be applied for wide range of antibiotics on both small and industrial scale. However, the approach demands high energy, high cost and degradation can be incomplete with the persistence of by-products.


$$C_{17}\,H_{18}\,F\,N_{3}\,O_{3}+O_{2}\to C_{17}\,H_{16}\,F\,N_{3}\,O_{4}+H_{2}\,O$$ (5)

$$C_{22}\,H_{24}\,N_{2}\,O_{8}+2\,H_{2}\to C_{22}\,H_{28}\,N_{2}\,O_{8}$$ (6)

##### 5.2.1.3 Antibiotic degradation by chemical adsorption

In chemical adsorption, the antibiotic is adsorbed onto carbonaceous adsorbents which has electrostatic force, pore filling, hydrogen bond, and hydrophobic behavior which aids adsorption ability (Huang et al., 2022). Some of these adsorbents may require a chemical or thermal treatment before they are applied to increase their surface areas and adsorption capacity (Dutta and Mala, 2020). The rate of tetracycline, sulfamethoxazole, norfloxacin, amoxicillin and erythromycin elimination has reported to be enhanced by natural biochars, biochar composites, and engineered biochars (Krasucka et al., 2021). In a study, a biosurfactant (sophorolipid) produced by *Candida bombicola*, was found to increase the interaction between adsorbent (fly ash with sophorolipid) and adsorbate containing tetracycline showed the enhanced adsorption of antibiotic to be 55.57 mg/g (Ren et al., 2024). Another major development in adsorption technology is the triple network combination of carbon nanotubes, graphene oxide, and sodium alginate in the creation of a three-dimensional hydrogel with higher surface area to adsorb higher concentrations of antibiotics such as chloramphenicol, clarithromycin, amoxicillin, penicillin, sulfamethoxazole and ciprofloxacin to the surface (Ma et al., 2020). Polymer of Intrinsic Microporosity-1 is used to remove combination of trimethroprim with sulfamethoxazole, sulfonamides, fluoroquinolone and beta-lactams from aquatic environments and this application is regulated by surface and pore-filling adsorption. Electrostatic interactions between aromatic rings and charged functional groups, as well as the formation of hydrogen bonds between the adsorbent and adsorbate, are the changes that facilitate the removal of antibiotics (Alnajrani and Alsager, 2020).

Nanomaterials are also used in the chemical method to enhance their removal and degradation efficiency (Zhang et al., 2023). In radical chlorination, metal oxide nanoparticles like TiO_2_, Fe_2_O_3_ has been used in combination with UV radiation to enhance degradation efficiency, whereas in case of redox reactions zero-valent iron or TiO_2_ can be used to increase the effectiveness of degradation (Shree et al., 2023).

#### 5.2.2 Advanced chemical oxidation processes for the degradation of antibiotics

##### 5.2.2.1 Antibiotic degradation by photolysis and heterogeneous photocatalysis

**FIGURE 3:**
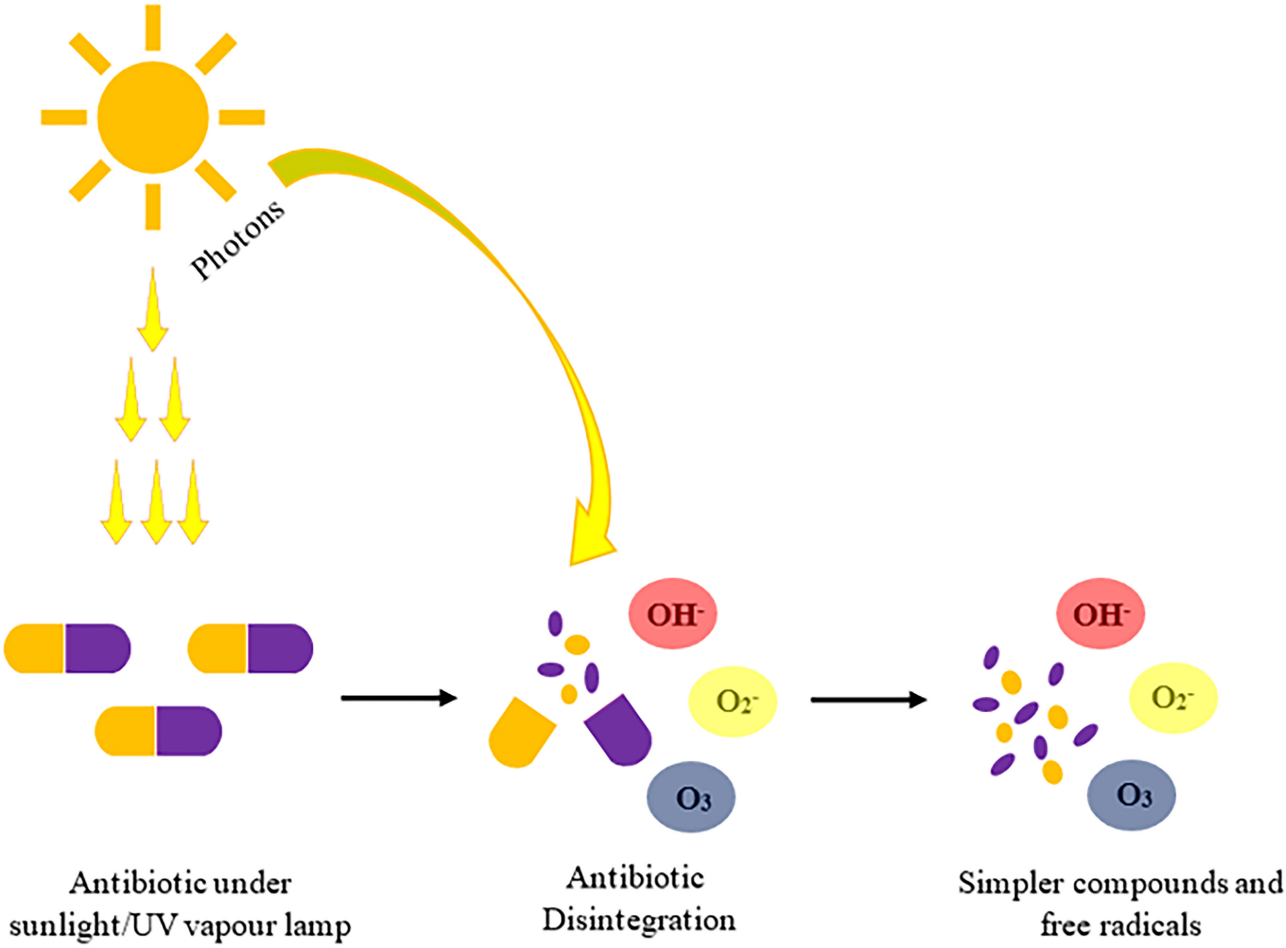
Mechanism of antibiotic photodegradation and radical formation under solar/UV irradiation.


Photolysis involves the application of light energy (photons) which can be absorbed by molecules, causing them to break down into simpler units. Typically, ultraviolet wavelength ranging from 200 to 400 nm has been reported to cause photolysis and photocatalysis. However, commercial UV lamps emits radiation of 185 nm which leads to a more enhanced production of reactive species. Reaction (7)–(10) shows various possible mechanisms involved in the degradation of ciprofloxacin by photolysis based on their photon energy, oxygen presence and pH. Reaction (7) explains decarboxylation reaction, where desethylene norfloxacin and carbon dioxide are formed (Lin et al., 2018), reaction (8) describes oxidation of piperazine ring that leads to the production of desethylene ciprofloxacin and methylamine upon absorption of light (Tozar et al., 2021) reaction (9) shows the mechanism of hydroxylation, where addition of hydroxyl radicals leads to the formation of hydroxylated ciprofloxacin (Lin et al., 2023; Ren et al., 2023), reaction (10) explains the influence of light (hν) to form desfluoro ciprofloxacin which removes fluorine from the parent compound (Tian et al., 2023). Under prolonged exposure to light and oxygen, complete degradation of ciprofloxacin was achieved which resulted in the production of formic and acetic acids along with the release of carbon dioxide and water (Abdulrahman et al., 2022; Figure 3).

$$C_{17}\,H_{18}\,F\,N_{3}\,O_{3}\overset{\begin{array}{c} h\,\nu \end{array}}{⟶}C_{16}\,H_{16}\,F\,N_{3}\,\begin{array}{c} O \end{array}+C\,O_{2}$$ (7)

$$C_{17}\,H_{18}\,F\,N_{3}\,O_{3}\overset{\begin{array}{c} h\,\nu \end{array}}{⟶}C_{16}\,H_{15}\,F\,N_{2}\,O_{3}+N_{2}\,C\,H_{3}$$ (8)

$$C_{17}\,H_{18}\,F\,N_{3}\,O_{3}+\begin{array}{c} . \end{array}\,\begin{array}{c} O\,H \end{array}\to C_{17}\,H_{18}\,N_{3}\,O_{4}$$ (9)

$$C_{17}\,H_{18}\,F\,N_{3}\,O_{3}\overset{\begin{array}{c} h\,\nu \end{array}}{⟶}C_{17}\,H_{18}\,N_{3}\,O_{3}+F^{-}$$ (10)

Tetracycline, ciprofloxacin, sulfadiazine and sulfamethoxazole are photosensitive antibiotics that can be degraded by both photolysis and photocatalysis. Photocatalysis showed a maximum degradation percentage of 98–99% in comparison to photolysis (Zambrano et al., 2022). UV photolysis can breakdown beta lactam ring found in cefalexin, amoxicillin, and ampicillin. It can also act on the fluorine substituent found in florfenicol and ofloxacin resulting in the elimination of active ingredient. This study also suggests UV photodecomposition to be a necessary pretreatment for antibiotic containing wastewater (Ding et al., 2020). Although photolysis is a simple process that involves direct light-induced degradation of antibiotics, its limited efficiency has led to the development of heterogeneous photocatalysis where semiconductor catalysts are applied to generate enhanced reactive species for improved degradation performance.

The heterogeneous photocatalysis commences with the catalyst absorbing UV radiation, resulting in the generation of hollow electron pairs (Amarzadeh et al., 2023). The generated electrons around the photocatalyst reduces the adsorbed oxygen, leading to the formation of superoxide and hydroxyl radicals that can oxidize the pollutants in the liquid medium (Calvete et al., 2019). Reactions (11)–(14) explains the excitation of photocatalyst, electron generation and conduction into free radicals (Sayadi et al., 2019). Reactions (13) and (14) shows a general reaction where electron-hole pairs react with water to form hydroxyl species which are primary oxidizing species (Saha and Varanasi, 2024). Hydroxyl radicals targets the macrolide ring of azithromycin to form hydroxylated azithromycin which further cleaves the ring to form desosamine-cleaved azithromycin and dihydroxy azithromycin (Bai et al., 2022). Oxidation of amino sugar desosamine leads to the formation of azithromycin amide derivative and an aldehyde derivative described in reaction (15)–(17) (Satulu et al., 2024) and followed by complete mineralization of amide derivative into carbon dioxide, water and inorganic nitrogen containing products like nitrate and ammonium ions (Zouli, 2024).


$$T\,i\,O_{2}+\begin{array}{c} h\,\nu \end{array}\to T\,i\,O_{2}\,\left(e^{-}\right)+T\,i\,O_{2}\,\left(h^{+}\right)$$ (11)

$$T\,i\,O_{2}\,\left(e^{-}\right)+O_{2}\to T\,i\,O_{2}+O_{2}^{-}$$ (12)

$$T\,i\,O_{2}\,\left(h^{+}\right)+H_{2}\,\begin{array}{c} O \end{array}\to T\,i\,O_{2}+\begin{array}{c} O\,H \end{array}+H^{+}$$ (13)

$$h^{+}+O\,H^{-}\to \begin{array}{c} . \end{array}\,\begin{array}{c} O\,H \end{array}$$ (14)

$$C_{38}\,H_{72}\,N_{2}\,O_{12}+\begin{array}{c} . \end{array}\,\begin{array}{c} O\,H \end{array}\to C_{38}\,H_{71}\,N_{2}\,O_{13}+H^{+}$$ (15)

$$C_{38}\,H_{71}\,N_{2}\,O_{13}+\begin{array}{c} . \end{array}\,\begin{array}{c} O\,H \end{array}\to C_{20}\,H_{38}\,N_{2}\,O_{5}+C_{18}\,H_{34}\,O_{9}$$ (16)

$$C_{20}\,H_{38}\,N_{2}\,O_{5}+\begin{array}{c} . \end{array}\,\begin{array}{c} O\,H \end{array}\to C_{10}\,H_{20}\,N_{2}\,O_{2}+C_{10}\,H_{19}\,O_{4}$$ (17)

Spherical metal oxides such as hollow, porous shell, yolk shell and nanoflowers are catalysts in the heterogeneous photocatalysis to enhance the degradation potential of antibiotics like ciprofloxacin, chloramphenicol, ofloxacin, sulphonamides and metronidazole as these oxides have more surface area, more absorption of light and thereby, results in release of oxygen species (Zhu et al., 2024).

Heterogenous photocatalysis based on TiO_2_ exhibited high removal of antibiotics including sulfamethoxazole, amoxicillin, oxolinic acid, tetracycline, erythromycin and spiramycin. This technique can also reduce the total oxygen content and chemical oxygen demand in an aquatic system (Kanakaraju et al., 2019; Nguyen et al., 2020). Another study reported the removal of penicillins, cephalosporins, quinolones, and sulfonamides from distilled water by photocatalysis using the catalyst titanium dioxide P-25. Oxacillin (93%) was shown to have the highest rate of antibiotic elimination, followed by cephradine (91%) and ciprofloxacin (91%), nalidixic acid (86%), ampicillin (75%), sulfacetamide (72%), cephadroxil (58%) and sulfamethoxazole (56%) (Estrada-Flórez et al., 2020).

##### 5.2.2.2 Degradation of antibiotics through ozonation

Ozone, also known as trioxygen, is a strong oxidant due to its ability to react with both organic (bacteria, fungi, viruses, or compounds synthesized from natural and synthetic resources) and inorganic (metals or compounds derived from metal-based elements) compounds that oxidize and eliminate contaminants in water (Malik et al., 2020; Pak et al., 2016; Su et al., 2022). During ozonation of tetracycline, reaction (18) shows that ozone targets the double bond and cleave the ring structure present in tetracycline antibiotic to form tetracycline epoxide and H_2_O_2_ with the release of hydroxyl radicals (Khan et al., 2010) and reaction (19) describes that the degradation is enhanced when epoxide undergoes hydrolysis to form dihydroxy tetracycline by hydroxyl radical induced degradation (Wang et al., 2011). Reaction (20) shows the formation of aromatic tetracycline derivative upon further ozonation of dihydroxy tetracycline. Further nitrogen groups are oxidized into nitrate ions and ammonium ions along with other common antibiotic degradation intermediates like carbon dioxide and water under prolonged ozonation (Hopkins and Blaney, 2014).


$$C_{22}\,H_{24}\,N_{2}\,O_{8}+O_{3}\to C_{22}\,H_{22}\,N_{2}\,O_{9}+H_{2}\,O_{2}$$ (18)

$$C_{22}\,H_{22}\,N_{2}\,O_{9}+\begin{array}{c} . \end{array}\,\begin{array}{c} O\,H \end{array}\to C_{21}\,H_{20}\,N_{2}\,O_{6}+C\,O_{2}+H_{2}\,\begin{array}{c} O \end{array}$$ (19)

$$C_{21}\,H_{20}\,N_{2}\,O_{6}+3\,O_{3}\to C_{16}\,H_{14}\,N_{2}\,O_{2}+5\,C\,O_{2}+3\,H_{2}\,\begin{array}{c} O \end{array}$$ (20)

A study on the “Removal of antibiotic by ozonation” was compiled from various countries across the globe, which found that antibiotics such as ampicillin, azithromycin, clarithromycin, erythromycin, ofloxacin, sulfamethoxazole, tetracycline, and trimethoprim had a degradation rate of more than 84% (Iakovides et al., 2019). Antibiotic resistance genes, such as sul1, tet G, tetA, tetM, tetO, tetQ, tetW, sulI, and sulII, have been eliminated with 90–99% efficiency by ozonation (Alexander et al., 2016; Zhuang et al., 2015) along with significant reduction in the presence of *Escherichia coli*, *Enterococcus faecium*, *Enterococcus faecalis*, and *Estafilococos* sp. (Stange et al., 2019; Zapata-Zúñiga et al., 2022).

**FIGURE 4:**
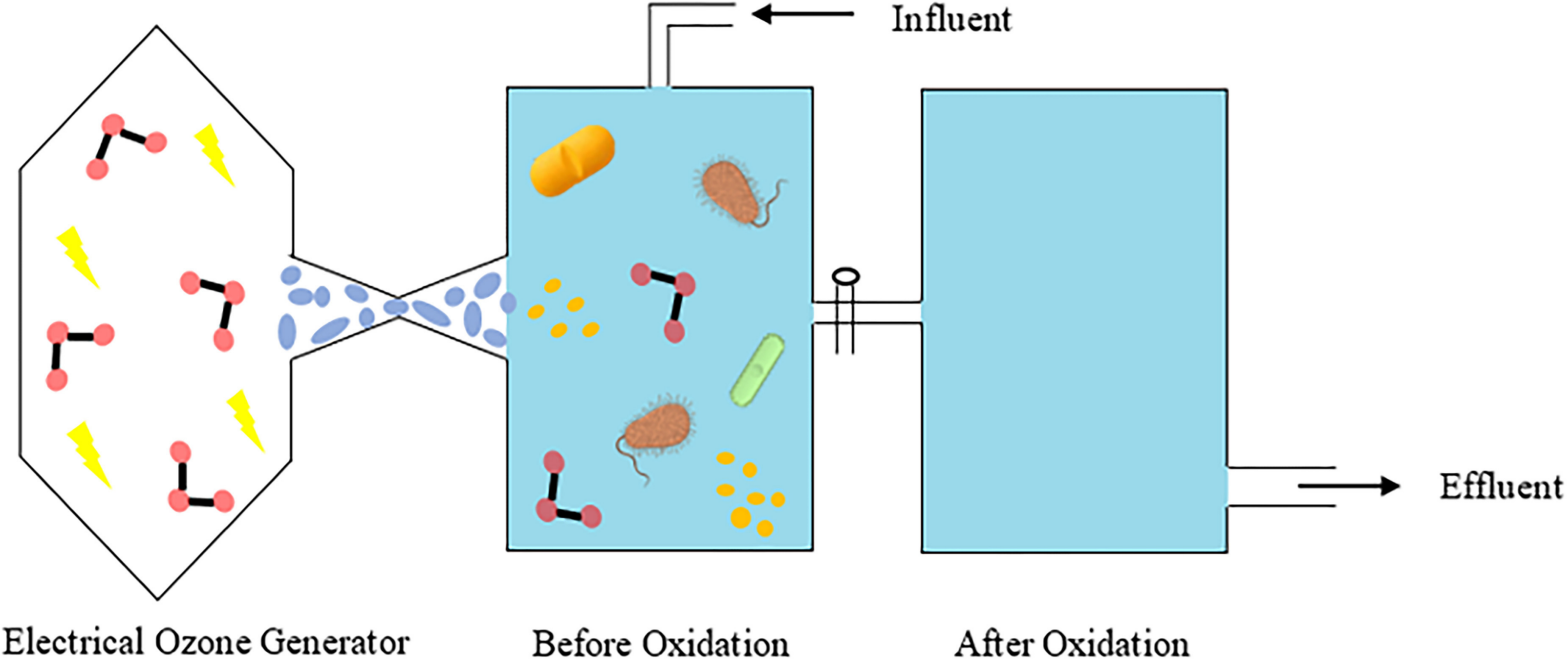
Electrical ozone generator-assisted ozonation for antibiotic and microbial removal in wastewater.

Ecotoxicity assessment for the degraded products of ampicillin, doxycycline, tylosin, and sulfathiazole upon ozonation reported lower mineralization and hence combination with other biodegradation technologies are essential to increase the degradation efficiency. Hence, ozonation can also be used as a pretreatment process to disintegrate the active ingredient so as to prevent the resistance mechanism that can occur during biodegradation approach (Adamek and Baran, 2024; Figure 4).

##### 5.2.2.3 Fenton or Fenton-like processes for the degradation of antibiotic residues

The combination of H_2_O_2_ with Fe^2+^ or Fe^3+^ ions, is called the Fenton or Fenton-like process, whereas when H_2_O_2_ is combined with UV and Fe^2+^ or Fe^3+^ ions, it is called the Photo-Fenton process (Cuerda-correa et al., 2019; Gou et al., 2021). Reaction (21) explains the combined use of Fenton’s reagent and H_2_O_2_ to remove antibiotics upon generation of hydroxyl radicals and ferric ions, whereas, reaction (22) indicate a pathway used to form ferrous ions when ferric ions react with H_2_O_2_ (Cuerda-correa et al., 2019). Hydroxyl radicals generated from reactions (21) and (22) targets the aromatic ring present in sulfamethoxazole and forms hydroxylated sulfamethoxazole, followed by the cleavage of isoxazole ring resulting in the production of 3-amino-5-methylisoxazole and sulphonamide bond is cleaved to produce sulfanilic acid, the processes are explained in reactions (23)–(25) (Borowska et al., 2015; Wang and Wang, 2017). Further oxidation of sulfanilic acid leads to the formation of p-benzoquinone and butanenitrile shown in reaction (26) and (27) (Du et al., 2018; Ahmed et al., 2022).


$$F\,e^{2+}+H_{2}\,O_{2}\to F\,e^{3+}+\begin{array}{c} O\,H \end{array}+O\,H^{-}$$ (21)

$$F\,e^{3+}+H_{2}\,O_{2}\to F\,e^{2+}+H^{+}+H\,O_{2}$$ (22)

$$C_{10}\,H_{11}\,N_{3}\,O_{3}\,\begin{array}{c} S \end{array}+\begin{array}{c} . \end{array}\,\begin{array}{c} O\,H \end{array}\to C_{10}\,H_{11}\,N_{3}\,O_{4}\,\begin{array}{c} S \end{array}+H^{+}$$ (23)

$$C_{10}\,H_{11}\,N_{3}\,O_{4}\,\begin{array}{c} S \end{array}+\begin{array}{c} . \end{array}\,\begin{array}{c} O\,H \end{array}\to C_{4}\,H_{3}\,N_{2}\,\begin{array}{c} O \end{array}+C_{6}\,H_{7}\,N\,O_{3}\,\begin{array}{c} S \end{array}+H_{2}\,\begin{array}{c} O \end{array}$$ (24)

$$C_{6}\,H_{7}\,N\,O_{3}\,\begin{array}{c} S \end{array}+\begin{array}{c} 2. \end{array}\,\begin{array}{c} O\,H \end{array}\to C_{6}\,H_{7}\,N\,O_{3}+S\,O_{2}+H_{2}$$ (25)

$$C_{6}\,H_{7}\,N\,O_{3}+\begin{array}{c} 3. \end{array}\,\begin{array}{c} O\,H \end{array}\to C_{6}\,H_{4}\,O_{2}+2\,H_{2}\,\begin{array}{c} O \end{array}+N\,O_{2}+H_{2}$$ (26)

$$C_{6}\,H_{7}\,N\,O_{3}+\begin{array}{c} 2. \end{array}\,\begin{array}{c} O\,H \end{array}\to C_{4}\,H_{7}\,N+2\,C\,O_{2}+H_{2}\,\begin{array}{c} O \end{array}$$ (27)

Fenton-like process is more appropriate than Fenton process for ciprofloxacin, amoxicillin and erythromycin elimination due to their simpler and easier catalyst recycling, broader application ranges, greater decrease in economic expenses and improved cycle times. Heterogenous Fenton reaction also substantially increases the degradation potential of antibiotics due to its effective catalyst recovery and reusability (Jiang et al., 2022). In a study, antibiotic resistant bacteria and resistance genes, and sulfamethoxazole was eliminated using a modified photo-Fenton technique that uses ethylenediamine-N, N-disuccinic acid (Ahmed et al., 2021).

**FIGURE 5:**
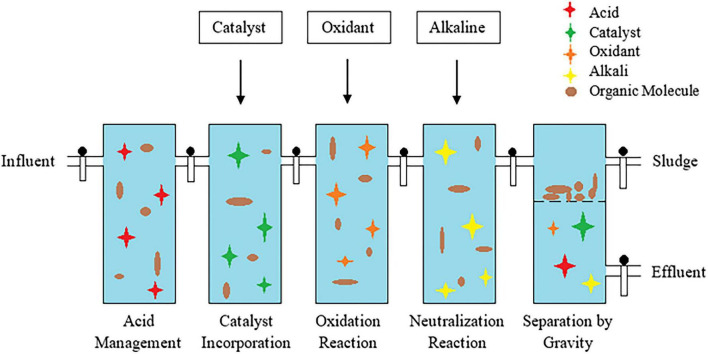
Schematic representation of the multi-stage fenton treatment process for antibiotic degradation.

An example of ciprofloxacin degradation by heterogenous Fenton include a process involving two catalysts for enhanced degradation potential Red mud (RM) is an iron based catalyst and Prussian blue analog (PBA) is an active site for rich iron based metal organic framework. When these two catalysts are dopped with nickel (RM-Ni PBA) their degradation efficiency was increased by 16.63, 1.78, and 1.81 times as compared to RM, RM-PBA and Ni PBA (Liu et al., 2024). Application of UV technology can be restricted to waters consisting of chemicals that are photosensitive or with low COD levels. Therefore, UV/H_2_O_2_ technology is developed, to treat sewage effluents that has a high concentration of organic chemical (Bello et al., 2019; Figure 5). This process is considered as a promising effective approach that can be combined with other technologies for a faster mineralization of wide range of antibiotics.

##### 5.2.2.4 Antibiotic degradation by sonochemical and electrochemical oxidation method

Ultrasounds are sound waves with frequencies above 20 kHz (greater than threshold of human hearing) and up to around 1 GHz (Cárdenas Sierra et al., 2021). When these waves are used in the range of 20 and 100 kHz, they can induce cavitation, leading to the formation of reactive species and the disintegration of contaminants through specific chemical reactions (Estrada-Flórez et al., 2020; Montoya-Rodríguez et al., 2020). During the sonochemical oxidation, cavitation occurs which leads to formation of microbubbles or cavities are formed and implode within the liquid by release of energy (Zhang et al., 2024). Simultaneously, the occurence of cavitation also triggers sonolysis of water, releasing hydrogen peroxide and generate hydroxyl radicals which is shown in reactions (28) and (29) (Cuerda-correa et al., 2019). Sonochemical oxidation method can also be used in combination with Fenton and hydrogen peroxide to increase the degradation rate. Reactions (30) and (31) explains the hydroxylation of metronidazole by hydroxyl species to form hydroxylated metronidazole (Yazdanpanah et al., 2023; Liu et al., 2021), ring cleavage which leads to the formation of acetyl-imidazole derivative and simultaneously, oxidation of hydroxylated metronidazole produces vinyl nitroso fragment (Sun et al., 2023). Followed by mineralization of imidazole derivative leading to the production of carbon dioxide, water and nitrogen containing inorganic ions (Su et al., 2022).


$$2\,H_{2}\,\begin{array}{c} O \end{array}+u\,l\,t\,r\,a\,s\,o\,u\,n\,d\,s\to H_{2}+H_{2}\,O_{2}$$ (28)

$$H_{2}\,O_{2}+u\,l\,t\,r\,a\,s\,o\,u\,n\,d\,s\to 2\,\begin{array}{c} .OH \end{array}\,$$ (29)

$$C_{6}\,H_{9}\,N_{3}\,O_{3}+\begin{array}{c} . \end{array}\,\begin{array}{c} O\,H \end{array}\to C_{6}\,H_{8}\,N_{3}\,O_{4}+H_{2}$$ (30)

$$C_{6}\,H_{8}\,N_{3}\,O_{4}+\begin{array}{c} . \end{array}\,\begin{array}{c} O\,H \end{array}\to C_{4}\,H_{7}\,N_{2}\,O_{3}+C_{2}\,H_{2}\,\begin{array}{c} N \end{array}+C\,O_{2}$$ (31)

A recent study has showed that effect of organic and inorganic substance in sonochemical degradation affects the degradation potential of antibiotics. Cefadroxil in presence of CCl_4_ (organic additive) and Fe^2+^ (inorganic additive) showed higher degradation ability as compared to the absence of these additives. Increase in concentration of CCl_4_ gave better result, whereas higher concentration of Fe^2+^ showed detrimental effects (Guateque-Londoño et al., 2024). Ultrasound waves are employed in the sonochemical oxidation process to eliminate penicillins, cephalosporins, quinolones, and sulfonamides from distilled water. Oxacillin (83%), ampicillin (76%), cephradine (70%), cephadroxil (30%), ciprofloxacin (54%), nalidixic acid (86%), sulfacetamide (47%) and sulfamethoxazole (66%) were the antibiotics that are removed from wastewater. Simultaneously, high frequency ultrasound is also responsible for total eradication of antibacterial activity from the distilled and mineral water (Estrada-Flórez et al., 2020; Serna-Galvis et al., 2016).

Similar to sonochemcial oxidation which utilizes acoustic cavitation to generate reactive radical species, electrochemical oxidation applies anodic reactions to generate oxidizing species. Electrochemical method uses electric current to induce chemical reactions for degradation of pollutants. This degradation can be acquired by direct oxidation of antibiotics at the surface of anode by the release of hydroxyl radicals, in indirect oxidation, degradation is initiated by reactive oxygen species such as hydroxyl radicals, hypochlorite and hydrogen peroxide in the electrolyte solution and reduction occurs at cathode (Choudhary et al., 2021; Ormeno-Cano and Radjenovic, 2022). On the other hand this method has certain drawbacks such as electrode material and maintenance cost, high energy consumption and potential by-product formation. Although these processes effectively remove antibiotics, they are also reported to generate toxic by-products, the need to regenerate or replace adsorbents can be a limitation to its application (Huang et al., 2022). In electrochemical processes, electrodes made of nanomaterials such as graphene and boron-doped diamond provide larger surface area with better conductivity and thereby enabling higher degradation rates.

All the above non-biological technologies involving physical and chemical methods have been used for the removal of antibiotics. However, there are several drawbacks associated with these processes which include inability in complete degradation, production of by-products that are recalcitrant, increase in operational cost and production of sludge. Therefore, identification of a suitable method for the degradation of antibiotics through biological process could pave way to a cost-effective and eco-friendly treatment strategy (Table 4).

**TABLE 4:** Comparative evaluation of physical, chemical, advanced chemical oxidation and biological methods for antibiotic removal using tetracycline as a model compound: advantages, limitations and removal efficiencies.*

Category	Method	Advantages	Limitations	Efficiency (%)	Reference
Physical	Microfiltration	Simple, low energy, no chemical by-product formation and cost-effective	Particle size selectivity, membrane fouling, energy consumption, high initial cost, limited to physical removal, need for pre-treatment and selective removal	<20	(Anis et al., 2019; Shi et al., 2021)
	Nanofiltration	High removal efficiency, charge-based selectivity, no chemical by-product formation and simultaneous removal of other contaminants	Selective removal, energy intensive, membrane fouling, limited to soluble compounds, chemical instability, high capital, high operational costs and scale-up challenges	60–90	(Van der Bruggen et al., 2008; Zylla et al., 2021)
	Ultrafiltration	Remove macromolecules, effective pre-treatment step, no toxic by-product formation and compatibility with hybrid systems	Selective removal, membrane fouling, limited to soluble compounds, operational costs, large treatment facilities, presence of dissolved salts influence membrane selectivity and chemical stability	10–30	(de Ilurdoz et al., 2022; Palacio et al., 2020)
	Reverse osmosis	High removal efficiency, effective for dissolved molecules, broad-spectrum contaminant removal and no chemical by-product formation	Energy intensive, selective removal, membrane fouling, not ideal for high salinity water, chemical sensitivity, high capital cost, waste generation and more suitable for dissolved substances than particulate matter	90–99	(de Ilurdoz et al., 2022; Labella et al., 2021)
Chemical	Chlorination	Widely established technology, cost-effective, rapid result, simultaneous disinfection and easy to operate	Incomplete mineralization, toxic disinfectant by-products, high chlorine demand and sub-lethal chlorination may promote resistance	40–80	(Dou et al., 2022; Zhou et al., 2014)
	Precipitation	Simple operation, low cost, rapid separation and effective for metal-antibiotic complexes	Not degradation but transferred to solid-phase, not applicable for highly soluble antibiotics and risk of antibiotic re-dissolve	10–50	(Abed and Faisal, 2023; Hou et al., 2024)
	Redox reaction	Fast process, suitable for wastewater treatment, and applied for broad variety of antibiotics	Incomplete mineralization, formation of toxic by-products, high oxidant demand, chemical and operational cost and challenges in post treatment neutralization	50–85	(Ahmad et al., 2021; Wang X. et al., 2023)
	Adsorption	High removal efficiency, simple operation, absence of by-product formation, effectively remove traceable concentrations and rapid process	Selective adsorption, limited capacity of adsorbents, competing ions, pH sensitivity, regeneration of adsorbent materials are challenging, relatively slow adsorption rate, adsorbent material cost and biodegradability concerns as no degradation happens	60–95	(Krasucka et al., 2021; Chen et al., 2020)
Advanced chemical oxidation	Photolysis/heterogenous photocatalysis	Solar-driven process, no sludge generation, structural degradation, high oxidative potential, suitable for low-concentration, used for broad variety of antibiotics and can be incorporated in hybrid system	Limited wavelength range, high operational cost, chemical stability, limited penetration depth, replacement of catalyst due to catalyst degradation photolysis efficiency is greatly affected by temperature and require long treatment time	30–60 (photolysis) 70–95 (photo-catalysis)	(Cuerda-correa et al., 2019; Nguyen et al., 2020; Xing et al., 2023)
	Ozonation	Rapid degradation, effective against traceable antibiotic concentration, no sludge generation, applicable on variety of antibiotics and can be incorporated in hybrid technology	Formation of by-products, high operational cost, unstable ozone decomposition, ozone efficiency is temperature dependent, stable antibiotics are resistant to ozonation, pH sensitivity and need for after-treatment to remove residual ozone	70–90	(Merkus et al., 2023; Zhao K. et al., 2024)
	Fenton process	Rapid degradation, significant structural degradation, effective against traceable concentrations, potential for partial to high mineralization and applicable on variety of antibiotics	pH sensitivity, sludge formation, optimized amount of chemicals, high cost of catalytic metals ions, high energy requirement, temperature sensitivity and sludge handling and disposal required	70–95	(Jiang et al., 2022; Gürtekin et al., 2022)
	Sonochemical oxidation method	Highly effective radicals without chemicals, no sludge formation, effective for complex antibiotics, suitable for traceable antibiotic removal and can be combined with other degradation techniques	Selective efficiency, high energy consumption, greatly affected by wastewater composition, high requirement for optimal conditions, significant equipment cost and temperature sensitivity	60–90	(Pereira et al., 2023; Samraj et al., 2024)
	Electrochemical oxidation	Potential for complete mineralization, no external chemical required, easy to operate and suitable for recalcitrant compounds	High energy consumption, electrode fouling and degradation, production of secondary intermediates and sensitivity to wastewater composition	70–95	(Ormeno-Cano and Radjenovic, 2022; Chen et al., 2024)
Biological	Bacterial degradation	Sustainable and environment friendly, biodegradation and biosorption, antibiotic-specific in nature, lower operational cost, suitable for large-scale wastewater treatment, enzymatic capability and can be incorporated with other conventional technologies	Slower degradation rate, sensitive to environmental conditions, greatly affected by wastewater composition, risk of promoting antibiotic resistance genes, require potent bacterial strain and efficiency may decrease at high antibiotic concentration	60–90	(Kato et al., 2009; Yu et al., 2024)
	Fungal degradation	Enzymatic ability to degrade, effective for complex antibiotics, non-specific degradation and suitable for recalcitrant antibiotics	Slower degradation, sensitive to environmental conditions, filamentous mycelial fungal biomass is difficult to handle and limitations scale up	50–85	(Tan et al., 2022; Amangelsin et al., 2023)
	Algal degradation	Combined biodegradation and biosorption, eco-friendly, simultaneous nutrient removal, low chemical requirement and CO_2_ fixation benefit	Light dependant process, antibiotic toxicity might affect algal growth and seasonal variation	30–70	(Li et al., 2022; Chen X. et al., 2023)
	Plant-based degradation	Environmental friendly, cost-effective, multiple removal mechanisms, simultaneous nutrient removal and supports rhizobacterial growth	Slow degradation rate, limited removal efficiency, risk of antibiotic accumulation in plant tissues, sensitive to environmental conditions and risk of resistance development in rhizobacteria	20–60	(Stando et al., 2022; Guo et al., 2020)

*The efficiency values represent reported outcomes from individual experimental studies and may vary depending on antibiotic type, antibiotic solubility, photolytic sensitivity, catalyst, current density, membrane pressure, initial concentration, temperature, pH, ionic strength, presence of organic matter, heavy metals, salinity, COD/BOD levels, contact time, reactor configuration and reactor type.

### 5.3 Mechanisms of antibiotic resistance in bacteria

Bacterial resistance through proteins and enzymes have been a major breakthrough in the degradation of antibiotics. Constant exposure and abuse of antibiotics is often associated with the development of antibiotic resistance. Further, these bacteria can evolve into a strain capable of expressing enzymes that could degrade antibiotics. In simple terms antibiotic resistance, is the ability of bacteria to survive and grow in the presence of antibiotics, normally exposure to these antibiotics kill the organisms (Khan F. et al., 2018).

Primary resistance mechanism which can leads to degradation is enzymatic inactivation of antibiotics. Generally bacteria that can produce enzymes such as beta-lactamase, acetyltransferase, nucleotidyltransferase or oxidoreductase can chemically modify or degrade antibiotics (Kireeva et al., 2021). Efflux pumps are mediated by certain proteins which export antibiotics out of the cell, reducing intracellular drug concentration below inhibitory levels. They can be either specific to a particular class of antibiotic or serve as multi-drug resistant pumps. Major efflux pump families are ATP-Binding Cassette transporters (ABC), Major Facilitator Superfamily (MFS), Resistance-Nodulation-Division (RND), Small Multidrug Resistance (SMR) and Multidrug and Toxic Compound Extrusion (MATE). In Gram-positive bacteria, ABC and MFS function as both importers and exporters, while SMR and MATE transporters primarily act as exporters. In Gram-negative bacteria, porins, ABC and MFS system serve as the major importers whereas RND function as predominant exporter, along with MFS, ABC, SMR and MATE (Blanco et al., 2016). For instance, in *Pseudomonas aeruginosa*, multiple efflux systems known as Mex pumps (RND), mediate resistance against beta lactams, fluoroquinolones, and aminoglycosides. They also maintain lower concentrations of antibiotics by preventing intracellular accumulation (Rocha et al., 2019).

Modification of antibiotic targets is another form of remediation strategy, where proteins synthesized by the bacteria can modify the antibiotic binding sites, making the bacterium resistant to antibiotics (Biondo, 2023). Bacteria alter antibiotic targets by mutating the target genes (gyrA mutation for fluoroquinolone resistance), methylation of ribosomal RNA (mediated by erm genes leading to macrolide resistance) and structural modification of penicillin-binding proteins that reduce beta-lactam binding. For example: *Streptococcus pneumoniae* can produce PspA and PspC, which modifies the surface proteins that can bind to antibiotics and neutralize, blocking their antimicrobial effects (Hasan and Al-Harmoosh, 2020). Target protection proteins bind to the antibiotic target and prevent their antibacterial activity without modifying the target, thereby maintaining its physiological activity even in antibiotic presence. The *Streptomyces* species can synthesize proteins that bind to antibiotics, which enables the bacteria to persist in the presence of antibiotics. Bacteria, like *Escherichia coli*, may produce regulatory proteins MarA, MarB, and MarR, which bind to specific antibiotics like tetracyclines and fluoroquinolones that alter gene expression and confer antibiotic resistance (Li and Nikaido, 2016).

**FIGURE 6:**
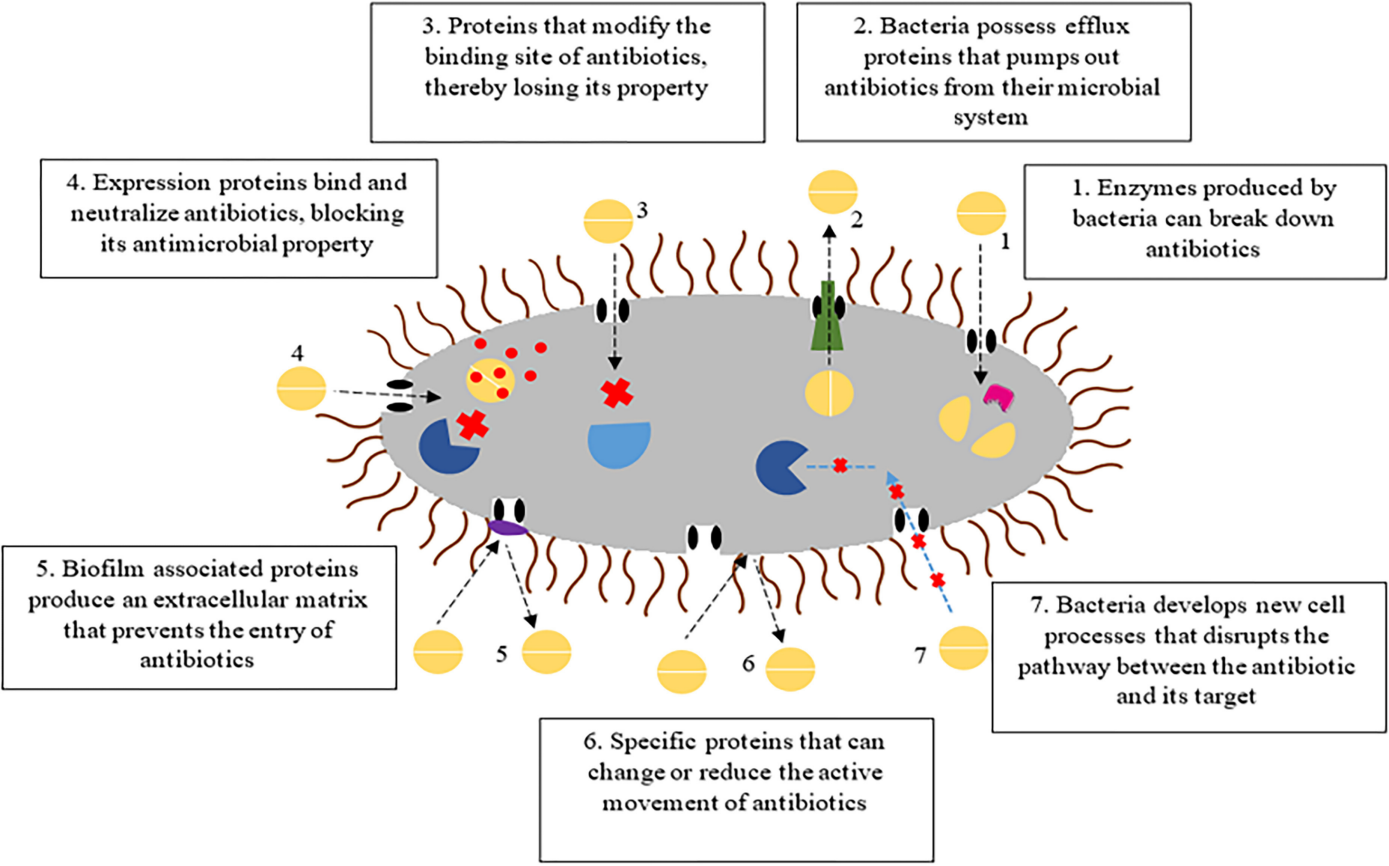
Antibiotic resistance mechanisms involving enzymatic inactivation, biofilm-associated barriers, target site modification, target bind pathway alteration and elimination through efflux pump.

Another mechanism in antibiotic resistance is development of biofilm-associated proteins which aids in the enhanced resistance toward antibiotics (Aleksandrowicz et al., 2023). In biofilm-associated resistance, bacteria produce extracellular polymeric substance (EPS) that restrict antibiotic penetration into the biofilm matrix. Cells within biofilms often exhibit slow growth or dormancy which reduces their susceptibility to antibiotics. Biofilm communities generally possess resistance genes and their expression is enhanced under stress condition. Additionally, existence of cells within the biofilm can enable them to tolerate antibiotic exposure in dormant state and subsequently grow once the environmental condition becomes favorable (Wang N. et al., 2023). A similar mechanism is also observed in *Escherichia coli*, which produces cellulose and curli proteins that provides a protective matrix around the cell as a coating while curli, fibrous proteins, helps in adherence (Sharma et al., 2016; Figure 6). Another resistance strategy involves disruption of antibiotic target pathway. In this mechanism, bacteria blocks the usual target pathway and activate alternative metabolic route to maintain essential functions. Overproduction of antibiotic target enzyme further reduces their antibacterial activity and simultaneously, their stress responses are triggered to repair antibiotic induced damage and enhance their survival (Wilson et al., 2020). These diverse resistance mechanisms enable bacteria to withstand antibiotic exposure by inactivating or degrading antibiotics, modifying or protecting target site and activating alternative pathways for regular functions.

### 5.4 Biological degradation approach to tackle antibiotic

**FIGURE 7:**
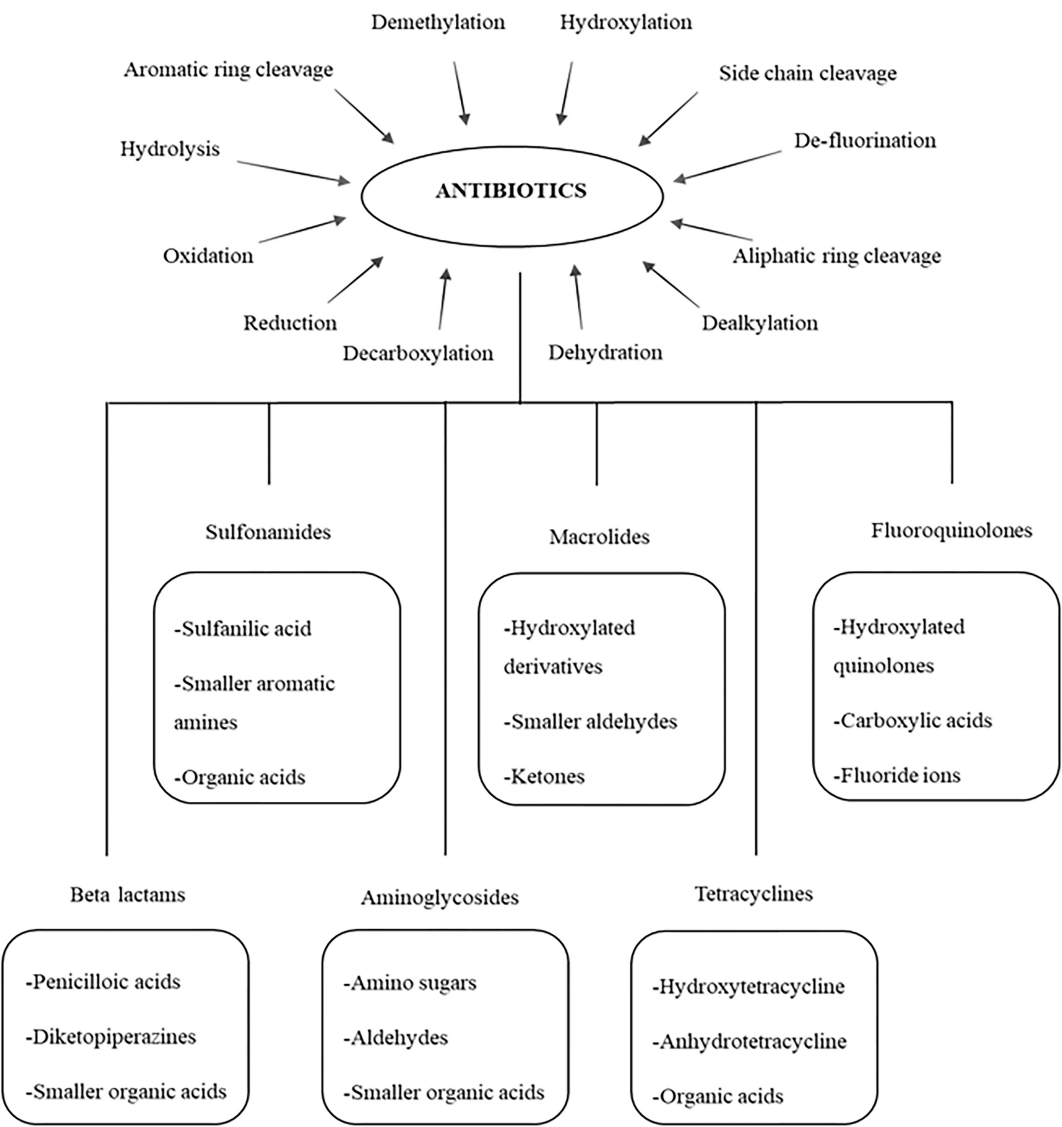
Degradation mechanism of broad spectrum antibiotics and their possible by-product.

**FIGURE 8:**
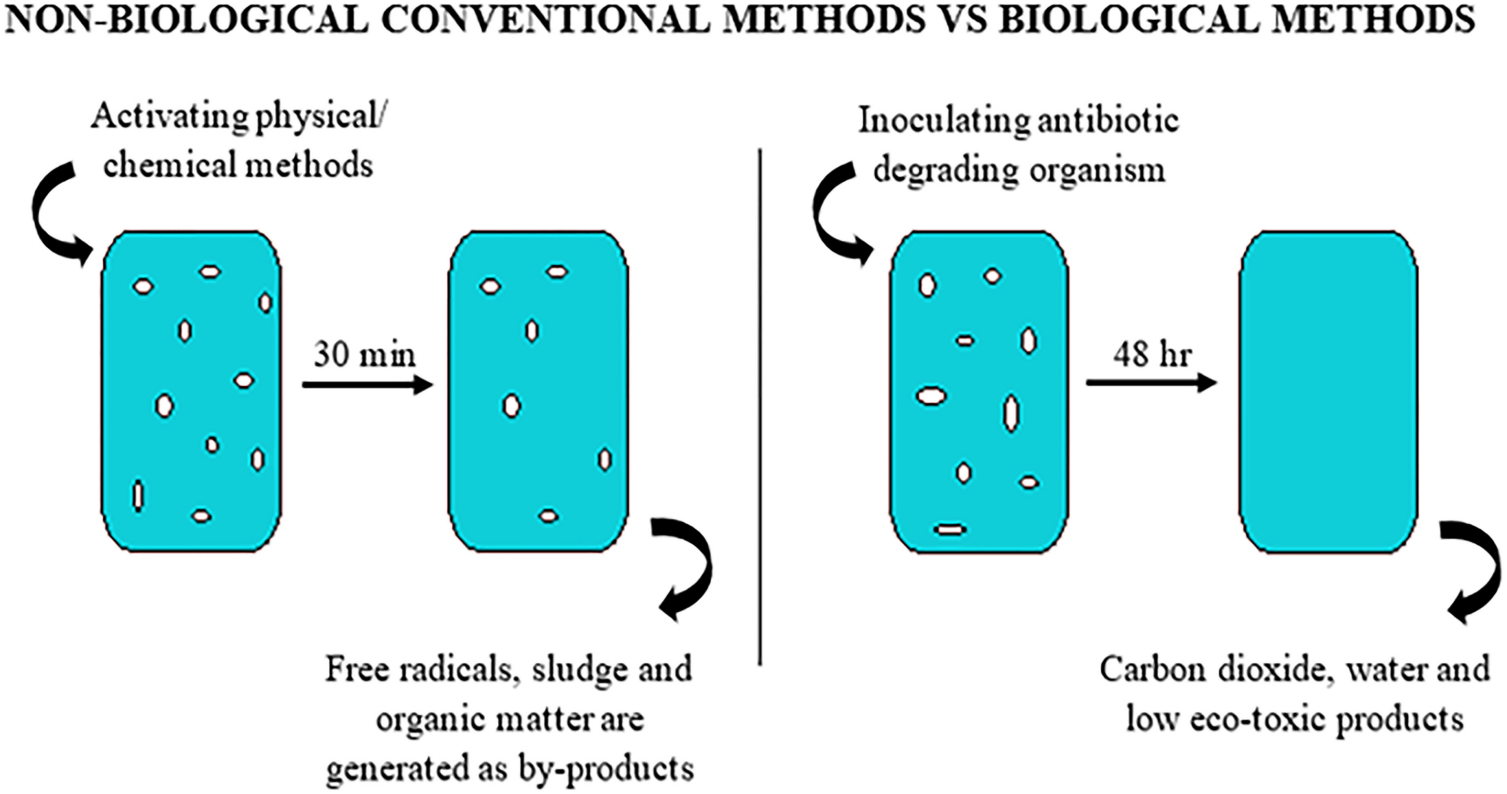
Waste water treatment process by non-biological convention method **(left)** and by biological method **(right)**.

The biological degradation of antibiotics involves the breakdown using various biological agents which includes microbes such as bacteria, fungi, algae and their metabolites (enzymes and proteins) (Chandel et al., 2022). This process produces less toxic by-products with no requirement of chemicals, thereby preventing the generation of chemical sludge. Antibiotic compounds, bacterial resistant genes and other organic matters can be degraded by mechanisms such as demethylation, hydroxylation, side chain cleavage, defluorination, aliphatic ring cleavage, dealkylation, dehydration, decarboxylation, reduction, oxidation, hydrolysis and aromatic ring cleavage leading to production of respective by-products (Kireeva et al., 2021; Navada and Kulal, 2019; Figures 7, 8). Plants are also reported to degrade antibiotics and is often not considered to be effective in the degradation process.

#### 5.4.1 Biodegradation of antibiotics by bacterial enzymes

Bacterial enzymes bind to the ring structure of antibiotic causing the ring to cleave or nullify the effects of antibiotics. Beta-lactam antibiotics can be broken down by a group of bacteria that produce β lactamases (Diene et al., 2019), which hydrolyzes β lactam ring of antibiotics, primarily penicillin and cephalosporin preventing the antibiotic from binding to bacterial penicillin-binding proteins (Kim et al., 2020). Furthermore, bacteria synthesize vital enzymes for the bioremediation of antibiotics which include aminoglycoside modifying enzymes and antibiotic-modifying enzymes (Luthra et al., 2018), macrolide esterases (Nicoloff and Andersson, 2016) and tetracycline inactivating enzyme that chemically modifies and neutralizes their activity (Reis et al., 2020; Table 5).

**TABLE 5:** Classification of antibiotics and associated bacterial genes encoding enzymes responsible for their inactivation and degradation.

Antibiotics class	Effective gene	References
Penicillins	Beta-lactamase genes: bla_TEM_, bla_SHV_, bla_CTX–M_, bla_OXA_, blaAmpC Gene responsible for penicillin-binding protein alterations: mecA Penicillin-specific efflux pump genes: acrAB, mexA, mexB, oprM, adeA, adeB, adeC, norA, mefA	(Ligozzi et al., 1993; Varela et al., 2021; Rice et al., 2001)
Cephalosporins	Beta-lactamase genes: bla_CTX–M_, bla_TEM_, bla_SHV_, blaAmpC, bla_KPC_, bla_NDC_, bla_OXA_ Penicillin-binding protein alteration genes: mecA Porin alteration genes: ompK35, ompK36 Cephalosporin-specific efflux pump genes: acrA, acrB, tolC, mexA, mexB, oprM, adeA, adeB, adeC, norA, acrD, cmeA, cmeB, cmeC	(Varela et al., 2021; Antunes and Fisher, 2014; Bush, 2010; Davidson et al., 2008)
Carbapenems	Beta lactamase genes: bla_GES_, bla_*NDM*_-like Metallo beta lactamase genes: bla_NDM_, bla_IMP_, bla_VIM_ Carbapenem-specific beta lactamase genes: bla_KPC_, bla_OXA–48_ Carbapenem-specific efflux pump: adeA, adeB, adeC, acrA, acrB, tolC, mexA, mexB, oprM, cmeA, cmeB, cmeC, qepA	(Varela et al., 2021; Antunes and Fisher, 2014; Bush, 2010; Davidson et al., 2008; Afzal-Shah et al., 2001; Bush and Jacoby, 2010)
Tetracyclines	Tetracycline-inactivating enzyme genes: tet(X), tet(X1), tet(X2), tet(X3), tet(X3.2), tet(X4), tet(X5), tet(X6), tet(X7), tet(37), tet(47), tet(48), tet(49), tet(50), tet(51), tet(52), tet(53), tet(54), tet(55), tet(56) Tetracycline-specific efflux pump genes: tet(A), tet(B), tet(C), tet(D), tet(E), tet(G), tet(H), tet(J), tet(K), tet(L), tet(V), tet(Y), tet(Z), tet(30), tet(31), tet(33), tet(35), tet(38), tet(39), tet(40), tet(41), tet(42), tet(43), tet(45), tet(57), tet(58), tet(59), tet(62), tetA(P), tetAB(46), tetAB(60), otr(B), otr(C), tcr3 Tetracycline-ribosomal protection genes: tet(M), tet(O), tet(S), tet(Q), tet(T), tet(W), tet(32), tet(36), tet(44), tet(61), otr(A), tetB(P), tet	(Fang et al., 2020; Gasparrini et al., 2020; Markley and Wencewicz, 2018)
Aminoglycosides	Aminoglycoside-ribosomal modifying genes: bla_CTX–M_-_14_, bla_SHV_-_12_, bla_TEM_-1, aac(3)-Ia, aac(3)-II, aac(6’)-Ib-cr, aacA4cr, aacC2, aadA1, aadA2, ant3”9, aphA1, aph(3’)-Ia, aph(3’)-Ib, arr-1, bla_ADC_-_30_, bla_ADC_-_67_, bla_CMY_-_16_, bla_CMY_-_30_, bla_CTX–M_-_3_, bla_CTX–M_-_14_, bla_CTX–M_-_15_, bla_IMP_-_1_, bla_KPC_-_2_, bla_NDM_-_1_, bla_OXA_-_1_, bla_OXA_-_9_, bla_OXA_-_10_, bla_OXA_-_23_, bla_OXA_-_30_, bla_OXA_-_48_, bla_OXA_-_51_, bla_OXA_-_66_, bla_OXA_-_72_, bla_OXA_-_82_, bla_OXA_-_202_, bla_PER_-_1_, bla_SHV_, bla_SHV_-_2_, bla_SHV_-_11_, bla_SHV_-_12_, bla_SHV_-_28_, bla_SHV_-_32_, bla_SHV_-_33_, bla_SHV_-_130_, bla_SHV_-_133_, bla_TEM_-_1_, bla_TEM_-_16_, bla_VEB_-_1_, bla_VIM_-_1_, cmlA1, dfrA12, dfrA14, dfrXII, florR, linF, mel, mph2, qnrA1, qnrB2, qnrS, sul1, sul2, tet(A) Aminoglycoside-specific modifying genes: aac(3)-Ia, aacC1, aac(3)-Ib, aac(3)-Ic, aac(3)-Id, aac(3)-Ie, aacCA5, aac(3)-IIa, aaC3, aacC5, aacC2, aac(3)-Va, aac(3)-IIb, aac(3)-Vb, aac(3)-IIc, aacC2, aac(3)-IIIa, aacC3, aac(3)-IIIb, aac(3)-IIIc, ant(2”)-Ib, aac(3)-IVa, aac(3)VIa, aac(3)-VIIa, aacC7, aac(6’)-Ib, aac(6’)-Ic, aac(6’)-Ie, aac(6’)-If, aac(6’)-Ig, aac(6’)-Ih, aac(6’)-Ii, aac(6’)-Ij, aac(6’)-Ik, aac(6’)-Il, aac(6’)-Im, aac(6’)-Ip, aac(6’)-Iq, aac(6’)-Ir, aac(6’)-Is, aac(6’)-It, aac(6’)-Iu, aac(6’)-Iw	(Bouzidi et al., 2011; Sung et al., 2011; Granier et al., 2011; Paquin et al., 2015)
Macrolides	Macrolide-specific ribosome modifying genes: ermB, ermC, ermF Macrolide modifying genes: mphA, vatA, vatB Macrolide specific efflux pump genes: mefA, mefE, msrA, msrB	(Varela et al., 2021; Andersen et al., 2015; Golkar et al., 2018)
Fluoroquinolones	Mutation occurring genes in account of fluoroquinolones: gyrA, parC Quinolone specific resistance genes: qnrA, qnrB, qnrS Other modifying genes: aac(6’)-Ib-cr Fluoroquinolone specific efflux pump genes: acrA, acrB, tolC, norA, qepA, mexA, mexB, oprM, mexX, mexY, adeA, adeB, adeC	(Varela et al., 2021; Paquin et al., 2015; Andersen et al., 2015)
Sulfonamides	Sulfonamide-inactivating enzyme genes: sul1, sul2, sul3, dfrA1, dfrA5, dfrA7, sulI, sulII Mutation occurring gene in account of sulfonamides: folP Sulfonamide specific efflux pump genes: SmeE, QacA, QacB	(Lunara et al., 2021; de Oliveira-Tintino et al., 2023)
Trimethoprim	Trimethoprim-inactivating enzyme genes: dfrA1, dfrA5, dfrA7, dhfr Other resistant genes: qnrA, qnrB, qnrS Dhfr gene cassettes against trimethoprim: dfrA1, dfrA5, dfrA7 Dfr genes that carry trimethoprim resistant genes: dfrA1, dfrA5, dfrA7, dfrA12, dfrA14, dfrA17 Multidrug resistant efflux pump genes: acrA, acrB, tolC, SmeE, QepA	(Adrian and Klugman, 1997; Ambrose and Hall, 2021; Manna et al., 2021)
Glycopeptides (particularly vancomycin and teicoplanin)	Glycopeptide specific modifying genes: vanA, vanB, vanC, vanD, vanE, vanG, vanH, vanL, vanM, vanN, vanO, vanW, vanX, vanY, vanZ Teicoplanin resistant genes: tei genes Glycopeptide resistant operon located genes: vraS, vraR, vraG	(Varela et al., 2021; Arthur and Quintiliani, 2001; Dougherty and Pucci, 2014)
Oxazolidinones	Oxazolidinone-specific ribosomal modifying genes: cfr, optrA Oxazolidinone resistant genes: poxtA, cfrB	(Varela et al., 2021)

The TEM-1 and TEM-2 lactamases that *Escherichia coli* produces can cleave the beta-lactam ring, imparting resistance toward antibiotics, preventing the interaction of antibiotic with penicillin-binding protein, thus preventing cell wall damage (Cumming et al., 2022; Khalifa et al., 2021) Kanamycin-modifying enzymes including aminoglycoside acetyltransferase, aminoglycoside phosphotransferases, and aminoglycoside nucleotidyltransferases can be produced by *Escherichia coli*, *Enterococcus faecalis*, and *Pseudomonas aeruginosa* which modifies kanamycin through acetylation, phosphorylation or adenylation of its hydroxyl or amino groups, thereby preventing the binding of antibiotic to its ribosomal target (Biondo, 2023; Bodendoerfer et al., 2020; Pang et al., 2019).

ErmA, ErmB, and ErmC are a few commonly found Erm enzymes that work against macrolide antibiotics by methylating 23S rRNA component of 50S ribosomal subunit, which alters the binding site and prevents protein synthesis (Biondo, 2023). ErmA is frequently associated with *Enterococcus faecalis*, whereas ErmB and ErmC are observed in *Staphylococcus aureus* (Biondo, 2023; Miklasińska-Majdanik, 2021). Beta lactamase and Extended Spectrum Beta Lactamases (ESBLs) produced by *Rhizobium* and *Klebisella*, respectively, can degrade 99% of cephalexin as they neutralize the antibiotic by inhibiting the binding of penicillin-binding proteins (Philippon et al., 2016). Degradation study of sulfadiazine by *Brevibacterium epidermidis* and *Castellaniella denitrificans* in three different media showed the effect of carbon sources such as glucose and humic acid in diluted R2A medium on the degradation efficiency. These co-metabolites were reported to stimulate bacterial growth and enzymatic activity which accelerated the degradation of sulfadiazine (Deng et al., 2018). The co-cultivation of bacteria showed maximum degradation in the presence of glucose, whereas the humic acid was unfavorable for the degradation of sulfadiazine. It was observed that hydrolase, oxidoreductase and lyase enzymes produced by *B. epidermidis* and *C. denitrificans* broke down the sulfadiazine (Deng et al., 2018).

Glutathione S-transferases (GSTs) are group of enzymes involved in detoxification and biodegradation of antibiotics such as chloramphenicol and sulphonamides. Bacteria such as *Staphylococcus epidermidis* and *Rhodobacter sphaeroides* are reported to synthesize this group which could degrade antibiotics. In another study, *Bifidobacterium thermophilum* was used due to its antibiotic susceptible nature and can be used as a host so as to enhance the degradation ability of GST enzyme conjugated antibiotic (Park and Choung, 2013). *Sphingosinicella* sp. synthesize hydrolytic enzymes that can degrade the peptide or ester bonds present in sulfamethoxazole and tetracycline (Kato et al., 2009). Similarly, *Mycobacterium smegmatis* expresses aminoglycoside 6-phosphotransferase enzyme that have the potential to degrade streptomycin by phosphorylating its hydroxyl groups that inhibits antibiotic binding to 30S ribosomal subunit, leading to prevention of protein synthesis (Luthra et al., 2018).

A study has compared the biofilm-based system and conventional activated sludge found that biofilm-based process achieved higher antibiotic removal in wastewater treatment system. The biofilm community, dominated by the genuses Proteobacteria, Bacteroidetes, Firmicutes and Actinobacteria were developed in moving bed biofilm reactor and biofilm aerated filters. This bacterial cocktail was capable of degrading antibiotics through metabolism and co-metabolism (utilizing carbon and nitrogen sources) into simpler low-molecular weight products. This biofilm matrix enabled diverse microbial species to coexist and cooperate, enhancing the degradation process (Wang N. et al., 2023).

**FIGURE 9:**
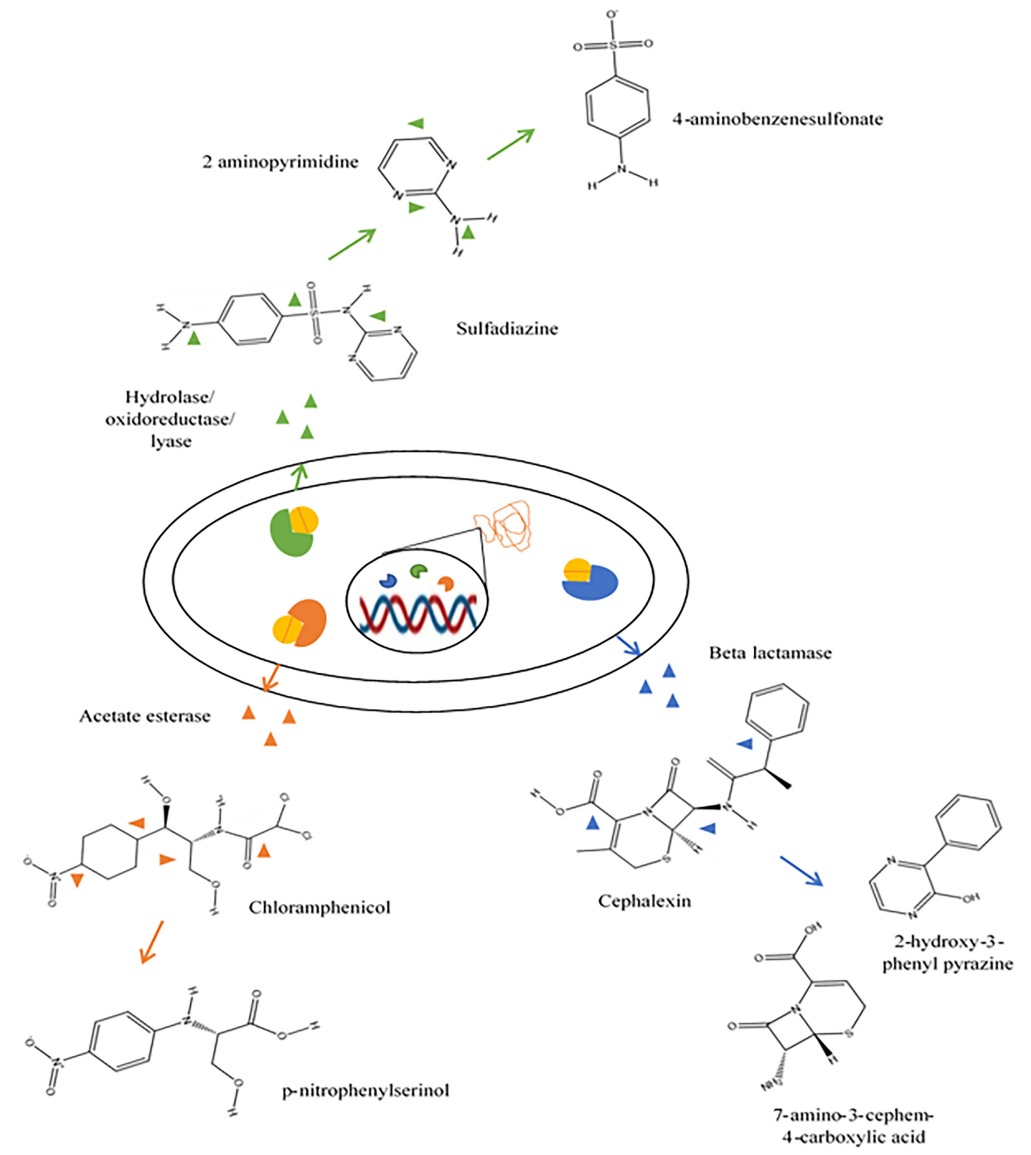
Bacterial enzymes involved in antibiotic degradation and the formation of corresponding metabolites.

The bacterial co-cultivation of *Sphingomonas* sp. and *Caballeronia* sp. showed the degradation of chloramphenicol by a novel enzyme glucose-methanol-choline-oxidoreductase that broke down the chloramphenicol through nitroaromatic oxidation (Zhang et al., 2022). *Escherichia coli* possess estDL136 gene that encodes for the enzyme acetate esterase which is responsible for chloramphenicol degradation. This gene also produces amidohydrolase, dehydrogenase, reductase and esterase that catalyses hydrolysis, reduction and deacetylation leading to changes in the amide and nitro functional groups involved in the degradation of chloramphenicol and florfenicol (Tao et al., 2012; Figure 9).

#### 5.4.2 Role of fungi in the biodegradation of antibiotics

Antibiotics do not affect fungal species naturally as fungi and bacteria have different cell structures and compositions and hence unlike bacteria, fungi do not possess enzymes specific to antibiotics. However, fungi can be engineered with specific bacterial genes for the inactivation of antibiotics and was preferred, as they could thrive in extremely harsh conditions and rejuvenate from its spore form during favorable conditions (Arun et al., 2023). Overall antibiotic elimination efficiency is improved more than 60%, when *Aspergillus niger* was inserted with the bla gene, which makes them capable of producing beta lactamase enzymes that cleaved beta-lactam rings of cefamezin showing 80.45% degradation and 100% degradation was achieved against amoxicillin and ampicillin (Ji et al., 2021). Fungi can also possess efflux pumps, antibiotic-modifying enzymes, antibiotic-modifying proteins, and biofilm-associated proteins, similar to bacteria (Sekyere and Asante, 2018).

Apart from enzymes that specifically inactivate antibiotics, there are other enzymes that cleave the bonds in the antibiotic structure, making them lose their antimicrobial effects. *Trichoderma asperellum* showed 81% degradation against ciprofloxacin, while *Trichoderma harzanium* and *T. asperellum* showed 60% degradation toward ofloxacin. This biotransformation was attained by enzymatic reactions like conjugation, hydroxylation and oxidation/reduction reactions, as these fungal species lack the ability to synthesize extracellular specific enzymes (Manasfi et al., 2020).

Another study that indicated the importance of essential nutritional factors that can also negatively impact the degradation efficiency is the application of *Penicillium oxalicum* RJJ-2 obtained from erythromycin from the contaminated site and it was capable of utilizing erythromycin as the sole carbon source. It had the ability to hydrolyze cladinose and desosamine from erythromycin to 7,12-dihydroxy-6-deoxyerythronolide B, which can further be broken down by an esterase to aldehydes, ketones and short chain fatty acids such as acetate and propionate before undergoing mineralization while releasing carbon dioxide and water (Ren et al., 2021).

*Penicillium funiculosum* NCIM1228 showed significant levels of expression of ABC and MFS family efflux pumps, which resulted in high drug tolerance to nourseothricin (streptothricin), zeocin (glycopeptides), and hygromycin (aminoglycosides) by pumping the antibiotics out of their cell, thereby lowering the intracellular accumulation of antibiotics (Cherazard et al., 2017). In another study, *Cutaneotrichosporon dermatis* M503 was capable of breaking down the methyl and amino groups in tetracycline, doxycycline, and chlorotetracycline (Tan et al., 2022).

To our knowledge, among all the fungal species *Candida* sp. and *Aspergillus nidulans* are known for their antibiotic resistance and degradation (Arastehfar et al., 2020). *Candida* sp. are known for biofilm formation which could shield them from antibiotics (Bhattacharya et al., 2020) and *Aspergillus nidulan* also shows biofilm-associated resistance and produces an enzyme that aids in the acetylation, thereby leading to inactivation of antibiotics. Though resistant mechanisms may vary from species to species and strain to strain, their efficiency in the removal of antibiotics from the natural environment including soil and water bodies has been significant (Liu et al., 2022; Zhang et al., 2021).

#### 5.4.3 Algal-mediated biodegradation of antibiotics

Algae, on the other hand, are photosynthetic organisms and do not have the same enzymatic systems as bacteria and fungi. Algae are directly involved in the elimination of antibiotics from the water through photodegradation, adsorption and absorption, and biotransformation (Li et al., 2022). Antibiotic degradation is also accomplished indirectly through the process of algal-mediated enzyme production. Some of the algae that are reported to show antibiotic degradation includes *Chlorella vulgaris* (Huang et al., 2023), *Microcystis aeruginosa* (Liu et al., 2015), and *Scenedesmus obliquus* (Xiong et al., 2019). Most of the algae follow the mechanism of photodegradation process that breaks down antibiotics and these algae also possess photosynthetic pigments that capture solar energy and initiate photochemical processes that initiates photochemical degradation of antibiotics which also leads to the generation of reactive oxygen species that aid in the removal of antibiotics (Liu et al., 2015).

Both micro and macro algal organisms can physically bind to antibiotics present in water which can be eliminated through adsorption and absorption (Biris-Dorhoi et al., 2016). Several aquatic micro and macro algal species, including *Chlorella* sp., *Spirulina* sp., *Ulva* sp., and *Gracilaria* sp., (Gworek et al., 2021; Yazdi et al., 2018) have reported to adsorb and absorb antibiotics in water, thereby reducing the antibiotic concentration upon binding to their cell surfaces. Some microalgal species are also known to biotransform or modify antibiotics through hydroxylation, dehydrogenation, and conjugation. Researchers have discovered that *Chlorella vulgaris*, *Scenedesmus obliquus*, and several cyanobacterial species can alter the chemical structure of antibiotics like tetracycline, erythromycin, and sulfamethoxazole thereby leading to the degradation of antibiotics (Li et al., 2022; Qi et al., 2022).

Algae can synthesize enzymes such as peroxidase and laccase that can break down antibiotics though they are ahead in the evolutionary origin as compared to bacteria and fungi despite having little similarity in common with bacterial and fungal systems. These enzymes may directly contribute to breakdown or may combine with antibiotic-degrading microorganisms (Chaturvedi et al., 2021a; Mora-Gamboa et al., 2022). When algae engage in photosynthesis, they release organic substances and nutrients into the environment, which are utilized as a source of nutrients by other bacteria, including those that break down antibiotics (Khan M. I. et al., 2018). The diverse community that is linked to algal blooms also possess a range of metabolic capacities (Stamps et al., 2018). Algae and other microbes are observed to have a symbiotic interaction in addition to their physical affinity. They not only have attachment sites for bacteria but also create an environment that supports their growth and activity (Saravanan et al., 2021).

#### 5.4.4 Phytoremediation of antibiotics

Phytoremediation involves application of hyper accumulator plants to uptake, degrade, or stabilize antibiotics from the environment. This approach encompasses various techniques, such as phytoextraction, phytodegradation, rhizofiltration, phytostabilization, phytostimulation, phytotransformation, and phytovolatilization (Zhou et al., 2024). Extensive research has been conducted in the field of phytoextraction, which focuses on the uptake and accumulation of antibiotics by plants (Khan et al., 2023). Key studies have reported the accumulation of oxytetracycline by Indian mustard root tissues (*Brassica juncea*), and vetiver grass (*Chrysopogon zizanioides*) in the extraction of sulfamethazine and chlorotetracycline. The uptake of sulfamethazine and oxytetracycline by spinach (*Spinacia oleracea*) and lettuce (*Lactuca sativa*) (Tang et al., 2021), and the capacity of sunflower (*Helianthus annuus*) to absorb a wide range of antibiotics, particularly tetracycline and sulfamethoxazole, from contaminated soil has also been reported (Conde-Cid et al., 2020).

Plant-based degradation is one of the phytoremediation strategy, where plants metabolize antibiotics into less toxic forms (Khan et al., 2023). Fluoroquinolone antibiotics such as norfloxacin is known to persist in the environment, has been reported to be degraded by Chinese brake fern (*Pteris vittata*). Similarly, ryegrass (*Lolium multiflorum*) was reported in degradation sulfamethoxazole, tetracycline, and ciprofloxacin (Cui et al., 2021). Rhizofiltration involves the trapping of pollutants by the root system of plants, duckweed (*Lemna minor*) has been effective in the phytoremoval of sulfamethoxazole and ciprofloxacin (Khan et al., 2023), while water hyacinth (*Eichhornia crassipes*) was capable of accumulating sulfamethoxazole and tetracycline from water from the rhizosphere region (Kochi et al., 2023).

A similar study using willow tree (*Salix* sp.) showed its ability in immobilizing certain antibiotics tetracycline and sulfamethoxazole through phytostabilization where antibiotics were sequestered in their rhizosphere (Stando et al., 2022). Therefore, reviewing the interaction between plant roots and root-associated microbes may offer valuable insights into the degradation of antibiotics (Khan et al., 2023). Similarly in another study alfalfa plants (*Medicago sativa*) cultivated in sulfamethoxazole contaminated soil exhibited the capacity to degrade antibiotic with the degradation rate being notably higher within the rhizosphere region as compared to the non-rhizosphere region (Conde-Cid et al., 2020). Studies have showed that willow trees (*Salix* sp.) cultivated in soil contaminated with sulfamethoxazole and ciprofloxacin could break down and volatalize antibiotics into the environment. These evidences supports the overall capability of willow tree to be a suitable candidate for the phytoremediation of antibiotics in pollution site (Stando et al., 2022; Khan et al., 2023). However, there are concerns regarding the potential formation of airborne antibiotic residues through the volatilization which could lead to ill-effects on several higher life forms.

Antibiotic degradation by pot culture experiments in controlled environment can be simulated and manipulated using various parameters such as soil type, nutrient additions, moisture levels, and plant selection and screening. In contrast, constructed wetland studies represent real-world scenarios, showing the practical feasibility of phytoremediation (Conde-Cid et al., 2020). Hyperaccumulator plants are capable of removing antibiotics and can be utilized in pot culture studies by enriching the rhizosphere with microbial community in a smaller scale. Whereas, wetland studies are primarily employed for large-scale phytoremediation projects. Duckweed and water hyacinth are the plants that are identified to be suitable for the bioremoval of antibiotics using wetland-based approach (Cui et al., 2021).

Hydroponic system consists of cultivation of plants in the absence of soil (soilless). Studies have shown that plants can co-metabolize antibiotics while utilizing nutrients for their growth and metabolism (Tang et al., 2021). The nutrient solution in these systems can be derived from water polluted with antibiotics and other agrochemicals. Specifically, tomato (*Solanum lycopersicum*), rice (*Oryza sativa*), and spinach (*Spinacia oleracea*) have been shown to have the capacity to remove antibiotics in a mixture consisting of sulfamethazine and tetracycline from the water (Stando et al., 2022). A hybrid system of non-biological and biological approaches can be successfully employed together for faster and effective mitigation of antibiotic pollution in the environment.

## 6 Multi-omics approaches for metabolic pathway elucidation and regulatory mechanism analysis

The identification and quantification of active antibiotic compounds are typically evaluated through several analytical techniques which includes Liquid Chromatography-Mass Spectrometry (LC-MS), Gas Chromatography-Mass Spectrometry (GC-MS), High Performance Liquid Chromatography (HPLC), Ultra Performance Liquid Chromatography (UPLC), Capillary Electrophoresis (CE), Nuclear Magnetic Resonance (NMR) Spectroscopy, Fourier-Transform Infrared (FTIR) Spectroscopy, Enzyme-Linked Immunosorbent Assay (ELISA), and UV-Visible Spectroscopy (Seifrtová et al., 2009).

Metabolomic analysis is considered as a powerful tool that can reveal alterations in metabolite profiles, predict pathway changes, and provide insights into the biological responses of an organism. The metabolites produced during catabolism are directly linked to the ability of the organism in degrading antibiotics via various biomolecules (Aminov, 2022). The typical metabolic pathway and metabolites may differ under stress conditions, such as the presence and concentration of antibiotics, which can trigger specific metabolites as a response to stress. The metabolites generated through degradation can offer significant information about the target of the antibiotic, mode of action, enzymes produced by the organism, and characteristics of metabolic pathways (Xiang et al., 2023).

Certain factors are known to regulate the modification of central metabolic pathways, stress response mechanisms, and specific antibiotic resistance pathways (Xiang et al., 2023). Under stress environment, organisms undergo a comprehensive process that involves DNA damage repair, modifications to central carbon metabolism in glycolysis and Tricarboxylic Acid Cycle (TCA) to maintain the energy production, and mitigation of oxidative stress through the generation of NADPH in the Pentose Phosphate Pathway and anti-oxidative enzymes such as catalase and peroxidase (Dai and Shen, 2022). Heat shock proteins can be expressed in response to the exposure to antibiotic during the repair mechanism of cells affected by the stress response. Bacteria employ various strategies to survive in antibiotic-stressed environments, including modifying antibiotic target sites to prevent binding, enzymatically breaking down antibiotic-binding interactions and the antibiotics themselves, altering lipid membranes or forming biofilm matrices as barriers to antibiotic entry, and utilization of efflux pumps to expel the antibiotics. Additionally, bacteria scavenge alternative nutrient sources like antibiotic components and divert to less affected metabolic pathways to mitigate the effects of antibiotics (Cabral et al., 2018).

Some of the examples involving metabolomic study where the unknown degraded products were assessed by analytical techniques and for the prediction of pathway are as follows: Degradation of cephalexin by *Rhizobium* released into two major degraded metabolites such as 7-amino-3-cephem-4-carboxylic acid and 2-hydroxy-3-phenyl pyrazine (Philippon et al., 2016). In another study, degradation of sulfadiazine by *Brevibacterium epidermidis* and *Castellaniella denitrificans* led to the production of 2-aminopyrimidine, followed by 4-aminobenzenesulfonate (Deng et al., 2018). Similar study revealed the breakdown of streptomycin into streptomycin-6-phosphate as the major metabolite by the action of *Mycobacterium smegmatis* (Luthra et al., 2018) and degradation of chloramphenicol by *Sphingomonas* sp. and *Caballeronia* sp. led to the production of pyruvate, oxaloacetate and 3 oxoadipyl-CoA metabolites (Zhang et al., 2022) and breakdown of chloramphenicol into p-nitrophenylserinol by the enzymatic mechanism of *Escherichia coli* (Tao et al., 2012).

A recent metabolomic study in *Lactobacillus plantarum* examined the impact of ampicillin and doxycycline antibiotics on its metabolic pathways using GC-MS analysis. The study generated a heatmap through R software, revealing major and minor metabolites across different biomolecules including nucleotides, amino acids, carbohydrates, fatty acids, and others (Costa et al., 2016). Notably, the purine nucleotide metabolic pathways were substantially modified, which can significantly affect the synthesis and function of DNA and RNA. Comparing the control (absence of antibiotics) and test (presence of antibiotics) groups, the results showed the emergence of both upregulated and downregulated metabolites. Upregulated metabolites were present in both groups, suggesting they maintain stable concentrations unaffected by antibiotic exposure. Conversely, the downregulated metabolites were highly influenced by the presence of the antibiotics and are present only in the test group (Zhong et al., 2024). Apart from focusing solely on parent compounds, metabolomics offers a comprehensive metabolic overview of microbial or chemical degradation of antibiotics and thereby ensuring the effectiveness and environmental safety of the degradation methods (Dai and Shen, 2022).

Metagenomics is also applied to reveal the wide diversity of microbial taxa present along with the identification of genes responsible for antibiotic resistance and degradation (De Abreu et al., 2021). Similarly, meta-transcriptomics has also been reported to show the active expression of antibiotic resistance and degradation genes (Ota et al., 2024). A field-scale constructed wetland was analyzed to understand the purpose of metagenomics and meta-transcriptomics in antibiotic resistance dynamics. This system reduced approximately 72% of antibiotic resistant genes (ARGs) present in the influent. Metagenomic analysis identified *Proteobacteria* as the primary host of ARGs, while meta-transcriptomics revealed changes in ARGs expression level across the treatment process (Zhao Y. et al., 2024).

Another study using metagenomics identified *Bradyrhizobium*, *Gemmatimonas* and Burkholderiaceae as sulphonamide assimilating bacteria that degraded sulfadiazine and sulfamethoxazole through ipso-hydroxylation mediated by the sadA gene which encoded NADH-dependent monooxygenase enzyme (Chen et al., 2022). Similarly, Fanfoni and others conducted a study that combined metagenomics and untargeted metabolomics to profile microbial communities and associated ARGs in an anaerobic-digestion composting system. Shotgun sequencing and high resolution mass spectrometry identified hundreds of antibiotic compounds including phenicols, sulphonamides, imidazoles and isoxazolines. The study showed that presence of antibiotic and abiotic factors such as physicochemical parameters can influence and shape ARGs profile (Fanfoni et al., 2025).

Another emerging omics approach, metaproteomics, remains less explored, but it has been used to detect enzymes associated with resistance or degradation (Abbondio et al., 2023). A study has examined the effect of sulphonamide on microbial enzyme expression. These enzyme activities reveal the protein level metabolic response under antibiotic stress. Meta-proteomics bridges the gap between genes present in an organism and their protein expression (Kennes-Veiga et al., 2022). These studies have enabled to identify the potential organism for degradation, mechanisms involved, active degradation pathway, genes present and functional expression genes responsible for degradation and correlate microbial activity with the type and concentration of antibiotics.

In addition, several other omics technologies such as phenomics, epigenomics and fluxomics can also be used either in the detection of antibiotics or its resistant genes or their degraded products (Azhar et al., 2023). During the process of antibiotic degradation by microorganisms, phenomics has been reported to identify the functional ability and traits of capable isolates under stress exposure (AlJindan et al., 2024). Epigenomics technology studies the regulatory mechanisms that can lead to the enhancement or suppression of degradation ability (Khan et al., 2022) and fluxomics can be used in quantifying the rate of flow of metabolites that are produced throughout the degradation process in wastewater treatment plant (Mishra and Kumar, 2024). Furthermore, including these advanced omics approaches will be crucial in future studies to achieve more comprehensive and mechanistic understanding of antibiotic degradation in the environment.

## 7 Impact of biodegraded products on ecological health

Biodegradation mimics a natural process with limited usage of external interventions, hazardous chemicals and production of fewer toxic by-products that can be integrated into natural cycles and thereby supporting the ecological health. During the process of biodegradation bacteria, fungi, algae and plants degrade antibiotics, and generate by-products such as carboxylic acid, amines, organic acids, hydrolyzed aromatic compounds, biomass feedstocks and bioenergy precursors (Ahmad et al., 2021). The widespread use of antibiotic classes such as fluoroquinolones, beta-lactams, sulphonamides, tetracyclines and macrolides has led to significant environmental impact from their degraded metabolites. Certain microbial species are capable of producing specific carboxylic acids during the degradation of above mentioned broad spectrum antibiotics (Santhaseelan et al., 2022). *Escherichia coli* produce acetoacetic acid and acetyl-coA derivatives, while *Pseudomonas putida* produces pyruvic acid and succinic acid (Sheikh et al., 2021). Similarly, *Bacillus subtilis* produces lactic and acetic acids, whereas *Pseudomonas aeruginosa* can synthesize gluconic and 2-ketogluconic acids (Gjonbalaj et al., 2020). Additionally, *Enterobacter cloacae* contributes to the formation of formic and acetic acids (Sheikh et al., 2021). These antibiotic-specific carboxylic acids, generated through oxidation can be used in bioplastic production or as substrates for microbial fermentation processes (de Souza and Gupta, 2024). Phenyl acetic acid is an aromatic carboxylic acid produced as a derivative during the degradation of beta-lactams that act as a carbon source for soil microbes, but in excessive quantities it can be toxic to ecological health (García et al., 2024). These carboxylic acids and hydroxylated derivatives are also used as biomass feedstocks to improve the soil quality (de Souza and Gupta, 2024). Certain fungal species such as *Gloeophyllum trabeum*, *Phanerochaete chrysosporium*, *Aspergillus niger* and *Trametes versicolor* play a crucial role in the degradation of beta-lactams, tetracyclines, fluoroquinolones and macrolides. These fungi produce compounds that resemble lignin structures like vanillin (Rehman et al., 2022), syrinaldehyde (Yi et al., 2023) and guaiacol (Black et al., 2021), which can further be incorporated into the soil as organic matter (Amobonye et al., 2023; Ghariani et al., 2025).

The degradation of these broad spectrum antibiotics by diverse microbial and plant species leads to the production of two key organic acids such as acetic and formic acids (Zheng et al., 2022). Acetic acid is obtained as an end product when beta-lactams, sulphonamides and macrolides are degraded by bacteria and plants such as *Pseudomonas aeruginosa* (Glen and Lamont, 2024), *Escherichia coli* (Glen and Lamont, 2021), *Medicago sativa* (Echeverria et al., 2021), *Helianthus annus* (Janusauskaite, 2023) and *Lemna minor* (Glen and Lamont, 2021; Zagórska-Dziok et al., 2020). Formic acid is produced as an end product released by *Pseudomonas putida* (Zhou et al., 2018) and *Eichhornia crassipes* (Ben Bakrim et al., 2022) by oxidizing the side groups of fluoroquinolones. Both these acids are also obtained during the degradation of tetracyclines by *Chlorella vulgaris* and Streptomyces species (Wang et al., 2024). The degradation process involves the mechanisms like ring cleavage, oxidation and hydrolysis, and the resulting organic acids have the potential to support other microbial communities or act as nutrients for plant growth (Zheng et al., 2022). Acetic acid, formic acid, propionic acid, butyric acid and caproic acid are few among the volatile fatty acids that can be biodegraded by *Clostridium butyricum*, *Bacillus subtilis*, *Streptomyces aureofaciens*, and *Trichoderma reesei*. These microorganisms can convert these volatile fatty acids into bioenergy products during the degradation of antibiotics (Dong et al., 2023). Additionally, the degradation of these antibiotics by *Bacillus subtilis*, *Coriandrum sativum*, *Escherichia coli*, *Brassica juncea*, *Fusarium* sp., and *Aspergillus niger* can produce amino alcohols and amines, which can serve as precursors in the synthesis of pharmaceutical products or agrochemicals (Zheng et al., 2022). Therefore, it is evident that the biological degradation of antibiotics does not have harmful effects on the environment due to the biodegradable end products that are released during the metabolism of antibiotics. Hence, biological process can be it is considered as an eco-friendly process of antibiotic degradation.

## 8 Hypothesis

While physical and chemical methods achieve rapid removal, they often result in incomplete mineralization and substantial sludge production. On the other hand, biological methods, despite their slower degradation kinetic rate, can achieve complete mineralization to carbon dioxide and water or other less toxic products which can integrate into natural cycles and thereby could avoid any disturbance that can be caused to the ecological health. Based on the synthesis of current evidence reviewed in this manuscript for the bioremediation of antibiotics using biological and non-biological methods, we propose that a hybrid treatment strategy, incorporating both physical and biological driven remediation, supported by omics-based monitoring can be an effective and sustainable approach for the mitigation of antibiotic residues and associated antimicrobial resistance genes at the application level. A sequential or combined system of these strategies can be a milestone for faster and complete detoxification.

Furthermore, our hypothesis concentrates on application of various omics technologies including metagenomics, meta-transcriptomics, meta-proteomics, metabolomics, fluxomics, epigenomics and phenomics that will serve as reliable tools for pathway prediction, identification of secondary metabolites, monitor the amplification or suppression of genes under stress environment, assessing rate of degradation and traits of capable microorganisms. These technologies can identify and characterize both the parent compound and the intermediates that can provide crucial information about antibiotics, antibiotic degrading genes and antibiotic degrading organisms. Finally, we hypothesize that combining physical, advanced chemical, biological and omics strategies can be effective in the removal of antibiotics and minimize the risk of disseminating antibiotic resistant genes. Testing this hypothesis through pilot scale studies in a controlled laboratory environment and replicating the findings into a real world scenario like wastewater treatment plants will be crucial to confirm its validity and global application.

## 9 Future prospects in bioremediation of antibiotics

The removal of antibiotics from soil or water environment can be expanded from existing literature views to genetic engineering modifications, field studies, and treatment plant strategies. Outlook of the bioremoval of antibiotics involve the use of engineered microorganisms with specialized genes capable of degrading antibiotics through gene transfer methods, as well as the characterization of microorganisms based on their metabolic pathways to understand the underlying degradation mechanisms. Phytoremediation can also be modified by employing genetically modified plants to enhance the uptake of antibiotics. Additionally, microbial enzymes can be processed to develop biocatalysts, as they can produce both specific enzymes like beta-lactamase and non-specific enzymes like hydrolase and carboxylase, which can breakdown and destabilize antibiotics. Degradation of antibiotics can be achieved by multi-systems that involve both biological and non-biological method to achieve efficient and rapid degradation in time, cost efficient and eco-friendly manner. Furthermore, the incorporation of nanomaterials, due to their stability, with microorganisms or enzymes can improve the bioremoval efficiency. Bioaugmentation and biostimulation should be encouraged and supported to facilitate the natural bioremediation process. Lastly, wastewater treatment plants, hospitals, and pharmaceutical industries can implement advanced bioreactors with optimal conditions, including nutrients, temperature, and pH, to effectively degrade antibiotics at the source, before they enter the broader environment.

## 10 Conclusion

Antibiotic residues are emerging contaminants that poses a serious threat to the ecosystem and public health. These contaminants intensify the issue of antibiotic resistance, disruption of microbial communities and contamination of soil and water resources. Consequently, these residues persists their way into all dimensions of life where isolated treatment techniques are becoming insufficient. Physical, chemical, and advanced chemical oxidation methods are known, to remove antibiotics. However, all these methods do not meet their purpose as the concentration of pollutant increases there is an increase in sludge formation, excessive usage of chemicals, and high operational and management costs makes these methods unsuitable for large scale applications. Physical methods concentrate on separation rather than degradation, chemical and advanced chemical methods provide a risk of incomplete mineralization with harmful toxic by-products and secondary pollution, whereas biological approach, though sustainable, are comparatively slower approach. This review emphasizes the value of hybrid treatments involving physical and biological approach to mitigate antibiotics and enhance remediation. The degradation rate and traits of these technical methods can be validated using omics technologies particularly, metabolomics to confirm the reduction in antibiotic detoxification. Such integrated strategies offers a safer, eco-friendly, cost-effective, and regulation-compliant future for remediation of antibiotics.

## References

[B1] Abbondio M. Tanca A. De Diego L. Sau R. Bibbò S. Pes G. M. et al. (2023). Metaproteomic assessment of gut microbial and host functional perturbations in *Helicobacter pylori*-infected patients subjected to an antimicrobial protocol. *Gut Microbes* 15 2291170. 10.1080/19490976.2023.2291170 38063474 PMC10730194

[B2] Abdulrahman S. A. Shnain Z. Y. Ibrahim S. S. Majdi H. S. (2022). Photocatalytic degradation of ciprofloxacin by UV light using N-doped TiO2 in suspension and coated forms. *Catalysts* 12:1663. 10.3390/catal12121663

[B3] Abed M. F. Faisal A. A. (2023). Calcium/iron-layered double hydroxides-sodium alginate for removal of tetracycline antibiotic from aqueous solution. *Alex. Eng. J.* 63 127–142. 10.1016/j.aej.2022.07.055

[B4] Adamek E. Baran W. (2024). Degradation of veterinary antibiotics by the ozonation process: Product identification and ecotoxicity assessment. *J. Hazard. Mater.* 469:134026. 10.1016/j.jhazmat.2024.134026 38493620

[B5] Adrian P. V. Klugman K. P. (1997). Mutations in the dihydrofolate reductase gene of trimethoprim-resistant isolates of *Streptococcus pneumoniae*. *Antimicrob. Agents Chemother.* 41 2406–2413. 10.1128/aac.41.11.2406 9371341 PMC164136

[B6] Afzal-Shah M. Woodford N. Livermore D. M. (2001). Characterization of OXA-25, OXA-26, and OXA-27, molecular class D β-lactamases associated with carbapenem resistance in clinical isolates of *Acinetobacter baumannii*. *Antimicrob. Agents Chemother.* 45 583–588. 10.1128/AAC.45.2.583-588.2001 11158758 PMC90330

[B7] Ahmad F. Zhu D. Sun J. (2021). Environmental fate of tetracycline antibiotics: Degradation pathway mechanisms, challenges, and perspectives. *Environ. Sci. Eur.* 33:64. 10.1186/s12302-021-00505-y

[B8] Ahmed Y. Zhong J. Yuan Z. Guo J. (2021). Simultaneous removal of antibiotic resistant bacteria, antibiotic resistance genes, and micropollutants by a modified photo-Fenton process. *Water Res.* 197:117075. 10.1016/j.watres.2021.117075 33819660

[B9] Ahmed Y. Zhong J. Yuan Z. Guo J. (2022). Roles of reactive oxygen species in antibiotic resistant bacteria inactivation and micropollutant degradation in Fenton and photo-Fenton processes. *J. Hazard. Mater.* 430:128408. 10.1016/j.jhazmat.2022.128408 35150997

[B10] Ainonen S. Tejesvi M. V. Mahmud M. R. Paalanne N. Pokka T. Li W. et al. (2022). Antibiotics at birth and later antibiotic courses: Effects on gut microbiota. *Pediatr. Res.* 91 154–162. 10.1038/s41390-021-01494-7 33824448 PMC8770115

[B11] Akbari M. Z. Xu Y. Lu Z. Peng L. (2021). Review of antibiotics treatment by advance oxidation processes. *Environ. Adv.* 5:100111. 10.1016/j.envadv.2021.100111

[B12] Akhil D. Lakshmi D. Senthil Kumar P. Vo D. V. N. Kartik A. (2021). Occurrence and removal of antibiotics from industrial wastewater. *Environ. Chem. Lett.* 19 1477–1507. 10.1007/s10311-020-01152-0

[B13] Akhremchuk K. V. Skapavets K. Y. Akhremchuk A. E. Kirsanava N. P. Sidarenka A. V. Valentovich L. N. (2022). Gut microbiome of healthy people and patients with hematological malignancies in Belarus. *Microbiol. Indep. Res. J.* 9 18–30. 10.18527/2500-2236-2022-9-1-18-30

[B14] Aleksandrowicz A. Carolak E. Dutkiewicz A. Błachut A. Waszczuk W. Grzymajlo K. (2023). Better together-*Salmonella* biofilm-associated antibiotic resistance. *Gut Microbes* 15:2229937. 10.1080/19490976.2023.2229937 37401756 PMC10321201

[B15] Alexander J. Knopp G. Dötsch A. Wieland A. Schwartz T. (2016). Ozone treatment of conditioned wastewater selects antibiotic resistance genes, opportunistic bacteria, and induce strong population shifts. *Sci. Total Environ.* 559 103–112. 10.1016/j.scitotenv.2016.03.154 27058129

[B16] Alissa R. Maraqa N. Williams P. D. Hipp J. A. Nath S. Torres N. S. et al. (2024). Prevalence of asymptomatic cytomegalovirus (CMV) infection in newborns in northeast Florida. *Front. Epidemiol.* 3:1270374. 10.3389/fepid.2023.1270374 38455916 PMC10910985

[B17] AlJindan R. Mahmoud N. AlEraky D. M. Almandil N. B. AbdulAzeez S. Borgio J. F. (2024). Phenomics and genomic features of *Enterococcus avium* IRMC1622a isolated from a clinical sample of hospitalized patient. *J. Infect. Public Health* 17:102463. 10.1016/j.jiph.2024.05.051 38833914

[B18] Alnajrani M. N. Alsager O. A. (2020). Removal of antibiotics from water by polymer of intrinsic microporosity: Isotherms, kinetics, thermodynamics, and adsorption mechanism. *Sci. Rep.* 10:794. 10.1038/s41598-020-57616-4 31964938 PMC6972944

[B19] Alrefaey K. A. Sallam N. A. ElZayat E. M. Youssef A. F. Fahim I. S. Hosney H. et al. (2025). A comprehensive review of techniques for removal of antibiotics from wastewater. *Environ. Sci. Water Res. Technol.* 18:4909. 10.3390/ijerph18094909 34062980 PMC8125331

[B20] Amangelsin Y. Semenova Y. Dadar M. Aljofan M. Bjørklund G. (2023). The impact of tetracycline pollution on the aquatic environment and removal strategies. *Antibiotics* 12:440. 10.3390/antibiotics12030440 36978308 PMC10044355

[B21] Amarzadeh M. Azqandi M. Nateq K. Ramavandi B. Khan N. A. Nasseh N. (2023). Heterogeneous fenton-like photocatalytic process towards the eradication of tetracycline under UV irradiation: Mechanism elucidation and environmental risk analysis. *Water* 15:2336. 10.3390/w15132336

[B22] Ambrose S. J. Hall R. M. (2021). DfrA trimethoprim resistance genes found in Gram-negative bacteria: Compilation and unambiguous numbering. *J. Antimicrob. Chemother.* 76 2748–2756. 10.1093/jac/dkab212 34180526

[B23] Aminov R. (2022). Metabolomics in antimicrobial drug discovery. *Expert Opin. Drug Discov.* 17 1047–1059. 10.1080/17460441.2022.2113774 35968739

[B24] Aminov R. I. (2010). A brief history of the antibiotic era: Lessons learned and challenges for the future. *Front. Microbiol.* 1:134. 10.3389/fmicb.2010.00134 21687759 PMC3109405

[B25] Amobonye A. Aruwa C. E. Aransiola S. Omame J. Alabi T. D. Lalung J. (2023). The potential of fungi in the bioremediation of pharmaceutically active compounds: A comprehensive review. *Front. Microbiol.* 14:1207792. 10.3389/fmicb.2023.1207792 37502403 PMC10369004

[B26] Andersen J. L. He G. X. Kakarla P. Ranjana K. C. Kumar S. Lakra W. S. et al. (2015). Multidrug efflux pumps from *Enterobacteriaceae*, *Vibrio cholerae* and *Staphylococcus aureus* bacterial food pathogens. *Int. J. Environ. Res. Public Health* 12 1487–1547. 10.3390/ijerph120201487 25635914 PMC4344678

[B27] Anis S. F. Hashaikeh R. Hilal N. (2019). Microfiltration membrane processes: A review of research trends over the past decade. *J. Water Process Eng.* 32:100941. 10.1016/j.jwpe.2019.100941

[B28] Antunes N. T. Fisher J. F. (2014). Acquired class D β-Lactamases. *Antibiotics* 3 398–434. 10.3390/antibiotics3030398 27025753 PMC4790369

[B29] Arastehfar A. Gabaldón T. Garcia-Rubio R. Jenks J. D. Hoenigl M. Salzer H. J. F. et al. (2020). Drug-resistant fungi: An emerging challenge threatening our limited antifungal armamentarium. *Antibiotics* 9:877. 10.3390/antibiotics9120877 33302565 PMC7764418

[B30] Arthur M. Quintiliani R. J. (2001). Regulation of VanA- and VanB-type glycopeptide resistance in enterococci. *Antimicrob. Agents Chemother.* 45 375–381. 10.1128/AAC.45.2.375-381.2001 11158729 PMC90301

[B31] Arun K. B. Madhavan A. Tarafdar A. Sirohi R. Anoopkumar A. N. Kuriakose L. L. et al. (2023). Filamentous fungi for pharmaceutical compounds degradation in the environment: A sustainable approach. *Environ. Technol. Innov.* 31:103182. 10.1016/j.eti.2023.103182

[B32] Azhar F. Busharat M. Chaudhary S. R. A. Waheed Z. Jamil M. N. (2023). Metabolomics in drug discovery: Restoring antibiotic pipeline. *Asian Pac. J. Trop. Biomed.* 13 378–383. 10.4103/2221-1691.385568

[B33] Babuponnusami A. Sinha S. Ashokan H. Paul M. V. Hariharan S. P. Arun J. et al. (2023). Advanced oxidation process (AOP) combined biological process for wastewater treatment: A review on advancements, feasibility and practicability of combined techniques. *Environ. Res.* 237:116944. 10.1016/j.envres.2023.116944 37611785

[B34] Bai X. Chen W. Wang B. Sun T. Wu B. Wang Y. (2022). Photocatalytic degradation of some typical antibiotics: Recent advances and future outlooks. *Int. J. Mol. Sci.* 23:8130. 10.3390/ijms23158130 35897716 PMC9331861

[B35] Barathe P. Kaur K. Reddy S. Shriram V. Kumar V. (2024). Antibiotic pollution and associated antimicrobial resistance in the environment. *J. Hazard. Mater. Lett.* 5:100105. 10.1016/j.hazl.2024.100105

[B36] Bello M. M. Abdul Raman A. A. Asghar A. (2019). A review on approaches for addressing the limitations of Fenton oxidation for recalcitrant wastewater treatment. *Process Saf. Environ. Prot.* 126 119–140. 10.1016/j.psep.2019.03.028

[B37] Ben Bakrim W. Ezzariai A. Karouach F. Sobeh M. Kibret M. Hafidi M. et al. (2022). *Eichhornia crassipes* (Mart.) Solms: A comprehensive review of its chemical composition, traditional use, and value-added products. *Front. Pharmacol.* 13:842511. 10.3389/fphar.2022.842511 35370709 PMC8971373

[B38] Bhatt S. Chatterjee S. (2022). Fluoroquinolone antibiotics: Occurrence, mode of action, resistance, environmental detection, and remediation–A comprehensive review. *Environ. Pollut.* 315:120440. 10.1016/j.envpol.2022.120440 36265724

[B39] Bhattacharya S. Sae-Tia S. Fries B. C. (2020). Candidiasis and mechanisms of antifungal resistance. *Antibiotics* 9:312. 10.3390/antibiotics9060312 32526921 PMC7345657

[B40] Bidoia E. D. Montagnolli R. N. (2021). *Biodegradation, Pollutants and Bioremediation Principles.* Boca Raton, FL: CRC Press, Taylor & Francis Group.

[B41] Biondo C. (2023). Bacterial antibiotic resistance: The most critical pathogens. *Pathogens* 12:116. 10.3390/pathogens12010116 36678464 PMC9863892

[B42] Biris-Dorhoi E. S. Tofana M. Mihãiescu T. Mihãiescu R. Odagiu A. (2016). Applications of microalgae in wastewater treatments: A review. *Proenviron. Promediu* 9 459–463.

[B43] Black J. E. Wagner T. Abbott G. D. (2021). Assessing lignin decomposition and soil organic carbon contents across a tropical savannah-rainforest boundary in Guyana. *Front. For. Glob. Change* 4:629600. 10.3389/ffgc.2021.629600

[B44] Blanco P. Hernando-Amado S. Reales-Calderon J. A. Corona F. Lira F. Alcalde-Rico M. et al. (2016). Bacterial multidrug efflux pumps: Much more than antibiotic resistance determinants. *Microorganisms* 4:14. 10.3390/microorganisms4010014 27681908 PMC5029519

[B45] Blaskovich M. A. Hansford K. A. Butler M. S. Ramu S. Kavanagh A. M. Jarrad A. M. et al. (2022). A lipoglycopeptide antibiotic for Gram-positive biofilm-related infections. *Sci. Transl. Med.* 14:eabj2381. 10.1126/scitranslmed.abj2381 36103517

[B46] Bodendoerfer E. Marchesi M. Imkamp F. Courvalin P. Böttger E. C. Mancini S. (2020). Co-occurrence of aminoglycoside and β-lactam resistance mechanisms in aminoglycoside- non-susceptible *Escherichia coli* isolated in the Zurich area, Switzerland. *Int. J. Antimicrob. Agents* 56:106019. 10.1016/j.ijantimicag.2020.106019 32422315

[B47] Bojarski B. Kot B. Witeska M. (2020). Antibacterials in aquatic environment and their toxicity to fish. *Pharmaceuticals* 13:189. 10.3390/ph13080189 32784912 PMC7464759

[B48] Borowska E. Felis E. Miksch K. (2015). Degradation of sulfamethoxazole using UV and UV/H2O2 processes. *J. Adv. Oxid. Technol.* 18 69–77. 10.1515/jaots-2015-0109

[B49] Bouzidi N. Aoun L. Dekhil M. Granier S. A. Poirel L. Brisabois A. et al. (2011). Co-occurrence of aminoglycoside resistance gene armA in non-Typhi *Salmonella* isolates producing CTX-M-15 in Algeria. *J. Antimicrob. Chemother.* 66 2180–2181. 10.1093/jac/dkr237 21676906

[B50] Bricchi M. Asti A. Pascale A. Andreini F. Arghittu M. Caldiroli D. et al. (2020). Implementation of preventive actions to control carbapenem-resistant *enterobacteriaceae* and mdr gram negatives at a neurological hospital. *J. Microbiol. Infect. Dis.* 10 1–9. 10.5799/jmid.700466

[B51] Brüssow H. (2020). Problems with the concept of gut microbiota dysbiosis. *Microb. Biotechnol.* 13 423–434. 10.1111/1751-7915.13479 31448542 PMC7017827

[B52] Buffie C. G. Jarchum I. Equinda M. Lipuma L. Gobourne A. Viale A. et al. (2012). Profound alterations of intestinal microbiota following a single dose of clindamycin results in sustained susceptibility to Clostridium difficile-induced colitis. *Infect. Immun.* 80 62–73. 10.1128/IAI.05496-11 22006564 PMC3255689

[B53] Bush K. (2010). Bench-to-bedside review: The role of β-lactamases in antibiotic-resistant Gram-negative infections. *Crit. Care* 14:224. 10.1186/cc8892 20594363 PMC2911681

[B54] Bush K. Jacoby G. A. (2010). Updated functional classification of β-lactamases. *Antimicrob. Agents Chemother.* 54 969–976. 10.1128/AAC.01009-09 19995920 PMC2825993

[B55] Cabral D. J. Wurster J. I. Belenky P. (2018). Antibiotic persistence as a metabolic adaptation: Stress, metabolism, the host, and new directions. *Pharmaceuticals* 11:14. 10.3390/ph11010014 29389876 PMC5874710

[B56] Calvete M. J. F. Piccirillo G. Vinagreiro C. S. Pereira M. M. (2019). Hybrid materials for heterogeneous photocatalytic degradation of antibiotics. *Coord. Chem. Rev.* 395 63–85. 10.1016/j.ccr.2019.05.004

[B57] Cárdenas Sierra R. S. Zúñiga-Benítez H. Peñuela G. A. (2021). Elimination of cephalexin and doxycycline under low frequency ultrasound. *Ultrason. Sonochem.* 79:105777. 10.1016/j.ultsonch.2021.105777 34649167 PMC8517921

[B58] Castle S. C. Uyemura K. Fulop T. Makinodan T. (2007). Host resistance and immune responses in advanced age. *Clin. Geriatr. Med.* 23 463–479. 10.1016/j.cger.2007.03.005 17631228 PMC7135540

[B59] Cederberg Å. Denneberg T. Ekberg M. Juhlin I. (1974). Nalidixic acid in urinary tract infections with particular reference to the emergence of resistance. *Scand. J. Infect. Dis.* 6 259–264. 10.3109/inf.1974.6.issue-3.09 4607842

[B60] Cetecioglu Z. Atasoy M. (2018). “Biodegradation and inhibitory effects of antibiotics on biological wastewater treatment systems,” in *Toxicity and Biodegradation Testing: Methods in Pharmacology and Toxicology*, eds Bidoia E. Montagnolli R. (New York, NY: Humana Press), 29–55. 10.1007/978-1-4939-7425-2_2

[B61] Chakraborty A. Amrit R. Dutta P. Osborne W. J. (2025). Unlocking nature’s vault: Endophytes as plant-sourced biological treasures. *Biocatal. Agric. Biotechnol.* 67:103685. 10.1016/j.bcab.2025.103685

[B62] Chandel N. Ahuja V. Gurav R. Kumar V. Tyagi V. K. Pugazhendhi A. et al. (2022). Progress in microalgal mediated bioremediation systems for the removal of antibiotics and pharmaceuticals from wastewater. *Sci. Total Environ.* 825:153895. 10.1016/j.scitotenv.2022.153895 35182616

[B63] Chaturvedi P. Giri B. S. Shukla P. Gupta P. (2021a). Recent advancement in remediation of synthetic organic antibiotics from environmental matrices: Challenges and perspective. *Bioresour. Technol.* 319:124161. 10.1016/j.biortech.2020.124161 33007697

[B64] Chaturvedi P. Shukla P. Giri B. S. Chowdhary P. Chandra R. Gupta P. et al. (2021b). Prevalence and hazardous impact of pharmaceutical and personal care products and antibiotics in environment: A review on emerging contaminants. *Environ. Res.* 194:110664. 10.1016/j.envres.2020.110664 33400949

[B65] Chen J. Yang Y. Ke Y. Chen X. Jiang X. Chen C. et al. (2022). Sulfonamide-metabolizing microorganisms and mechanisms in antibiotic-contaminated wetland sediments revealed by stable isotope probing and metagenomics. *Environ. Int.* 165:107332. 10.1016/j.envint.2022.107332 35687947

[B66] Chen J. Yu X. Li C. Tang X. Sun Y. (2020). Removal of tetracycline via the synergistic effect of biochar adsorption and enhanced activation of persulfate. *Chem. Eng. J.* 382:122916. 10.1016/j.cej.2019.122916

[B67] Chen X. Ke Y. Zhu Y. Xu M. Chen C. Xie S. (2023). Enrichment of tetracycline-degrading bacterial consortia: Microbial community succession and degradation characteristics and mechanism. *J. Hazard. Mater.* 448:130984. 10.1016/j.jhazmat.2023.130984 36860056

[B68] Chen Y. Zuo W. Sun H. Liu Y. Hou J. Xie H. et al. (2024). Electrochemical oxidation of low concentrated tetracycline (TC) in aqueous solution: Operation optimization, degradation mechanisms, and detoxification efficiency. *J. Environ. Chem. Eng.* 12:112604. 10.1016/j.jece.2024.112604

[B69] Chen Z. Ou D. Gu G. Gao S. Li X. Hu C. et al. (2023). Removal of tetracycline from water by catalytic photodegradation combined with the microalga *Scenedesmus obliquus* and the responses of algal photosynthesis and transcription. *J. Environ. Manage.* 326:116693. 10.1016/j.jenvman.2022.116693 36347215

[B70] Cherazard R. Epstein M. Doan T. L. Salim T. Bharti S. Smith M. A. (2017). Antimicrobial resistant *Streptococcus pneumoniae*: Prevalence, mechanisms, and clinical implications. *Am. J. Ther.* 24 e361–e369. 10.1097/MJT.0000000000000551 28430673

[B71] Chopra I. Roberts M. (2001). Tetracycline antibiotics: Mode of action, applications, molecular biology, and epidemiology of bacterial resistance. *Microbiol. Mol. Biol. Rev.* 65 232–260. 10.1128/mmbr.65.2.232-260.2001 11381101 PMC99026

[B72] Choudhary V. Vellingiri K. Thayyil M. I. Philip L. (2021). Removal of antibiotics from aqueous solutions by electrocatalytic degradation. *Environ. Sci. Nano* 8 1133–1176. 10.1039/D0EN01276A

[B73] Conde-Cid M. Núñez-Delgado A. Fernández-Sanjurjo M. J. Álvarez-Rodríguez E. Fernández-Calviño D. Arias-Estévez M. (2020). Tetracycline and sulfonamide antibiotics in soils: Presence, fate and environmental risks. *Processes* 8:1479. 10.3390/pr8111479 32028162

[B74] Costa C. Maraschin M. Rocha M. (2016). An R package for the integrated analysis of metabolomics and spectral data. *Comput. Methods Prog. Biomed.* 129 117–124. 10.1016/j.cmpb.2016.01.008 26853041

[B75] Cristóvão M. B. Tela S. Silva A. F. Oliveira M. Bento-Silva A. Bronze M. R. et al. (2021). Occurrence of antibiotics, antibiotic resistance genes and viral genomes in wastewater effluents and their treatment by a pilot scale nanofiltration unit. *Membranes* 11:9. 10.3390/membranes11010009 33374743 PMC7824572

[B76] Cuerda-correa E. M. Alexandre-franco M. F. Fern C. (2019). Advanced oxidation processes for the removal of antibiotics from water. An overview. *Water* 12:102. 10.3390/w12010102

[B77] Cui E. Cui B. Fan X. Li S. Gao F. (2021). Ryegrass (*Lolium multiflorum* L.) and Indian mustard (*Brassica juncea* L.) intercropping can improve the phytoremediation of antibiotics and antibiotic resistance genes but not heavy metals. *Sci. Total Environ.* 784:147093. 10.1016/j.scitotenv.2021.147093 33895506

[B78] Cumming A. J. Khananisho D. Harris R. Bayer C. N. Nørholm M. H. H. Jamshidi S. et al. (2022). Antibiotic-efficient genetic cassette for the TEM-1 β-lactamase that improves plasmid performance. *ACS Synth. Biol.* 11 241–253. 10.1021/acssynbio.1c00393 34982550 PMC8787818

[B79] Dai X. Shen L. (2022). Advances and trends in omics technology development. *Front. Med.* 9:911861. 10.3389/fmed.2022.911861 35860739 PMC9289742

[B80] Davidson A. L. Dassa E. Orelle C. Chen J. (2008). Structure, function, and evolution of bacterial ATP-binding cassette systems. *Microbiol. Mol. Biol. Rev.* 72 317–364. 10.1128/mmbr.00031-07 18535149 PMC2415747

[B81] De Abreu V. A. Perdigão J. Almeida S. (2021). Metagenomic approaches to analyze antimicrobial resistance: An overview. *Front. Genet.* 11:575592. 10.3389/fgene.2020.575592 33537056 PMC7848172

[B82] de Ilurdoz M. S. Sadhwani J. J. Reboso J. V. (2022). Antibiotic removal processes from water & wastewater for the protection of the aquatic environment-a review. *J. Water Process Eng.* 45:102474. 10.1016/j.jwpe.2021.102474

[B83] de Oliveira-Tintino C. D. M. Tintino S. R. Justino de Araújo A. C. dos Santos Barbosa C. R. Ramos Freitas P. de Araújo Neto J. B. et al. (2023). Efflux Pump (QacA, QacB, and QacC) and β-lactamase inhibitors? An evaluation of 1,8-naphthyridines against *Staphylococcus aureus* strains. *Molecules* 28:1819. 10.3390/molecules28041819 36838807 PMC9961278

[B84] de Souza F. M. Gupta R. K. (2024). Bacteria for bioplastics: Progress, applications, and challenges. *ACS Omega* 9 8666–8686. 10.1021/acsomega.3c07372 38434856 PMC10905720

[B85] Deng Y. Li B. Zhang T. (2018). Bacteria that make a meal of sulfonamide antibiotics: Blind spots and emerging opportunities. *Environ. Sci. Technol.* 52 3854–3868. 10.1021/acs.est.7b06026 29498514

[B86] Derderian S. L. (2007). Alexander Fleming’s miraculous discovery of penicillin. *Rivier Acad. J.* 3 1–5.

[B87] Diene S. M. Pinault L. Keshri V. Armstrong N. Khelaifia S. Chabrière E. et al. (2019). Human metallo-β-lactamase enzymes degrade penicillin. *Sci. Rep.* 9:12173. 10.1038/s41598-019-48723-y 31434986 PMC6704141

[B88] Ding Y. Jiang W. Liang B. Han J. Cheng H. Haider M. R. et al. (2020). UV photolysis as an efficient pretreatment method for antibiotics decomposition and their antibacterial activity elimination. *J. Hazard. Mater.* 392:122321. 10.1016/j.jhazmat.2020.122321 32092653

[B89] Dong K. Wang W. Li M. Zhou X. Huang Y. Zhou G. et al. (2023). Degradation of sulfonamide antibiotics in the rhizosphere of two dominant plants in Huixian karst wetland, Guangxi, China. *Water Reuse* 13 18–32. 10.2166/wrd.2023.062

[B90] Dou Y. Cheng X. Miao M. Wang T. Hao T. Zhang Y. et al. (2022). The impact of chlorination on the tetracycline sorption behavior of microplastics in aqueous solution. *Sci. Total Environ.* 849 157800. 10.1016/j.scitotenv.2022.157800 35934036

[B91] Dougherty T. J. Pucci M. J. (2014). *Antibiotic Discovery and Development.* (New York, NY: Springer), 1–1127. 10.1007/978-1-4614-1400-1

[B92] Dowling H. F. (1973). Comparisons and contrasts between the early arsphenamine and early antibiotic periods. *Bull. Hist. Med.* 47 236–249. 4586944

[B93] Du J. Guo W. Wang H. Yin R. Zheng H. Feng X. et al. (2018). Hydroxyl radical dominated degradation of aquatic sulfamethoxazole by Fe0/bisulfite/O2: Kinetics, mechanisms, and pathways. *Water Res.* 138 323–332. 10.1016/j.watres.2017.12.046 29627708

[B94] Dutta J. Mala A. A. (2020). Removal of antibiotic from the water environment by the adsorption technologies: A review. *Water Sci. Technol.* 82 401–426. 10.2166/wst.2020.335 32960788

[B95] Echeverria A. Larrainzar E. Li W. Watanabe Y. Sato M. Tran C. D. et al. (2021). *Medicago sativa* and *Medicago truncatula* show contrasting root metabolic responses to drought. *Front. Plant Sci.* 12:652143. 10.3389/fpls.2021.652143 33968107 PMC8097159

[B96] Enshaie E. Nigam S. Patel S. Rai V. (2025). Livestock antibiotics use and antimicrobial resistance. *Antibiotics* 14:621. 10.3390/antibiotics14060621 40558211 PMC12189104

[B97] Escobar-Huerfano F. Gómez-Oliván L. M. Luja-Mondragón M. SanJuan-Reyes N. Islas-Flores H. Hernández-Navarro M. D. (2020). Embryotoxic and teratogenic profile of tretracycline at environmentally relevant concentrations on *Cyprinus carpio*. *Chemosphere* 240:124969. 10.1016/j.chemosphere.2019.124969 31726589

[B98] Estrada-Flórez S. E. Serna-Galvis E. A. Torres-Palma R. A. (2020). Photocatalytic vs. sonochemical removal of antibiotics in water: Structure-degradability relationship, mineralization, antimicrobial activity, and matrix effects. *J. Environ. Chem. Eng.* 8:104359. 10.1016/j.jece.2020.104359

[B99] Ezeuko A. S. Ojemaye M. O. Okoh O. O. Okoh A. I. (2021). Potentials of metallic nanoparticles for the removal of antibiotic resistant bacteria and antibiotic resistance genes from wastewater: A critical review. *J. Water Process Eng.* 41:102041. 10.1016/j.jwpe.2021.102041

[B100] Fanfoni E. Bellassi P. Fontana A. Sinisgalli E. Rocchetti G. Piccinini S. et al. (2025). Metagenomics and untargeted metabolomics reveal antibiotic resistance dynamics in an anaerobic digestion–composting system treating organic fraction of municipal solid waste. *Environ. Microbiome* 20:106. 10.1186/s40793-025-00769-4 40813715 PMC12355879

[B101] Fang L. X. Chen C. Cui C. Y. Li X. P. Zhang Y. Liao X. P. et al. (2020). Emerging high-level tigecycline resistance: Novel tetracycline destructases spread via the Mobile Tet(X). *Bioessays* 42:e2000014. 10.1002/bies.202000014 32567703

[B102] Field R. W. She Q. Siddiqui F. A. Fane A. G. (2021). Reverse osmosis and forward osmosis fouling: A comparison. *Discov. Chem. Eng.* 1:6. 10.1007/s43938-021-00006-7

[B103] Gaikowski M. P. Wolf J. C. Schleis S. M. Gingerich W. H. (2003). Safety of oxytetracycline (Terramycin TM-100F) administered in feed to hybrid striped bass, walleyes, and yellow perch. *J. Aquat. Anim. Health* 15 274–286. 10.1577/H03-042.1

[B104] Gandhi P. Ingole R. K. (2023). Arsenicals review: Poison vis-a-vis medicine. *Int. J. Ayurveda Pharma Res.* 11 73–81. 10.47070/ijapr.v11i1.2625

[B105] García A. Aguirre C. Pérez A. Bahamonde S. S. Urtuvia V. Díaz-Barrera A. et al. (2024). Recent trends in the production and recovery of bioplastics using polyhydroxyalkanoates copolymers. *Microorganisms* 12:2135. 10.3390/microorganisms12112135 39597527 PMC11596358

[B106] Garrod L. P. (1957). The erythromycin group of antibiotics. *Br. Med. J.* 2:57. 10.1136/bmj.2.5036.57 13436854 PMC1961747

[B107] Gasparrini A. J. Markley J. L. Kumar H. Wang B. Fang L. Irum S. et al. (2020). Tetracycline-inactivating enzymes from environmental, human commensal, and pathogenic bacteria cause broad-spectrum tetracycline resistance. *Commun. Biol.* 3:241. 10.1038/s42003-020-0966-5 32415166 PMC7229144

[B108] Ghariani B. Zouari-Mechichi H. Alessa A. H. Alqahtani H. Alsaigh A. A. Mechichi T. (2025). Biotransformation of antibiotics by *Coriolopsis gallica*: Degradation of compounds does not always eliminate their toxicity. *Antibiotics* 14:897. 10.3390/antibiotics14090897 41009876 PMC12466409

[B109] Gjonbalaj M. Keith J. W. Do M. H. Hohl T. M. Pamer E. G. Becattini S. (2020). Antibiotic degradation by commensal microbes shields pathogens. *Infect. Immun.* 88 10–1128. 10.1128/iai.00012-20 31964746 PMC7093146

[B110] Glen K. A. Lamont I. L. (2021). β-lactam resistance in *Pseudomonas aeruginosa*: Current status, future prospects. *Pathogens* 10:1638. 10.3390/pathogens10121638 34959593 PMC8706265

[B111] Glen K. A. Lamont I. L. (2024). Characterization of acquired β-lactamases in *Pseudomonas aeruginosa* and quantification of their contributions to resistance. *Microbiol. Spectr.* 12:e00694-24. 10.1128/spectrum.00694-24 39248479 PMC11448201

[B112] Golkar T. Zielinski M. Berghuis A. M. (2018). Look and outlook on enzyme-mediated macrolide resistance. *Front. Microbiol.* 9:1942. 10.3389/fmicb.2018.01942 30177927 PMC6109786

[B113] Gou Y. Chen P. Yang L. Li S. Peng L. Song S. et al. (2021). Degradation of fluoroquinolones in homogeneous and heterogeneous photo-Fenton processes: A review. *Chemosphere* 270:129481. 10.1016/j.chemosphere.2020.129481 33423001

[B114] Granier S. A. Hidalgo L. Millan A. S. Escudero J. A. Gutierrez B. Brisabois A. et al. (2011). ArmA methyltransferase in a monophasic *Salmonella enterica* isolate from food. *Antimicrob. Agents Chemother.* 55 5262–5266. 10.1128/AAC.00308-11 21859937 PMC3195062

[B115] Gray D. A. Wenzel M. (2020). Multitarget approaches against multiresistant superbugs. *ACS Infect. Dis.* 6 1346–1365. 10.1021/acsinfecdis.0c00001 32156116 PMC7307902

[B116] Greenstein G. (1993). The role of metronidazole in the treatment of periodontal diseases. *J. Periodontol.* 64 1–15. 10.1902/jop.1993.64.1.1 8426284

[B117] Guateque-Londoño J. F. Serna-Galvis E. A. Lee J. Ávila-Torres Y. P. Torres-Palma R. A. (2024). Intensifying the sonochemical degradation of hydrophilic organic contaminants by organic and inorganic additives. *J. Environ. Manage.* 366:121930. 10.1016/j.jenvman.2024.121930 39053376

[B118] Guo X. Liu M. Zhong H. Li P. Zhang C. Wei D. et al. (2020). Potential of Myriophyllum aquaticum for phytoremediation of water contaminated with tetracycline antibiotics and copper. *J. Environ. Manage.* 270:110867. 10.1016/j.jenvman.2020.110867 32507744

[B119] Gürtekin E. Çelik M. Aydın E. Çelik A. (2022). Degradation and mineralization of tetracycline by Fenton process. *Environ. Res. Technol.* 5 181–187. 10.35208/ert.1088757

[B120] Gworek B. Kijeńska M. Wrzosek J. Graniewska M. (2021). Pharmaceuticals in the soil and plant environment: A review. *Water Air Soil Pollut.* 232:145. 10.1007/s11270-020-04954-8

[B121] Hampu N. Werber J. R. Chan W. Y. Feinberg E. C. Hillmyer M. A. (2020). Next-generation ultrafiltration membranes enabled by block polymers. *ACS Nano* 14 16446–16471. 10.1021/acsnano.0c07883 33315381

[B122] Hasan T. H. Al-Harmoosh R. A. (2020). Mechanisms of antibiotics resistance in bacteria. *Syst. Rev. Pharm.* 11 817–823. 10.31838/srp.2020.6.118

[B123] Hentschel D. M. Park K. M. Cilenti L. Zervos A. S. Drummond I. Bonventre J. V. (2005). Acute renal failure in zebrafish: A novel system to study a complex disease. *Am. J. Physiol. Renal Physiol.* 288 F923–F929. 10.1152/ajprenal.00386.2004 15625083

[B124] Hong Y. Li H. Chen L. Su H. Zhang B. Luo Y. et al. (2024). Short-term exposure to antibiotics begets long-term disturbance in gut microbial metabolism and molecular ecological networks. *Microbiome* 12:80. 10.1186/s40168-024-01795-z 38715137 PMC11075301

[B125] Hopkins Z. R. Blaney L. (2014). A novel approach to modeling the reaction kinetics of tetracycline antibiotics with aqueous ozone. *Sci. Total Environ.* 468 337–344. 10.1016/j.scitotenv.2013.08.032 24041601

[B126] Hotopp J. C. D. (2011). Horizontal gene transfer between bacteria and animals. *Trends Genet.* 27 157–163. 10.1016/j.tig.2011.01.005 21334091 PMC3068243

[B127] Hou C. Su J. Fan Y. Wang Z. Liu S. Ali A. (2024). Degradation of tetracycline based on activated persulfate by microbial-induced calcium carbonate precipitation as templates modified sludge biochar. *J. Water Process Eng.* 60:105193. 10.1016/j.jwpe.2024.105193

[B128] Hrncir T. (2022). Gut microbiota dysbiosis: Triggers, consequences, diagnostic and therapeutic options. *Microorganisms* 10:578. 10.3390/microorganisms10030578 35336153 PMC8954387

[B129] Huang R. Liu W. Su J. Li S. Wang L. Jeppesen E. et al. (2023). Keystone microalgae species determine the removal efficiency of sulfamethoxazole: A case study of *Chlorella pyrenoidosa* and microalgae consortia. *Front. Plant Sci.* 14:1193668. 10.3389/fpls.2023.1193668 37476166 PMC10354436

[B130] Huang S. Yu J. Li C. Zhu Q. Zhang Y. Lichtfouse E. et al. (2022). The effect review of various biological, physical and chemical methods on the removal of antibiotics. *Water* 14:3138. 10.3390/w14193138

[B131] Hutchings M. I. Truman A. W. Wilkinson B. (2019). Antibiotics: Past, present and future. *Curr. Opin. Microbiol.* 51 72–80. 10.1016/j.mib.2019.10.008 31733401

[B132] Iakovides I. C. Michael-Kordatou I. Moreira N. F. Ribeiro A. R. Fernandes T. Pereira M. F. R. et al. (2019). Continuous ozonation of urban wastewater: Removal of antibiotics, antibiotic-resistant *Escherichia coli* and antibiotic resistance genes and phytotoxicity. *Water Res.* 159 333–347. 10.1016/j.watres.2019.05.025 31108362

[B133] Ives A. M. Brenn-White M. Buckley J. Y. Kendall C. J. Wilton S. Deem S. L. (2022). A global review of causes of morbidity and mortality in free-living vultures. *EcoHealth* 19 40–54. 10.1007/s10393-021-01573-5 35000042

[B134] Janusauskaite D. (2023). The allelopathic activity of aqueous extracts of Helianthus annuus L., grown in boreal conditions, on germination, development, and physiological indices of *Pisum sativum* L. *Plants* 12:1920. 10.3390/plants12091920 37176978 PMC10180669

[B135] Jayalakshmi K. Paramasivam M. Sasikala M. Tamilam T. V. Sumithra A. (2017). Review on antibiotic residues in animal products and its impact on environments and human health. *J. Entomol. Zool. Stud.* 5 1446–1451.

[B136] Ji J. Gao T. Salama E. S. El-Dalatony M. M. Peng L. Gong Y. et al. (2021). Using *Aspergillus niger* whole-cell biocatalyst mycelial aerobic granular sludge to treat pharmaceutical wastewater containing β-lactam antibiotics. *Chem. Eng. J.* 412:128665. 10.1016/j.cej.2021.128665

[B137] Jiang Y. Ran J. Mao K. Yang X. Zhong L. Yang C. et al. (2022). Recent progress in Fenton/Fenton-like reactions for the removal of antibiotics in aqueous environments. *Ecotoxicol. Environ. Saf.* 236:113464. 10.1016/j.ecoenv.2022.113464 35395600

[B138] Kanakaraju D. Yahya M. S. Wong S. P. (2019). Removal of chemical oxygen demand from agro effluent by ZnO photocatalysis and photo-Fenton. *SN Appl. Sci.* 1:738. 10.1007/s42452-019-0782-z

[B139] Kato H. Tsuji K. Harada K. I. (2009). Microbial degradation of cyclic peptides produced by bacteria. *J. Antibiot.* 62 181–190. 10.1038/ja.2009.8 19218981

[B140] Kennes-Veiga D. M. Trueba-Santiso A. Gallardo-Garay V. Balboa S. Carballa M. Lema J. M. (2022). Sulfamethoxazole enhances specific enzymatic activities under aerobic heterotrophic conditions: A metaproteomic approach. *Environ. Sci. Technol.* 56 13152–13159. 10.1021/acs.est.2c05001 36073795 PMC9686132

[B141] Khalifa S. M. Abd El-Aziz A. M. Hassan R. Abdelmegeed E. S. (2021). β-lactam resistance associated with β-lactamase production and porin alteration in clinical isolates of E. coli and *K. pneumoniae*. *PLoS One* 16:e0251594. 10.1371/journal.pone.0251594 34014957 PMC8136739

[B142] Khan A. R. Ulhassan Z. Li G. Lou J. Iqbal B. Salam A. et al. (2024). Micro/nanoplastics: Critical review of their impacts on plants, interactions with other contaminants (antibiotics, heavy metals, and polycyclic aromatic hydrocarbons), and management strategies. *Sci. Total Environ.* 912:169420. 10.1016/j.scitotenv.2023.169420 38128670

[B143] Khan F. Khan M. A. Ahmed N. Khan M. I. Bashir H. Tahir S. et al. (2018). Molecular characterization of pneumococcal surface protein A (PspA), serotype distribution and antibiotic susceptibility of *Streptococcus pneumoniae* strains isolated from Pakistan. *Infect. Dis. Ther.* 7 277–289. 10.1007/s40121-018-0195-0 29524198 PMC5986679

[B144] Khan M. H. Bae H. Jung J. Y. (2010). Tetracycline degradation by ozonation in the aqueous phase: Proposed degradation intermediates and pathway. *J. Hazard. Mater.* 181 659–665. 10.1016/j.jhazmat.2010.05.063 20557998

[B145] Khan M. I. Shin J. H. Kim J. D. (2018). The promising future of microalgae: Current status, challenges, and optimization of a sustainable and renewable industry for biofuels, feed, and other products. *Microb. Cell Fact.* 17:36. 10.1186/s12934-018-0879-x 29506528 PMC5836383

[B146] Khan S. U. Khan M. U. Kalsoom F. Khan M. I. Gao S. Unar A. et al. (2022). Mechanisms of gene regulation by histone degradation in adaptation of yeast: An overview of recent advances. *Arch. Microbiol.* 204:287. 10.1007/s00203-022-02897-8 35482104

[B147] Khan S. Masoodi T. H. Pala N. A. Murtaza S. Mugloo J. A. Sofi P. A. et al. (2023). Phytoremediation prospects for restoration of contamination in the natural ecosystems. *Water* 15:1498. 10.3390/w15081498

[B148] Kim S. W. Lee J. S. Park S. B. Lee A. R. Jung J. W. Chun J. H. et al. (2020). The importance of porins and β-lactamase in outer membrane vesicles on the hydrolysis of β-lactam antibiotics. *Int. J. Mol. Sci.* 21:2822. 10.3390/ijms21082822 32316670 PMC7215730

[B149] Kireeva N. A. Sokolov S. S. Smirnova E. A. Galkina K. V. Severin F. F. Knorre D. A. (2021). Adaptive role of cell death in yeast communities stressed with macrolide antifungals. *mSphere* 6:e0074521. 10.1128/msphere.00745-21 34787448 PMC8597739

[B150] Kirst H. A. (2013). “Macrolide antibiotics,” in *Antimicrobials: New and Old Molecules in the Fight against Multi-Resistant Bacteria*, eds Marinelli F. Genilloud O. (Berlin: Springer), 211–230. 10.1007/978-3-642-39968-8_11

[B151] Kochi L. Y. Kitamura R. S. A. Rocha C. S. Brito J. C. M. Juneau P. Gomes M. P. (2023). Synergistic removal of Ciprofloxacin and sulfamethoxazole by Lemna minor and *Salvinia molesta* in mixed culture: Implications for phytoremediation of antibiotic-contaminated water. *Water* 15:1899. 10.3390/w15101899

[B152] Kourbeti I. S. Alegakis D. E. Maraki S. Samonis G. (2010). Impact of prolonged treatment with high-dose ciprofloxacin on human gut flora: A case report. *J. Med. Case Rep.* 4:111. 10.1186/1752-1947-4-111 20409330 PMC2877052

[B153] Krasucka P. Pan B. Sik Ok Y. Mohan D. Sarkar B. Oleszczuk P. (2021). Engineered biochar – A sustainable solution for the removal of antibiotics from water. *Chem. Eng. J.* 405:126926. 10.1016/j.cej.2020.126926

[B154] Kuppusamy S. Kakarla D. Venkateswarlu K. Megharaj M. Yoon Y. E. Lee Y. B. (2018). Veterinary antibiotics (VAs) contamination as a global agro-ecological issue: A critical view. *Agric. Ecosyst. Environ.* 257 47–59. 10.1016/j.agee.2018.01.026

[B155] Kwon S. J. Na D. H. Kwak J. H. Douaisi M. Zhang F. Park E. J. et al. (2017). Nanostructured glycan architecture is important in the inhibition of influenza A virus infection. *Nat. Nanotechnol.* 12 48–54. 10.1038/nnano.2016.181 27775724

[B156] Labella A. Molero R. Leiva-Rebollo R. Pérez-Recuerda R. Borrego J. J. (2021). Identification, resistance to antibiotics and biofilm formation of bacterial strains isolated from a reverse osmosis system of a drinking water treatment plant. *Sci. Total Environ.* 774:145718. 10.1016/j.scitotenv.2021.145718

[B157] Leanse L. G. Dos Anjos C. Mushtaq S. Dai T. (2022). Antimicrobial blue light: A ‘Magic Bullet’ for the 21st century and beyond? *Adv. Drug Deliv. Rev.* 180:114057. 10.1016/j.addr.2021.114057 34800566 PMC8728809

[B158] Le-Minh N. Khan S. J. Drewes J. E. Stuetz R. M. (2010). Fate of antibiotics during municipal water recycling treatment processes. *Water Res.* 44 4295–4323. 10.1016/j.watres.2010.06.020 20619433

[B159] Lerminiaux N. A. Cameron A. D. S. (2019). Horizontal transfer of antibiotic resistance genes in clinical environments. *Can. J. Microbiol.* 65 34–44. 10.1139/cjm-2018-0275 30248271

[B160] Li G. Yang H. An T. Lu Y. (2018). Antibiotics elimination and risk reduction at two drinking water treatment plants by using different conventional treatment techniques. *Ecotoxicol. Environ. Saf.* 158 154–161. 10.1016/j.ecoenv.2018.04.019 29684745

[B161] Li L. Mao Y. Dong H. Wang Y. Xu L. Liu S. et al. (2023). The ultrafiltration process enhances antibiotic removal in the full-scale advanced treatment of drinking water. *Engineering* 28 16–20. 10.1016/j.eng.2022.05.005

[B162] Li S. Show P. L. Ngo H. H. Ho S. H. (2022). Algae-mediated antibiotic wastewater treatment: A critical review. *Environ. Sci. Ecotechnol.* 9:100145. 10.1016/j.ese.2022.100145 36157853 PMC9488067

[B163] Li W. Liu K. Min Z. Li J. Zhang M. Korshin G. V. et al. (2023). Transformation of macrolide antibiotics during chlorination process: Kinetics, degradation products, and comprehensive toxicity evaluation. *Sci. Total Environ.* 858:159800. 10.1016/j.scitotenv.2022.159800 36309261

[B164] Li X. Nikaido H. (2016). “Efflux-mediated antimicrobial resistance in bacteria,” in *Efflux-Mediated Antimicrobial Resistance in Bacteria*, eds Li X. Z. Elkins C. A. Zgurskaya H. I. (Berlin: Springer). 10.1007/978-3-319-39658-3

[B165] Li Z. H. Yuan L. Gao S. X. Wang L. Sheng G. P. (2019). Mitigated membrane fouling and enhanced removal of extracellular antibiotic resistance genes from wastewater effluent via an integrated pre-coagulation and microfiltration process. *Water Res.* 159 145–152. 10.1016/j.watres.2019.05.005 31085389

[B166] Ligozzi M. Pittaluga F. Fontana R. (1993). Identification of a genetic element (psr) which negatively controls expression of *Enterococcus hirae* penicillin-binding protein 5. *J. Bacteriol.* 175 2046–2051. 10.1128/jb.175.7.2046-2051.1993 8458847 PMC204297

[B167] Lima L. M. da Silva B. N. M. Barbosa G. Barreiro E. J. (2020). β-lactam antibiotics: An overview from a medicinal chemistry perspective. *Eur. J. Med. Chem.* 208:112829. 10.1016/j.ejmech.2020.112829 33002736

[B168] Limbu S. M. Chen L. Q. Zhang M. L. Du Z. Y. (2021). A global analysis on the systemic effects of antibiotics in cultured fish and their potential human health risk: A review. *Rev. Aquac.* 13 1015–1059. 10.1111/raq.12511

[B169] Limbu S. M. Zhang H. Luo Y. Chen L. Q. Zhang M. Du Z. Y. (2020). High carbohydrate diet partially protects Nile tilapia (*Oreochromis niloticus*) from oxytetracycline-induced side effects. *Environ. Pollut.* 256:113508. 10.1016/j.envpol.2019.113508 31706777

[B170] Lin J. Zhang K. Jiang L. Hou J. Yu X. Feng M. et al. (2022). Removal of chloramphenicol antibiotics in natural and engineered water systems: Review of reaction mechanisms and product toxicity. *Sci. Total Environ.* 850:158059. 10.1016/j.scitotenv.2022.158059 35985581

[B171] Lin X. Kück U. (2022). Cephalosporins as key lead generation beta-lactam antibiotics. *Appl. Microbiol. Biotechnol.* 106 8007–8020. 10.1007/s00253-022-12272-8 36401643 PMC9712332

[B172] Lin Y. C. Hsiao K. W. Lin A. Y. C. (2018). Photolytic degradation of ciprofloxacin in solid and aqueous environments: Kinetics, phototransformation pathways, and byproducts. *Environ. Sci. Pollut. Res.* 25 2303–2312. 10.1007/s11356-017-0666-y 29119496

[B173] Lin Y. Wang Y. Shi C. Zhang D. Liu G. Chen L. et al. (2023). Degradation of ciprofloxacin by a constitutive gC 3 N 4/BiOCl heterojunction under a persulfate system. *RSC Adv.* 13 4361–4375. 10.1039/D2RA06500B 36760283 PMC9892887

[B174] Liu F. Shen Y. Hou Y. Wu J. Ting Y. Nie C. et al. (2024). Elimination of representative antibiotic-resistant bacteria, antibiotic resistance genes and ciprofloxacin from water via photoactivation of periodate using FeS2. *J. Hazard. Mater.* 476:134982. 10.1016/j.jhazmat.2024.134982 38917629

[B175] Liu P. Wu Z. Abramova A. V. Cravotto G. (2021). Sonochemical processes for the degradation of antibiotics in aqueous solutions: A review. *Ultrason. Sonochem.* 74:105566. 10.1016/j.ultsonch.2021.105566 33975189 PMC8122362

[B176] Liu S. Le Mauff F. Sheppard D. C. Zhang S. (2022). Filamentous fungal biofilms: Conserved and unique aspects of extracellular matrix composition, mechanisms of drug resistance and regulatory networks in *Aspergillus fumigatus*. *NPJ Biofilms Microbiomes* 8:83. 10.1038/s41522-022-00347-3 36261442 PMC9581972

[B177] Liu Y. Wang F. Chen X. Zhang J. Gao B. (2015). Cellular responses and biodegradation of amoxicillin in *Microcystis aeruginosa* at different nitrogen levels. *Ecotoxicol. Environ. Saf.* 111 138–145. 10.1016/j.ecoenv.2014.10.011 25450926

[B178] Lunara S. Pavelquesi S. Carolina A. de Oliveira A. Ricelle A. Maria C. et al. (2021). Presence of tetracycline and sulfonamide resistance genes in *Salmonella* spp.: Literature review. *Antibiotics* 10:1314. 10.3390/antibiotics10111314 34827252 PMC8615168

[B179] Luthra S. Rominski A. Sander P. (2018). The role of antibiotic-target-modifying and antibiotic-modifying enzymes in mycobacterium abscessusdrug resistance. *Front. Microbiol.* 9:2179. 10.3389/fmicb.2018.02179 30258428 PMC6143652

[B180] Ma J. Jiang Z. Cao J. Yu F. (2020). Enhanced adsorption for the removal of antibiotics by carbon nanotubes/graphene oxide/sodium alginate triple-network nanocomposite hydrogels in aqueous solutions. *Chemosphere* 242:125188. 10.1016/j.chemosphere.2019.125188 31675580

[B181] Majeed W. Ahmed H. S. Hamed Z. A. (2024). Development of tetracycline by AgO nanoparticles and studying its activity on antibiotic-resistant bacteria. *Acad. Open* 9 10–21070. 10.21070/acopen.9.2024.10275

[B182] Malik S. N. Ghosh P. C. Vaidya A. N. Mudliar S. N. (2020). Hybrid ozonation process for industrial wastewater treatment: Principles and applications: A review. *J. Water Process Eng.* 35:101193. 10.1016/j.jwpe.2020.101193

[B183] Manasfi R. Chiron S. Montemurro N. Perez S. Brienza M. (2020). Biodegradation of fluoroquinolone antibiotics and the climbazole fungicide by Trichoderma species. *Environ. Sci. Pollut. Res.* 27 23331–23341. 10.1007/s11356-020-08442-8 32337674

[B184] Manna M. S. Tamer Y. T. Gaszek I. Poulides N. Ahmed A. Wang X. et al. (2021). A trimethoprim derivative impedes antibiotic resistance evolution. *Nat. Commun.* 12:2949. 10.1038/s41467-021-23191-z 34011959 PMC8134463

[B185] Markley J. L. Wencewicz T. A. (2018). Tetracycline-inactivating enzymes. *Front. Microbiol.* 9:1058. 10.3389/fmicb.2018.01058 29899733 PMC5988894

[B186] Mathur P. Sanyal D. Callahan D. L. Conlan X. A. Pfeffer F. M. (2021). Treatment technologies to mitigate the harmful effects of recalcitrant fluoroquinolone antibiotics on the environment and human health. *Environ. Pollut.* 291:118233. 10.1016/j.envpol.2021.118233 34582925

[B187] McLeod D. C. Lyon J. A. (1985). Imipenem/cilastatin: The first carbapenem antibiotic. *Drug Intell. Clin. Pharm.* 19 894–899. 10.1177/106002808501901202 3910385

[B188] Merkus V. I. Leupold M. S. Rockel S. P. Schmidt T. C. (2023). Insights into the ozonation of antiviral purine derivatives by the basic Structures’ kinetics and ozone consumption ratios. *ACS ES&T Water* 3 2363–2372. 10.1021/acsestwater.3c00086

[B189] Miklasińska-Majdanik M. (2021). Mechanisms of resistance to macrolide antibiotics among *Staphylococcus aureus*. *Antibiotics* 10:1406. 10.3390/antibiotics10111406 34827344 PMC8615237

[B190] Minden V. Scherber C. Cebrián Piqueras M. A. Trinogga J. Trenkamp A. Mantilla-Contreras J. et al. (2016). Consistent drivers of plant biodiversity across managed ecosystems. *Philos. Trans. R. Soc. B Biol. Sci.* 371:20150284. 10.1098/rstb.2015.0284 27114585 PMC4843704

[B191] Mishra M. K. Kumar A. (2024). “Fluxomics and metabolic flux analysis,” in *Multi-Omics Analysis of the Human Microbiome: From Technology to Clinical Applications*, eds Mani I. Singh V. (Singapore: Springer Nature Singapore), 171–180.

[B192] Montoya-Rodríguez D. M. Serna-Galvis E. A. Ferraro F. Torres-Palma R. A. (2020). Degradation of the emerging concern pollutant ampicillin in aqueous media by sonochemical advanced oxidation processes - Parameters effect, removal of antimicrobial activity and pollutant treatment in hydrolyzed urine. *J. Environ. Manage.* 261:110224. 10.1016/j.jenvman.2020.110224 32148294

[B193] Mora-Gamboa M. P. C. Rincón-Gamboa S. M. Ardila-Leal L. D. Poutou-Piñales R. A. Pedroza-Rodríguez A. M. Quevedo-Hidalgo B. E. (2022). Impact of antibiotics as waste, physical, chemical, and enzymatical degradation: Use of laccases. *Molecules* 27:4436. 10.3390/molecules27144436 35889311 PMC9319608

[B194] Morales-Alvarez M. C. (2020). Nephrotoxicity of antimicrobials and antibiotics. *Adv. Chronic Kidney Dis.* 27 31–37. 10.1053/j.ackd.2019.08.001 32146999

[B195] Navada K. K. Kulal A. (2019). Enzymatic degradation of chloramphenicol by laccase from *Trametes hirsuta* and comparison among mediators. *Int. Biodeterior. Biodegrad.* 138 63–69. 10.1016/j.ibiod.2018.12.012

[B196] Nelson M. L. Levy S. B. (2011). The history of the tetracyclines. *Ann. N. Y. Acad. Sci.* 1241 17–32. 10.1111/j.1749-6632.2011.06354.x 22191524

[B197] Nguyen D. N. Bui H. M. Nguyen H. Q. (2020). “Heterogeneous photocatalysis for the removal of pharmaceutical compounds,” in *Current Developments in Biotechnology and Bioengineering: Emerging Organic Micro-pollutants*, eds Varjani S. Pandey A. Tyagi R. D. Ngo H. H. Larroche C. (Amsterdam: Elsevier B.V). 10.1016/B978-0-12-819594-9.00007-3

[B198] Nicoloff H. Andersson D. I. (2016). Indirect resistance to several classes of antibiotics in cocultures with resistant bacteria expressing antibiotic-modifying or-degrading enzymes. *J. Antimicrob. Chemother.* 71 100–110. 10.1093/jac/dkv312 26467993

[B199] Nwani C. D. Mkpadobi B. N. Onyishi G. Echi P. C. Chukwuka C. O. Oluah S. N. et al. (2014). Changes in behavior and hematological parameters of freshwater African catfish *Clarias gariepinus* (Burchell 1822) following sublethal exposure to chloramphenicol. *Drug Chem. Toxicol.* 37 107–113. 10.3109/01480545.2013.834348 24099453

[B200] Okaiyeto S. A. Sutar P. P. Chen C. Ni J. B. Wang J. Mujumdar A. S. et al. (2024). Antibiotic resistant bacteria in food systems: Current status, resistance mechanisms, and mitigation strategies. *Agric. Commun.* 2:100027. 10.1016/j.agrcom.2024.100027

[B201] Oladeji O. M. Mugivhisa L. L. Olowoyo J. O. (2025). Antibiotic residues in animal products from some African countries and their possible impact on human health. *Antibiotics* 14:90. 10.3390/antibiotics14010090 39858375 PMC11759178

[B202] Oliveira M. Leonardo I. C. Silva A. F. Crespo J. G. Nunes M. Crespo M. T. B. (2022). Nanofiltration as an efficient tertiary wastewater treatment: Elimination of total bacteria and antibiotic resistance genes from the discharged effluent of a full-scale wastewater treatment plant. *Antibiotics* 11:630. 10.3390/antibiotics11050630 35625274 PMC9137456

[B203] Oliveira R. McDonough S. Ladewig J. C. Soares A. M. Nogueira A. J. Domingues I. (2013). Effects of oxytetracycline and amoxicillin on development and biomarkers activities of zebrafish (*Danio rerio*). *Environ. Toxicol. Pharmacol.* 36 903–912. 10.1016/j.etap.2013.07.019 24008007

[B204] Ormeno-Cano N. Radjenovic J. (2022). Electrochemical degradation of antibiotics using flow-through graphene sponge electrodes. *J. Hazard. Mater.* 431:128462. 10.1016/j.jhazmat.2022.128462 35220123

[B205] Ota Y. Chen F. Prah I. Mahazu S. Watanabe K. Kinoshita T. et al. (2024). Metatranscriptomic analysis reveals actively expressed antimicrobial-resistant genes and their hosts in hospital wastewater. *Antibiotics* 13:1122. 10.3390/antibiotics13121122 39766512 PMC11672649

[B206] Pak G. Salcedo D. E. Lee H. Oh J. Maeng S. K. Song K. G. et al. (2016). Comparison of antibiotic resistance removal efficiencies using ozone disinfection under different pH and suspended solids and humic substance concentrations. *Environ. Sci. Technology* 50 7590–7600. 10.1021/acs.est.6b01340 27389869

[B207] Palacio D. A. Leiton L. M. Urbano B. F. Rivas B. L. (2020). Tetracycline removal by polyelectrolyte copolymers in conjunction with ultrafiltration membranes through liquid-phase polymer-based retention. *Environ. Res.* 182:109014. 10.1016/j.envres.2019.109014 31846895

[B208] Pandis P. K. Kalogirou C. Kanellou E. Vaitsis C. Savvidou M. G. Sourkouni G. et al. (2022). Key points of advanced oxidation processes (AOPs) for wastewater, organic pollutants and pharmaceutical waste treatment: A mini review. *ChemEngineering* 6:8. 10.3390/chemengineering6010008

[B209] Pang Z. Raudonis R. Glick B. R. Lin T. J. Cheng Z. (2019). Antibiotic resistance in *Pseudomonas aeruginosa*: Mechanisms and alternative therapeutic strategies. *Biotechnol. Adv.* 37 177–192. 10.1016/j.biotechadv.2018.11.013 30500353

[B210] Paquin F. Rivnay J. Salleo A. Stingelin N. Silva C. (2015). Multi-phase semicrystalline microstructures drive exciton dissociation in neat plastic semiconductors. *J. Mater. Chem. C* 3 10715–10722. 10.1039/b000000x 41614145 PMC12851566

[B211] Park H. Choung Y. K. (2013). Evaluation of the biodegradation feasibility of antibiotics by three bacteria involving glutathione S-transferases. *J. Environ. Eng. Sci.* 8 550–555. 10.1680/jees.2013.0057 26962031

[B212] Patangia D. V. Anthony Ryan C. Dempsey E. Paul Ross R. Stanton C. (2022). Impact of antibiotics on the human microbiome and consequences for host health. *MicrobiologyOpen* 11:e1260. 10.1002/mbo3.1260 35212478 PMC8756738

[B213] Pereira A. K. S. Silva L. F. Barbosa G. A. F. Miranda T. G. Sousa R. R. Sarmento R. A. et al. (2023). The socio-environmental and human health problems related to the use of pesticides and the use of advanced oxidative processes for their degradation: Brazil. *Water* 15:1608. 10.3390/w15081608

[B214] Pham C. M. Pham N. Q. Le K. A. (2021). Treatment of antibiotic residues of fluoroquinolones (Ofloxacin) in hospital wastewater using peroxone oxidation process. *Chem. Eng. Trans.* 89 211–216. 10.3303/CET2189036

[B215] Pham T. D. Ziora Z. M. Blaskovich M. A. (2019). Quinolone antibiotics. *Medchemcomm* 10 1719–1739. 10.1039/c9md00120d 31803393 PMC6836748

[B216] Philippon A. Slama P. Dény P. Labia R. (2016). A structure-based classification of class A β-lactamases, a broadly diverse family of enzymes. *Clin. Microbiol. Rev.* 29 29–57. 10.1128/cmr.00019-15 26511485 PMC4771212

[B217] Polianciuc S. I. Gurzãu A. E. Kiss B. Ştefan M. G. Loghin F. (2020). Antibiotics in the environment: Causes and consequences. *Med. Pharm. Rep.* 93 231–240. 10.15386/mpr-1742 32832887 PMC7418837

[B218] Qi X. Ru S. Xiong J. Q. (2022). Ecotoxicological effects of sulfacetamide on a green microalga, *Desmodesmus quadricauda*: Cell viability, antioxidant system, and biotransformation. *Environ. Technol. Innov.* 26:102278. 10.1016/j.eti.2022.102278

[B219] Qiu G. Chen H. Srinivasa Raghavan D. S. Ting Y. P. (2021). Removal behaviors of antibiotics in a hybrid microfiltration-forward osmotic membrane bioreactor for real municipal wastewater treatment. *Chem. Eng. J.* 417:129146. 10.1016/j.cej.2021.129146

[B220] Randhawa A. Pasari N. Sinha T. Gupta M. Nair A. M. Ogunyewo O. A. et al. (2021). Blocking drug efflux mechanisms facilitate genome engineering process in hypercellulolytic fungus, *Penicillium funiculosum* NCIM1228. *Biotechnol. Biofuels* 14:31. 10.1186/s13068-021-01883-4 33494787 PMC7836482

[B221] Rathman B. M. Del Valle J. R. (2022). Late-stage sidechain-to-backbone macrocyclization of N-amino peptides. *Org. Lett.* 24 1536–1540. 10.1021/acs.orglett.2c00204 35157469

[B222] Raval H. D. Parmar P. Raval K. (2024). Micellar-enhanced ultrafiltration with a novel modified membrane for removal of arsenate and emerging contaminants from water. *Desalination* 574:117230. 10.1016/j.desal.2023.117230

[B223] Raymond F. Ouameur A. A. Déraspe M. Iqbal N. Gingras H. Dridi B. et al. (2016). The initial state of the human gut microbiome determines its reshaping by antibiotics. *ISME J.* 10 707–720. 10.1038/ismej.2015.148 26359913 PMC4817689

[B224] Rehman J. U. Joe E. N. Yoon H. Y. Kwon S. Oh M. S. Son E. J. et al. (2022). Lignin metabolism by selected fungi and microbial consortia for plant stimulation: Implications for biologically active humus genesis. *Microbiol. Spectr.* 10:e2637-22. 10.1128/spectrum.02637-22 36314978 PMC9769858

[B225] Reis A. C. Kolvenbach B. A. Nunes O. C. Corvini P. F. X. (2020). Biodegradation of antibiotics: The new resistance determinants – part II. *New Biotechnol.* 54 13–27. 10.1016/j.nbt.2019.08.003 31419608

[B226] Ren C. Li J. Zhang X. Niu Y. (2023). Photocatalytic degradation of ciprofloxacin with supramolecular materials consisting of nitrogenous organic cations and metal salts. *Catalysts* 13:1134. 10.3390/catal13071134

[B227] Ren J. Wang Z. Deng L. Niu D. Huhetaoli, Li Z. et al. (2021). Degradation of erythromycin by a novel fungus, *Penicillium oxalicum* RJJ-2, and the degradation pathway. *Waste Biomass Valoriz.* 12 4513–4523. 10.1007/s12649-021-01343-y

[B228] Ren Z. Li H. Luo W. (2024). Unraveling the mystery of antibiotic resistance genes in green and red Antarctic snow. *Sci. Total Environ.* 915 170148. 10.1016/j.scitotenv.2024.170148 38246373

[B229] Rice L. B. Carias L. L. Hutton-Thomas R. Sifaoui F. Gutmann L. Rudin S. D. (2001). Penicillin-binding protein 5 and expression of ampicillin resistance in *Enterococcus faecium*. *Antimicrob. Agents Chemother.* 45 1480–1486. 10.1128/AAC.45.5.1480-1486.2001 11302814 PMC90492

[B230] Rocha A. J. De Oliveira Barsottini M. R. Rocha R. R. Laurindo M. V. De Moraes F. L. L. Da Rocha S. L. (2019). *Pseudomonas aeruginosa*: Virulence factors and antibiotic resistance Genes. *Braz. Arch. Biol. Technol.* 62:e19180503. 10.1590/1678-4324-2019180503

[B231] Rubinstein E. Keynan Y. (2014). Vancomycin revisited–60 years later. *Front. Public Health* 2:217. 10.3389/fpubh.2014.00217 25401098 PMC4215627

[B232] Saha A. Varanasi S. (2024). Sunlight-assisted photocatalytic degradation of azithromycin using cellulose nanocrystals–TiO2 composites. *Appl. Nanosci.* 14 675–686. 10.1007/s13204-024-03039-w

[B233] Samraj J. J. Manju R. Neppolian B. (2024). Sonochemical degradation of tetracycline and pharmaceutical effluent from the aqueous environment: Maximizing efficiency with S-scheme photocatalyst. *J. Water Process Eng.* 68:106487. 10.1016/j.jwpe.2024.106487

[B234] Sanahuja-Embuena V. Frauholz J. Oruc T. Trzaskus K. Hélix-Nielsen C. (2021). Transport mechanisms behind enhanced solute rejection in forward osmosis compared to reverse osmosis mode. *J. Membr. Sci.* 636:119561. 10.1016/j.memsci.2021.119561

[B235] Santhaseelan H. Dinakaran V. T. Sakthivel B. Somasundaram M. Thanamegam K. Devendiran V. et al. (2022). Bioactive efficacy of novel carboxylic acid from halophilic *Pseudomonas aeruginosa* against methicillin-resistant *Staphylococcus aureus*. *Metabolites* 12:1094. 10.3390/metabo12111094 36355177 PMC9698732

[B236] Sarasti J. F. Calispa N. S. G. Hidalgo E. S. B. Jaramillo A. P. H. (2025). Impact of antibiotic-induced dysbiosis in childhood: Effects on immunological development and the risk of chronic diseases such as asthma, allergies, and obesity. *RECIMUNDO* 9 1064–1077. 10.26820/recimundo/9.(1).enero.2025.1064-1077

[B237] Saravanan A. Kumar P. S. Varjani S. Jeevanantham S. Yaashikaa P. R. Thamarai P. et al. (2021). A review on algal-bacterial symbiotic system for effective treatment of wastewater. *Chemosphere* 271:129540. 10.1016/j.chemosphere.2021.129540 33434824

[B238] Sartini S. Permana A. D. Mitra S. Tareq A. M. Salim E. Ahmad I. et al. (2021). Current state and promising opportunities on pharmaceutical approaches in the treatment of polymicrobial diseases. *Pathogens* 10:245. 10.3390/pathogens10020245 33672615 PMC7924209

[B239] Satulu V. Pandele A. M. Ionica G. I. Bobiricã L. Bonciu A. F. Scarlatescu A. et al. (2024). Robust CA-GO-TiO2/PTFE photocatalytic membranes for the degradation of the azithromycin formulation from wastewaters. *Polymers* 16:1368. 10.3390/polym16101368 38794561 PMC11125009

[B240] Sayadi M. H. Sobhani S. Shekari H. (2019). Photocatalytic degradation of azithromycin using GO@ Fe3O4/ZnO/SnO2 nanocomposites. *J. Clean. Prod.* 232 127–136. 10.1016/J.JCLEPRO.2019.05.338

[B241] Schüttel M. (2023). Development of Methods for the Synthesis of Large Combinatorial Libraries of Macrocyclic Compounds (No. 9951). EPFL. Available online at: https://infoscience.epfl.ch/handle/20.500.14299/197677 (accessed May 15, 2023).

[B242] Schwartz D. J. Langdon A. E. Dantas G. (2020). Understanding the impact of antibiotic perturbation on the human microbiome. *Genome Med.* 12:82. 10.1186/s13073-020-00782-x 32988391 PMC7523053

[B243] Seifrtová M. Nováková L. Lino C. Pena A. Solich P. (2009). An overview of analytical methodologies for the determination of antibiotics in environmental waters. *Anal. Chim. Acta* 649 158–179. 10.1016/j.aca.2009.07.031 19699391

[B244] Sekyere J. O. Asante J. (2018). Emerging mechanisms of antimicrobial resistance in bacteria and fungi: Advances in the era of genomics. *Future Microbiol.* 13 241–262. 10.2217/fmb-2017-0172 29319341

[B245] Serna-Galvis E. A. Silva-Agredo J. Giraldo-Aguirre A. L. Flórez-Acosta O. A. Torres-Palma R. A. (2016). High frequency ultrasound as a selective advanced oxidation process to remove penicillinic antibiotics and eliminate its antimicrobial activity from water. *Ultrason. Sonochem.* 31 276–283. 10.1016/j.ultsonch.2016.01.007 26964950

[B246] Sharma G. Sharma S. Sharma P. Chandola D. Dang S. Gupta S. et al. (2016). *Escherichia coli* biofilm: Development and therapeutic strategies. *J. Appl. Microbiol.* 121 309–319. 10.1111/jam.13078 26811181

[B247] Sheikh S. W. Ali A. Ahsan A. Shakoor S. Shang F. Xue T. (2021). Insights into emergence of antibiotic resistance in acid-adapted enterohaemorrhagic *Escherichia coli*. *Antibiotics* 10:522. 10.3390/antibiotics10050522 34063307 PMC8147483

[B248] Shi C. Yu S. Wang L. Zhang X. Lin X. Li C. (2021). Degradation of tetracycline/oxytetracycline by electrospun aligned polyacrylonitrile-based carbon nanofibers as anodic electrocatalysis microfiltration membrane. *J. Environ. Chem. Eng.* 9:106540. 10.1016/j.jece.2021.106540

[B249] Shi Y. Wang X. Feng C. Songtao Z. (2022). Technologies for the removal of antibiotics in the environment: A review. *Int. J. Electrochem. Sci.* 17:220768. 10.20964/2022.07.74

[B250] Shree S. K. Namasivayam S. K. R. Pandian A. (2023). Sustainable developmental measures for the treatment of pharmaceutical industry effluent using nano zero valent iron technology (nZVI)–A review. *J. Water Process Eng.* 56:104390. 10.1016/j.jwpe.2023.104390

[B251] Singh R. Samuel M. S. Ravikumar M. Ethiraj S. Kirankumar V. S. Kumar M. et al. (2023). Processing of carbon-based nanomaterials for the removal of pollutants from water/wastewater application. *Water* 15:3003. 10.3390/w15163003

[B252] Song Y. Meng C. Chen X. Li Y. Ma S. Zhang L. et al. (2022). Synchronous removal of antibiotics in sewage effluents by surface-anchored photocatalytic nanofiltration membrane in a continuous dynamic process. *Environ. Sci. Nano* 10 567–580. 10.1039/d2en00972b

[B253] Stamps B. W. Nunn H. S. Petryshyn V. A. Oremland R. S. Miller L. G. Rosen M. R. et al. (2018). Metabolic capability and phylogenetic diversity of Mono Lake during a bloom of the eukaryotic phototroph *Picocystis* sp. strain ML. *Appl. Environ. Microbiol.* 84:e01171-18. 10.1128/AEM.01171-18 30120120 PMC6193381

[B254] Stando K. Czyż A. Gajda M. Felis E. Bajkacz S. (2022). Study of the Phytoextraction and Phytodegradation of Sulfamethoxazole and Trimethoprim from Water by *Limnobium laevigatum*. *Int. J. Environ. Res. Public Health* 19:16994. 10.3390/ijerph192416994 36554877 PMC9779370

[B255] Stange C. Sidhu J. P. S. Toze S. Tiehm A. (2019). Comparative removal of antibiotic resistance genes during chlorination, ozonation, and UV treatment. *Int. J. Hyg. Environ. Health* 222 541–548. 10.1016/j.ijheh.2019.02.002 30738743

[B256] Stanton I. C. Murray A. K. Zhang L. Snape J. Gaze W. H. (2020). Evolution of antibiotic resistance at low antibiotic concentrations including selection below the minimal selective concentration. *Commun. Biol.* 3:467. 10.1038/s42003-020-01176-w 32884065 PMC7471295

[B257] Stavroulaki E. M. Suchodolski J. S. Pilla R. Fosgate G. T. Sung C. H. Lidbury J. A. et al. (2021). Short-and long-term effects of amoxicillin/clavulanic acid or doxycycline on the gastrointestinal microbiome of growing cats. *PLoS One* 16:e0253031. 10.1371/journal.pone.0253031 34910719 PMC8673677

[B258] Su R. Dai X. Wang H. Wang Z. Li Z. Chen Y. et al. (2022). Metronidazole degradation by UV and UV/H2O2 advanced oxidation processes: Kinetics, mechanisms, and effects of natural water matrices. *Int. J. Environ. Res. Public Health* 19:12354. 10.3390/ijerph191912354 36231654 PMC9565145

[B259] Sun J. Chu R. Khan Z. U. H. (2023). A Theoretical study on the degradation mechanism, kinetics, and ecotoxicity of metronidazole (MNZ) in OH-and SO4–assisted advanced oxidation processes. *Toxics* 11:796. 10.3390/toxics11090796 37755806 PMC10535747

[B260] Sung J. Y. Kwon K. C. Cho H. H. Koo S. H. (2011). Antimicrobial resistance determinants in imipenem-nonsusceptible Acinetobacter calcoaceticus-baumannii complex isolated in Daejeon, Korea. *Korean J. Lab. Med.* 31 265–270. 10.3343/kjlm.2011.31.4.265 22016680 PMC3190005

[B261] Tan H. Kong D. Ma Q. Li Q. Zhou Y. Jiang X. et al. (2022). Biodegradation of tetracycline antibiotics by the yeast strain *Cutaneotrichosporon dermatis* M503. *Microorganisms* 10:565. 10.3390/microorganisms10030565 35336139 PMC8955161

[B262] Tang J. Wang P. Xie Z. Wang Z. Hu B. (2021). Effect of iron plaque on antibiotic uptake and metabolism in water spinach (Ipomoea aquatic Forsk.) grown in hydroponic culture. *J. Hazard. Mater.* 417:125981. 10.1016/j.jhazmat.2021.125981 33975166

[B263] Tao W. Lee M. H. Wu J. Kim N. H. Kim J. C. Chung E. et al. (2012). Inactivation of chloramphenicol and florfenicol by a novel chloramphenicol hydrolase. *Appl. Environ. Microbiol.* 78 6295–6301. 10.1128/AEM.01154-12 22752166 PMC3416615

[B264] Thakre M. B. Kapoor S. B. Gandhare N. (2024). Methods for eliminating micropollutant from wastewater: A review. *Environ. Conserv. J.* 25 267–273. 10.36953/ECJ.26652643

[B265] Thoms D. Liang Y. Haney C. H. (2021). Maintaining symbiotic homeostasis: How do plants engage with beneficial microorganisms while at the same time restricting pathogens? *Mol. Plant Microbe Interact.* 34 462–469. 10.1094/MPMI-11-20-0318-FI 33534602

[B266] Thrastardottir T. O. Copeland V. J. Constantinou C. (2022). The association between the gut microbiome, nutritional habits, antibiotics, and gastric cancer: A scoping review. *Curr. Nutr. Rep.* 11 19–38. 10.1007/s13668-021-00391-z 35020173

[B267] Tian C. Huang W. Wei Z. Liang C. Dong Y. Shi J. et al. (2023). Photocatalytic degradation of different antibiotics using TiO 2–carbon composites: A case study of tetracycline and ciprofloxacin. *New J. Chem.* 47 19646–19656. 10.1039/D3NJ02748A

[B268] Tozar T. Boni M. Staicu A. Pascu M. L. (2021). Optical characterization of ciprofloxacin photolytic degradation by UV-pulsed laser radiation. *Molecules* 26:2324. 10.3390/molecules26082324 33923649 PMC8073987

[B269] Tran K. Moody G. Wu F. Lu X. Choi J. Kim K. et al. (2019). Evidence for moiré excitons in van der Waals heterostructures. *Nature* 567 71–75. 10.1038/s41586-019-0975-z 30804527 PMC11493145

[B270] Tyers M. Wright G. D. (2019). Drug combinations: A strategy to extend the life of antibiotics in the 21st century. *Nat. Rev. Microbiol.* 17 141–155. 10.1038/s41579-018-0141-x 30683887

[B271] Urban-Chmiel R. Marek A. Stȩpień-Pyśniak D. Wieczorek K. Dec M. Nowaczek A. et al. (2022). Antibiotic resistance in bacteria—A review. *Antibiotics* 11:1079. 10.3390/antibiotics11081079 36009947 PMC9404765

[B272] Van der Bruggen B. Mänttäri M. Nyström M. (2008). Drawbacks of applying nanofiltration and how to avoid them: A review. *Sep. Purif. Technol.* 63 251–263. 10.1016/j.seppur.2008.05.010

[B273] Varela M. F. Stephen J. Lekshmi M. Ojha M. Wenzel N. Sanford L. M. et al. (2021). Bacterial resistance to antimicrobial agents. *Antibiotics* 10:593. 10.3390/antibiotics10050593 34067579 PMC8157006

[B274] Vinayamohan P. G. Pellissery A. J. Venkitanarayanan K. (2022). Role of horizontal gene transfer in the dissemination of antimicrobial resistance in food animal production. *Curr. Opin. Food Sci.* 47:100882. 10.1016/j.cofs.2022.100882

[B275] Wang H. Wang Q. Lv M. Song Z. Yu J. Wang X. et al. (2024). Study on the degradation and metabolic mechanism of four quinolone antibiotics by mixed strains. *Front. Environ. Chem.* 5:1326206. 10.3389/fenvc.2024.1326206

[B276] Wang M. Shen W. Yan L. Wang X. H. Xu H. (2017). Stepwise impact of urban wastewater treatment on the bacterial community structure, antibiotic contents, and prevalence of antimicrobial resistance. *Environ. Pollut.* 231 1578–1585. 10.1016/j.envpol.2017.09.055 28967569

[B277] Wang N. Peng L. Gu Y. Liang C. Pott R. W. Xu Y. (2023). Insights into biodegradation of antibiotics during the biofilm-based wastewater treatment processes. *J. Clean. Prod.* 393:136321. 10.1016/j.jclepro.2023.136321

[B278] Wang S. Wang J. (2017). Comparative study on sulfamethoxazole degradation by Fenton and Fe (II)-activated persulfate process. *RSC Adv.* 7 48670–48677. 10.1039/C7RA09325J

[B279] Wang X. Ryu D. Houtkooper R. H. Auwerx J. (2015). Antibiotic use and abuse: A threat to mitochondria and chloroplasts with impact on research, health, and environment. *BioEssays* 37 1045–1053. 10.1002/bies.201500071 26347282 PMC4698130

[B280] Wang X. Wang X. Lynch I. Ma J. (2023). High-efficiency removal of tetracycline from water by electrolysis-assisted NZVI: Mechanism of electron transfer and redox of iron. *RSC Adv.* 13 15881–15891. 10.1039/D3RA00954H 37250228 PMC10213827

[B281] Wang Y. Zhang H. Zhang J. Lu C. Huang Q. Wu J. et al. (2011). Degradation of tetracycline in aqueous media by ozonation in an internal loop-lift reactor. *J. Hazard. Mater.* 192 35–43. 10.1016/j.jhazmat.2011.04.086 21616591

[B282] Wilson D. N. Hauryliuk V. Atkinson G. C. O’Neill A. J. (2020). Target protection as a key antibiotic resistance mechanism. *Nat. Rev. Microbiol.* 18 637–648. 10.1038/s41579-020-0386-z 32587401

[B283] Wohde M. Berkner S. Junker T. Konradi S. Schwarz L. Düring R. A. (2016). Occurrence and transformation of veterinary pharmaceuticals and biocides in manure: A literature review. *Environ. Sci. Eur.* 28 1–25. 10.1186/s12302-016-0091-8 27761355 PMC5044974

[B284] Xiang Y. Xiong W. Xu R. Yang Z. Zhang Y. Jia M. et al. (2023). Metagenomic analysis reveals microbial metabolic potentials alterations under antibiotic stress during sludge anaerobic digestion. *J. Environ. Chem. Eng.* 11:110746. 10.1016/j.jece.2023.110746

[B285] Xing J. Huang J. Wang X. Yang F. Bai Y. Li S. et al. (2023). Removal of low-concentration tetracycline from water by a two-step process of adsorption enrichment and photocatalytic regeneration. *J. Environ. Manage.* 343:118210. 10.1016/j.jenvman.2023.118210 37229865

[B286] Xiong J. Q. Govindwar S. Kurade M. B. Paeng K. J. Roh H. S. Khan M. A. et al. (2019). Toxicity of sulfamethazine and sulfamethoxazole and their removal by a green microalga, *Scenedesmus obliquus*. *Chemosphere* 218 551–558. 10.1016/j.chemosphere.2018.11.146 30500716

[B287] Yang L. Bajinka O. Jarju P. O. Tan Y. Taal A. M. Ozdemir G. (2021). The varying effects of antibiotics on gut microbiota. *AMB Express* 11:116. 10.1186/s13568-021-01274-w 34398323 PMC8368853

[B288] Yazdanpanah G. Heidari M. R. Amirmahani N. Nasiri A. (2023). Heterogeneous Sono-Fenton like catalytic degradation of metronidazole by Fe3O4@ HZSM-5 magnetite nanocomposite. *Heliyon* 9:e16461. 10.1016/j.heliyon.2023.e16461 37292306 PMC10245020

[B289] Yazdi M. Sayadi M. H. Farsad F. (2018). Removal of penicillin in aqueous solution using *Chlorella vulgaris* and *Spirulina platensis* from hospital wastewater. *Desalination Water Treat.* 123 315–320. 10.5004/dwt.2018.22772 26354686

[B290] Yi B. Lu C. Huang W. Yu W. Yang J. Howe A. et al. (2023). Resolving the influence of lignin on soil organic matter decomposition with mechanistic models and continental-scale data. *Glob. Change Biol.* 29 5968–5980. 10.1111/gcb.16875 37448171

[B291] Yin F. Lin S. Zhou X. Dong H. Zhan Y. (2021). Fate of antibiotics during membrane separation followed by physical-chemical treatment processes. *Sci. Total Environ.* 759:143520. 10.1016/j.scitotenv.2020.143520 33248789

[B292] Yu X. Bai M. Li X. Yang P. Wang Q. Wang Z. et al. (2024). Tetracycline removal by immobilized indigenous bacterial consortium using biochar and biomass: Removal performance and mechanisms. *Bioresour. Technol.* 413 131463. 10.1016/j.biortech.2024.131463 39277055

[B293] Zagórska-Dziok M. Ziemlewska A. Nizioł-Łukaszewska Z. Bujak T. (2020). Antioxidant activity and cytotoxicity of *Medicago sativa* L. seeds and herb extract on skin cells. *Biores. Open Access* 9 229–242. 10.1089/biores.2020.0015 33117615 PMC7590823

[B294] Zambrano J. García-Encina P. A. Jiménez J. J. López-Serna R. Irusta-Mata R. (2022). Photolytic and photocatalytic removal of a mixture of four veterinary antibiotics. *J. Water Process Eng.* 48:102841. 10.1016/j.jwpe.2022.102841

[B295] Zampaloni C. Mattei P. Bleicher K. Winther L. Thäte C. Bucher C. et al. (2024). A novel antibiotic class targeting the lipopolysaccharide transporter. *Nature* 625 566–571. 10.1038/s41586-024-07641-4 38172634 PMC10794144

[B296] Zapata-Zúñiga M. C. Parra-Pérez M. Á. Álvarez-Berrio J. A. Molina-Gómez N. I. (2022). Technologies in wastewater treatment plants for the removal of antibiotics, resistant bacteria and antibiotic resistance genes: A review of the current literature*. *Ingenieria Univ.* 26 1–30. 10.11144/Javeriana.iyu26.twtp

[B297] Zhang J. Li X. Klümper U. Lei H. Berendonk T. U. Guo F. et al. (2022). Deciphering chloramphenicol biotransformation mechanisms and microbial interactions via integrated multi-omics and cultivation-dependent approaches. *Microbiome* 10:180. 10.1186/s40168-022-01361-5 36280854 PMC9590159

[B298] Zhang X. Zhao B. Liu Y. Yao M. Li Y. Wu N. (2024). Enhanced effect of sonochemistry on the degradation of trace antibiotics in water by N-β-rGO/PMS adsorption-catalytic oxidation system. *Appl. Catal. O Open* 194:207003. 10.1016/j.apcato.2024.207003

[B299] Zhang Y. Bai J. Zhang L. Zhang C. Liu B. Hu Y. (2021). Self-resistance in the biosynthesis of fungal macrolides involving cycles of extracellular oxidative activation and intracellular reductive inactivation. *Angew. Chem. Int. Ed.* 60 6639–6645. 10.1002/anie.202015442 33314510

[B300] Zhang Y. Liu F. Zhong L. Dong Z. Chen C. Xu Z. (2023). Reusable and environmentally friendly cellulose nanofiber/titanium dioxide/chitosan aerogel photocatalyst for efficient degradation of tetracycline. *Appl. Surface Sci.* 641:158425. 10.1016/j.apsusc.2023.158425

[B301] Zhang Y. Mu T. Huang M. Chen G. Cai T. Chen H. et al. (2020). Nanofiber composite forward osmosis (NCFO) membranes for enhanced antibiotics rejection: Fabrication, performance, mechanism, and simulation. *J. Membr. Sci.* 595:117425. 10.1016/j.memsci.2019.117425

[B302] Zhao K. Padervand M. Ren H. Jia T. Guo Q. Yang L. et al. (2024). Enhancing tetracycline removal efficiency through ozone micro–nano bubbles: Environmental implication and degradation pathway. *ACS Es&t Eng.* 4 1860–1870. 10.1021/acsestengg.4c00102

[B303] Zhao X. Su H. Xu W. Hu X. Xu Y. Wen G. et al. (2021). Removal of antibiotic resistance genes and inactivation of antibiotic-resistant bacteria by oxidative treatments. *Sci. Total Environ.* 778:146348. 10.1016/j.scitotenv.2021.146348 34030387

[B304] Zhao Y. Zhao Q. Liu D. Xie H. Zhang J. Zheng Y. et al. (2024). Antibiotic resistomes and ecological risk elimination in field-scale constructed wetland revealed by integrated metagenomics and metatranscriptomics. *J. Hazard. Mater.* 480:136045. 10.1016/j.jhazmat.2024.136045 39368357

[B305] Zheng S. Wang Y. Chen C. Zhou X. Liu Y. Yang J. et al. (2022). Current progress in natural degradation and enhanced removal techniques of antibiotics in the environment: A review. *Int. J. Environ. Res. Public Health* 19:10919. 10.3390/ijerph191710919 36078629 PMC9518397

[B306] Zhong Y. Guo J. Zheng Y. Lin H. Su Y. (2024). Metabolomics analysis of the Lactobacillus plantarum ATCC 14917 response to antibiotic stress. *BMC Microbiol.* 24:229. 10.1186/s12866-024-03385-3 38943061 PMC11212188

[B307] Zhou S. Shao Y. Gao N. Zhu S. Ma Y. Deng J. (2014). Chlorination and chloramination of tetracycline antibiotics: Disinfection by-products formation and influential factors. *Ecotoxicol. Environ. Saf.* 107 30–35. 10.1016/j.ecoenv.2014.05.008 24905694

[B308] Zhou T. An Q. Zhang L. Wen C. Yan C. (2024). Phytoremediation for antibiotics removal from aqueous solutions: A meta-analysis. *Environ. Res.* 240:117516. 10.1016/j.envres.2023.117516 37890821

[B309] Zhou Y. Gao Y. Pang S. Y. Jiang J. Yang Y. Ma J. et al. (2018). Oxidation of fluoroquinolone antibiotics by peroxymonosulfate without activation: Kinetics, products, and antibacterial deactivation. *Water Res.* 145 210–219. 10.1016/j.watres.2018.08.026 30142519

[B310] Zhu K. Li X. Chen Y. Huang Y. Yang Z. Guan G. et al. (2024). Recent advances on the spherical metal oxides for sustainable degradation of antibiotics. *Coord. Chem. Rev.* 510:215813. 10.1016/j.ccr.2024.215813

[B311] Zhuang Y. Ren H. Geng J. Zhang Y. Zhang Y. Ding L. et al. (2015). Inactivation of antibiotic resistance genes in municipal wastewater by chlorination, ultraviolet, and ozonation disinfection. *Environ. Sci. and Pollut. Res.* 22 7037–7044. 10.1007/s11356-014-3919-z 25483976

[B312] Zouli N. I. (2024). Photodegradation of a broad-spectrum antibiotic azithromycin using H2O2 under ultraviolet irradiation. *Int. J. Mol. Sci.* 25:6702. 10.3390/ijms25126702 38928406 PMC11203608

[B313] Zwama M. Hayashi K. Sakurai K. Nakashima R. Kitagawa K. Nishino K. et al. (2017). Hoisting-loop in bacterial multidrug exporter AcrB is a highly flexible hinge that enables the large motion of the subdomains. *Front. Microbiol.* 8:2095. 10.3389/fmicb.2017.02095 29118749 PMC5661021

[B314] Żyłła R. Ledakowicz S. Boruta T. Olak-Kucharczyk M. Foszpańczyk M. Mrozińska Z. et al. (2021). Removal of tetracycline oxidation products in the nanofiltration process. *Water* 13:555. 10.3390/w13040555

